# Supplementary material for: CDK8 and CDK19 act redundantly to control the CFTR pathway in the intestinal epithelium
Source: EMBO Rep. 2022 Dec 22;24(2):e54261. doi: 10.15252/embr.202154261 (PMC10549226; doi:10.15252/embr.202154261)
Supplement: Supplementary file 1 — Appendix [file EMBR-24-e54261-s006.pdf]

Appendix for:
CDK8 and CDK19 act redundantly to control the CFTR pathway in the intestinal epithelium

Contents .... Page

Appendix Fig S1. CDK8 loss does not affect chemically-induced intestinal carcinogenesis........... 2

Appendix Fig S2. Amino acid sequence conservation of CDK8 and its paralogue CDK19 between different vertebrates.. 3

Appendix Fig S3. Cdk8/Cdk19 KO organoids are counter-selected. 4

Appendix Fig S4. Cdk8/Cdk19 double knockout does not significantly affect intestinal cell differentiation. 5

Appendix Fig S5. CDK8/19 inhibition is well tolerated by mice. 6

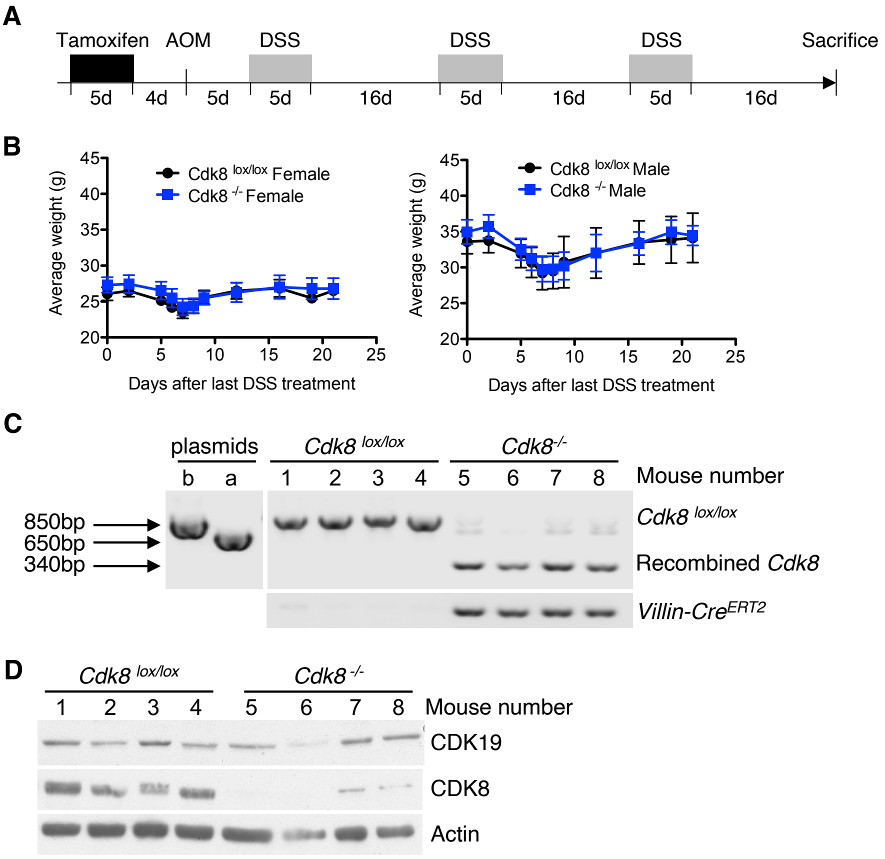

Appendix Fig S1. CDK8 loss does not affect chemically-induced intestinal carcinogenesis.

A Scheme showing the steps of the AOM/DSS carcinogenesis experiment. See Materials and Methods for more detailed information.

B Graphs showing female (left; n=6 mice in both groups*)* and male (right; n=5 mice in both groups) weight evolution over 21 days following the last DSS treatment.

C Genotyping after AOM/DSS treatment confirms the recombination and loss of *Cdk8* exon 2 in colon tumors from *Cdk8* $^{-/-}$ mice. Control plasmids (a and b), are described in Fig EV1C. PCR amplification with *Villin-Cre*^{ERT2}-specific primers confirms the presence of the *Cre*^{ERT2} recombinase gene.

D WB with the same colon tumour samples presented in (C) confirm the disappearance of CDK8 protein in the *Cdk8* $^{-/-}$ mice. β-actin was used as the loading control.

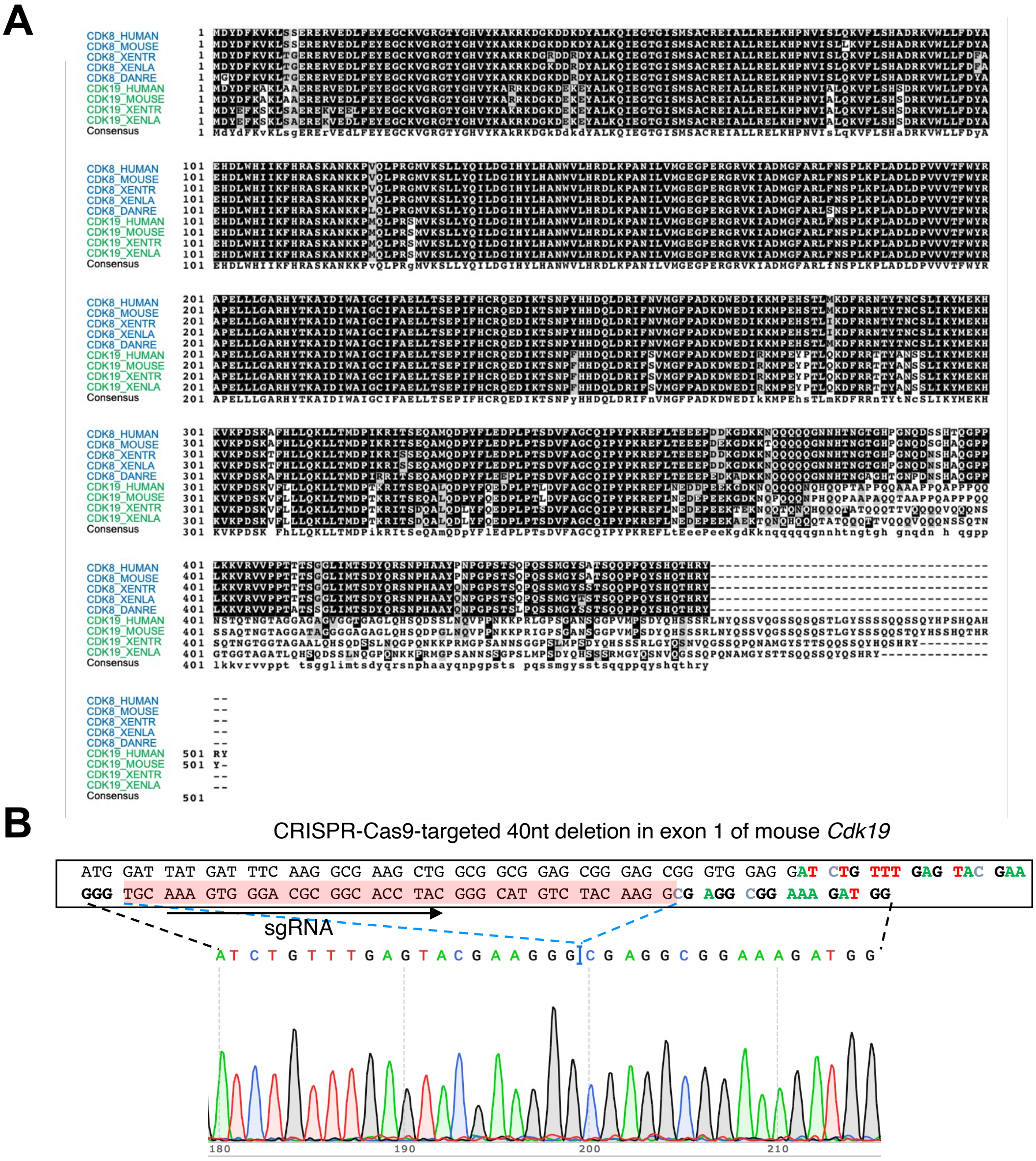

Appendix Fig S2. Amino acid sequence conservation of CDK8 and its paralogue CDK19 between different vertebrates.

A Sequence alignment of Cdk8 (blue) and Cdk19 (green) proteins from: *Homo sapiens, Mus musculus, Xenopus tropicalis, Xenopus laevis, and Danio rerio.* Homologous sequences are black. The consensus (> 80%) is presented below the alignment.

B Scheme indicating the fragment of *Cdk19* exon 1 removed by CRISPR-Cas9 (highlighted in red) in intestinal organoids. The arrow indicates the sequence of the sgRNA used. The sequence trace obtained after gene editing is presented below. The colour-code in the sequence in the box corresponds to the sequence trace.

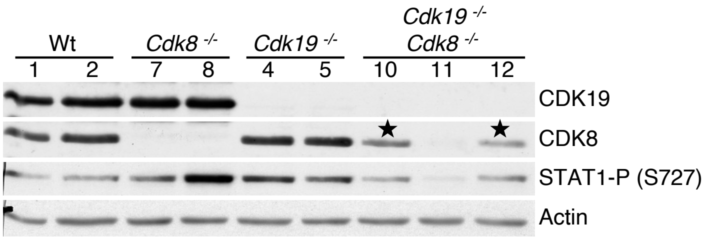

Appendix Fig S3. Cdk8/Cdk19 KO organoids are counter-selected.

WB indicating the levels of CDK8, CDK19 and phospho-STAT1-S727 in organoids after 14 days of OH-tamoxifen treatment. Two out of the three *Cdk8* $^{-/-}$/*Cdk19* $^{-/-}$ clones (numbers 10 and 12) show a reappearance of the CDK8 protein: compare with Fig. 2B where proteins were extracted from the same samples, but one week earlier. (★) indicates the two clones where CDK8 protein is detected; this was observed only in organoids where double KO had been induced.

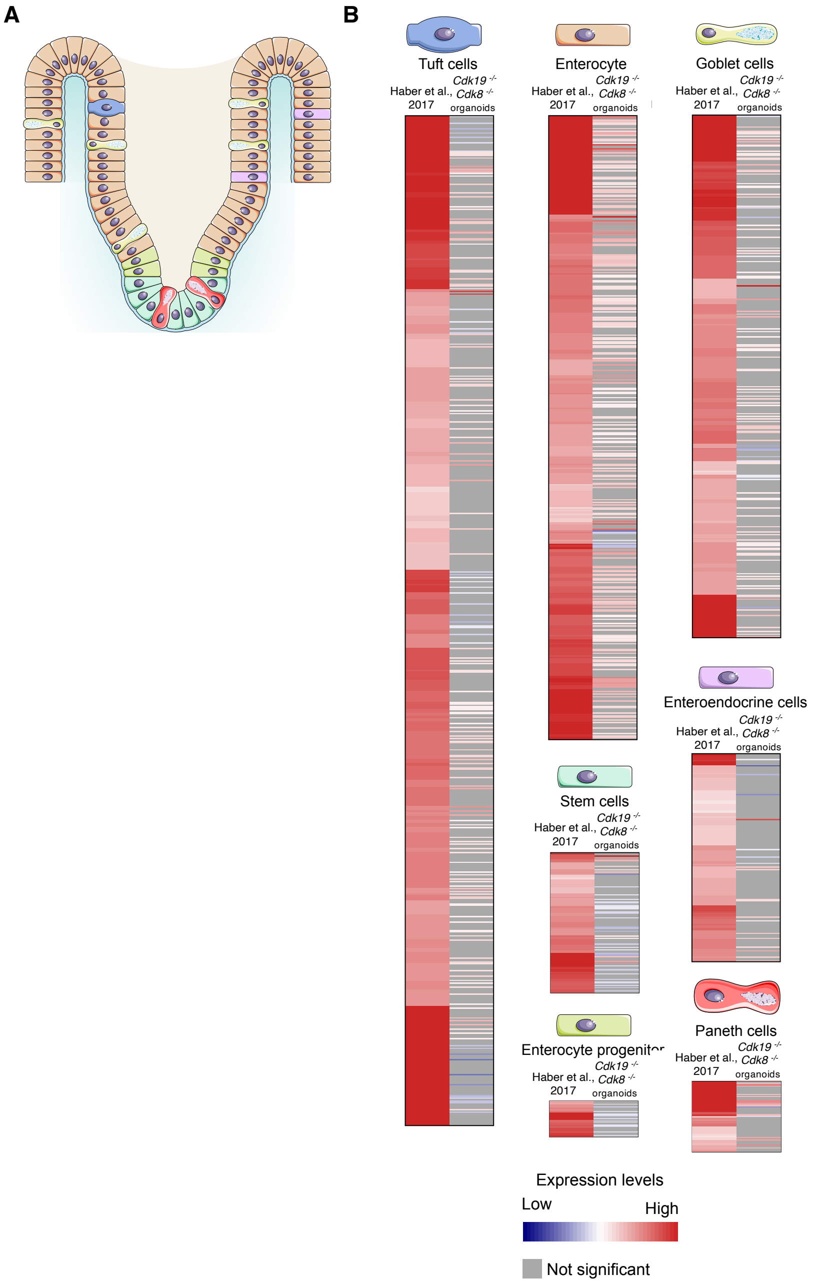

Appendix Fig S4. Cdk8/Cdk19 double knockout does not significantly affect intestinal cell differentiation.

A Schematic representation of the intestinal crypt and villus with all cell types indicated.

B Heatmaps visualising differences in gene expression in double *Cdk8/Cdk19* knockout organoids (bulk RNA-seq) and in indicated intestinal cell types obtained by single-cell RNAseq (Haber et al., 2017). After clustering and identifying the different cell types, gene signatures were defined as those genes in a given cell type that were significantly more expressed when compared against all other cell types, in a pairwise manner. Most signature genes for each cell type are not significantly deregulated (grey, right column of heatmaps) in organoids lacking CDK8 and CDK19.

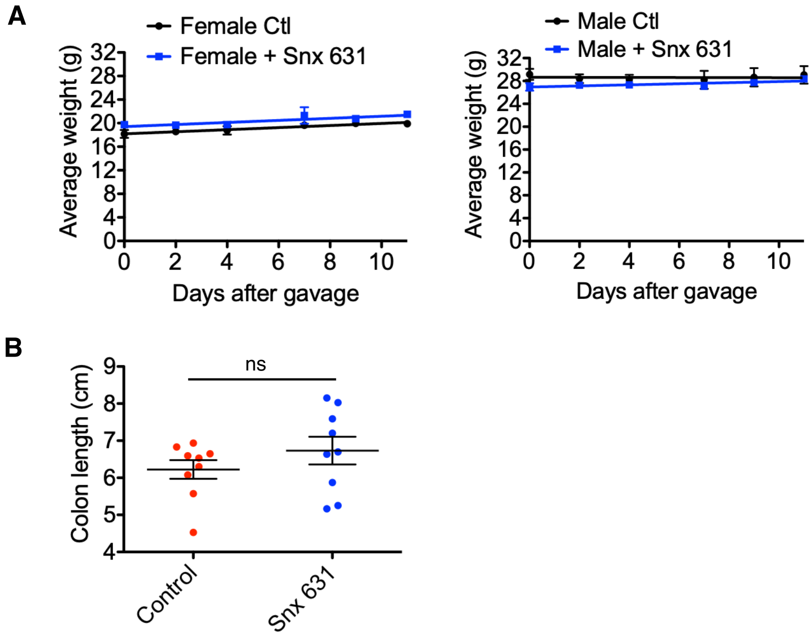

Appendix Fig S5. CDK8/19 inhibition is well tolerated by mice.

A, B Mice were treated with Senexin 631 (Snx 631, n=10) or vehicle (n=10) and mouse weight **(A)** during the experiment, and colon length **(B)** after 11 days of treatment were measured.
