# Supplementary material for: CDK8 and CDK19 act redundantly to control the CFTR pathway in the intestinal epithelium
Source: EMBO Rep. 2022 Dec 22;24(2):e54261. doi: 10.15252/embr.202154261 (PMC10549226; doi:10.15252/embr.202154261)
Supplement: Supplementary file 2 — Expanded View Figures PDF [file EMBR-24-e54261-s005.pdf]

Expanded View Figures

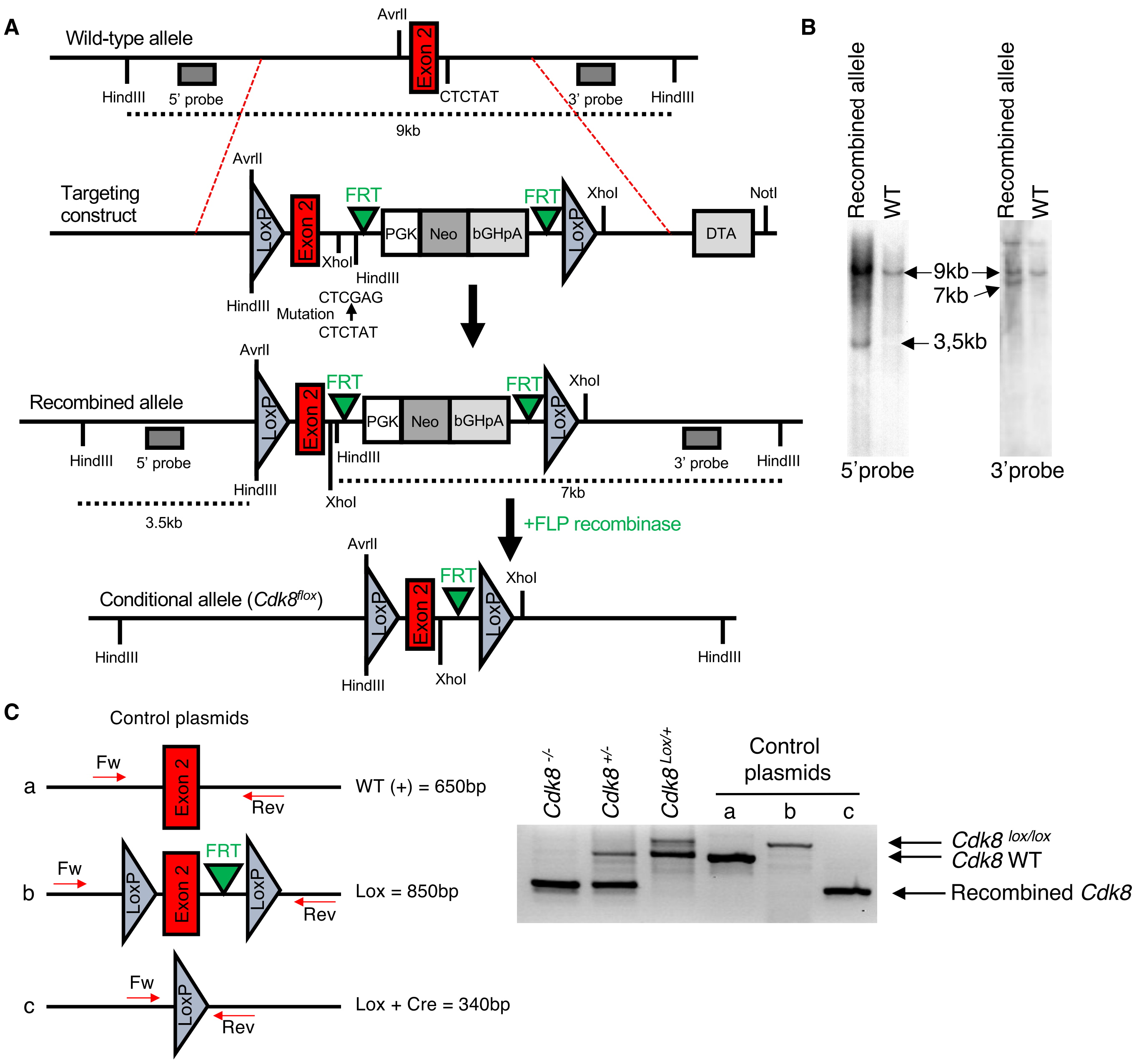

Figure EV1. Mouse *Cdk8* conditional knockout by Lox/Cre targeting of exon 2.

A Schematic representation of the strategy used for the generation of *Cdk8*^{Lox} alleles from a genomic fragment of mouse enclosing exon 2 of the *Cdk8* gene. See Materials and Methods section for details.

B Southern blot analysis of genomic DNA obtained from mice carrying the *Cdk8*^{Lox} and *Cdk8*^{WT} alleles. DNA was digested with HindIII and probed with 2 different probes (5′ and 3′), whose positions are shown in the scheme in (A). The length of the fragments obtained after HindIII digestion of the wild type and the recombinant alleles are indicated (see also the scheme in (A)).

C Left, scheme representing the control plasmids (a, b, and c) for WT, floxed and recombined *Cdk8* exon 2. The position of the oligos (Fw and Rev) used for PCR amplification of genomic DNA and control plasmids is indicated. Right, genotyping of *Cdk8* exon 2 in the mouse intestinal epithelium. All mice were treated with tamoxifen to induce recombination of the LoxP sites. The recombined fragment appears as a 340 bp band in the *Cdk8*$^{-/-}$ and *Cdk8*$^{+/-}$ mice (*VillinCre*^{ERT2} recombinase-positive), and is absent in the *Cdk8*$^{Lox/+}$ mouse that does not contain the *VillinCre*^{ERT2} gene.

© 2022 The Authors

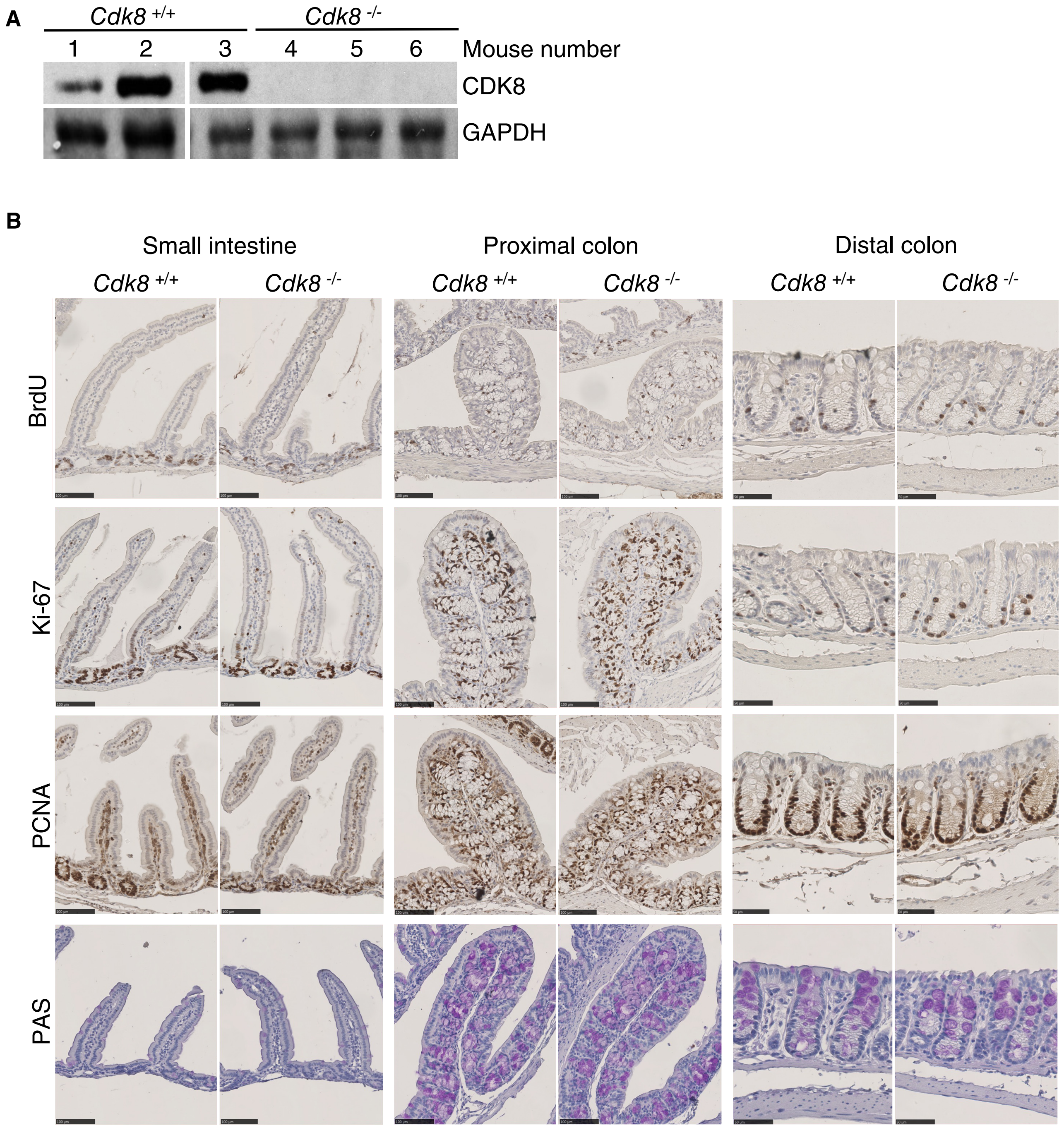

Figure EV2. CDK8 is not required for cell proliferation nor differentiation in mouse intestine.

A WB analysis of mouse intestine epithelium showing the absence of CDK8 protein 2 months after tamoxifen feeding. Mice 1, 2, and 3 did not have the *VillinCre*^{ERT2} gene; mice 4, 5, and 6 had the *VillinCre*^{ERT2}. GAPDH protein was used as loading control.

B Representative immunohistochemistry images of Fig 1B. Small intestine, proximal and distal colon samples were stained for BrdU, Ki-67 or PCNA antibodies. Goblet cells were detected with PAS staining. Scale bars: 100 μm for small intestine and distal colon, 50 μm for proximal colon. Twenty mice were used for the experiment (10 males and 10 females).

Source data are available online for this figure.

 © 2022 The Authors

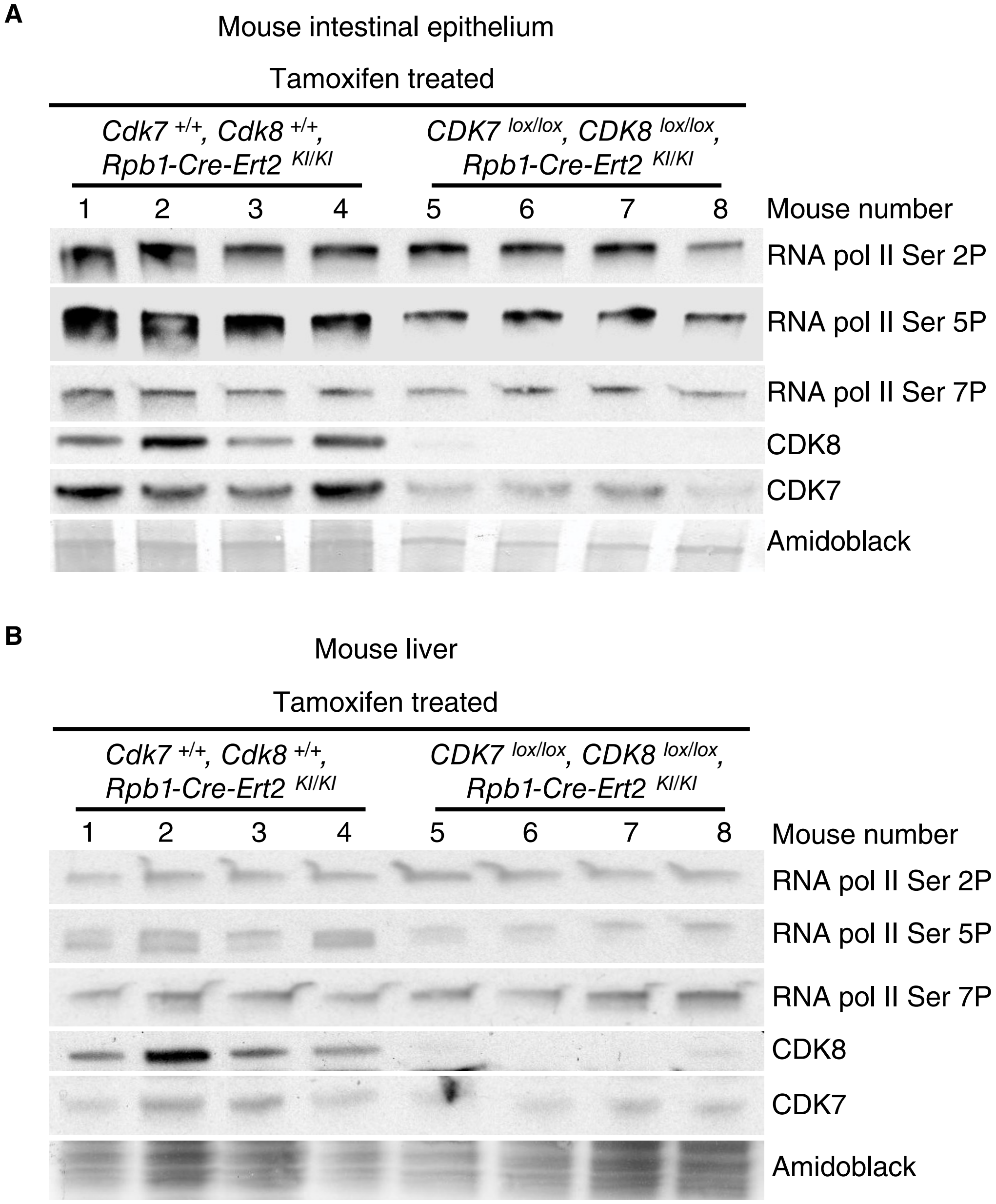

Figure EV3. Effects of CDK8 and CDK7 knockout on RNA pol II CTD phosphorylation.

A, B (A) WB analysis of the indicated proteins in mouse intestinal epithelium samples from WT and *Cdk7*$^{lox/lox}$, *Cdk8*$^{lox/lox}$, *Rpb-Cre-Ert2*$^{KI/KI}$ mice after tamoxifen treatment. Amido-black staining was used as loading control. Sixteen mice of each genotype (eight males and eight females) were used for this experiment. (B) As A but for liver.

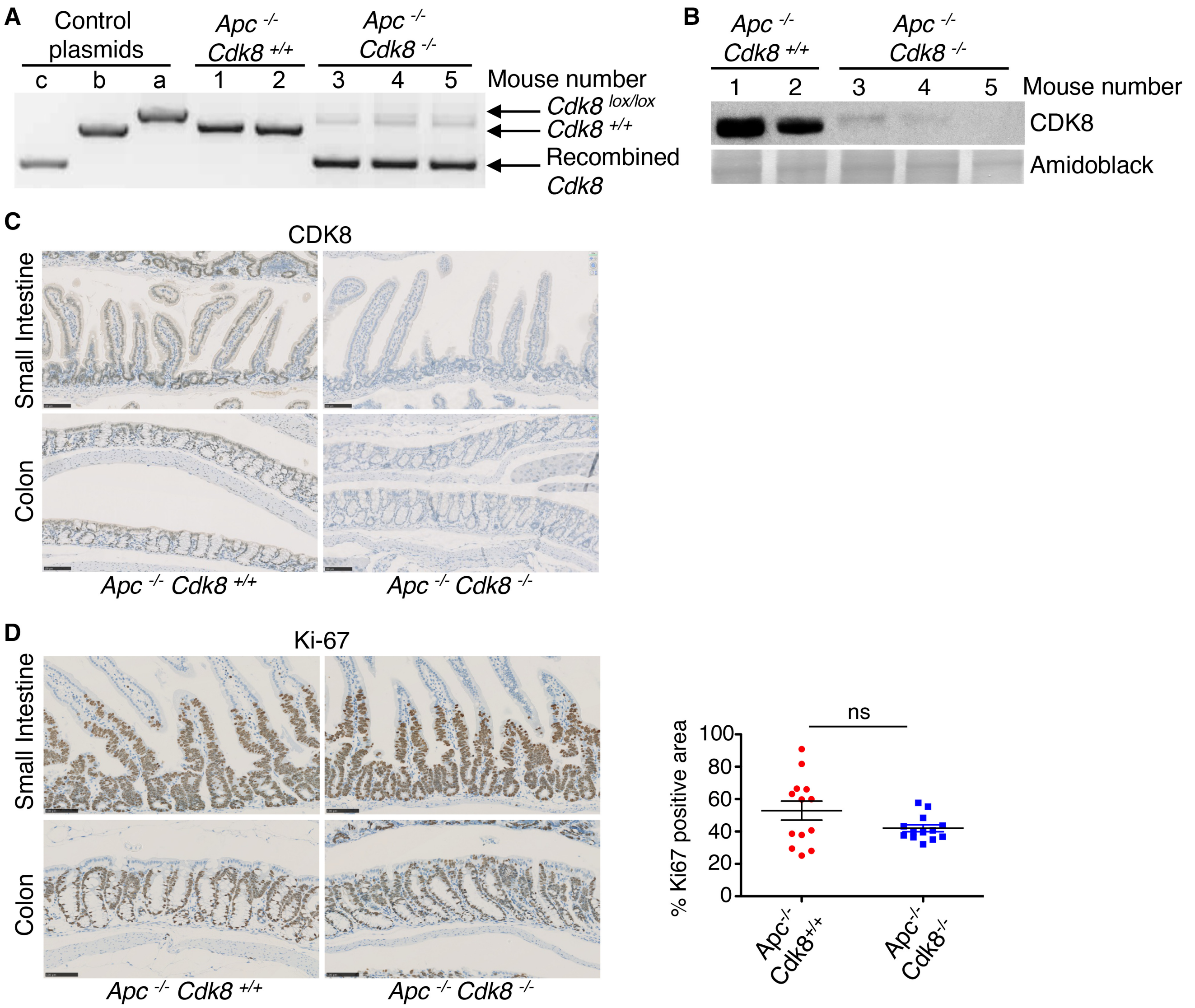

Figure EV4. CDK8 deletion does not prevent Apc-loss-dependent tumourigenesis in mouse intestine.

A Genotyping confirms the loss of *Cdk8* exon 2 in intestine epithelium from *Apc*$^{-/-}$/*Cdk8*$^{-/-}$ mice. Control plasmids (a, b, and c) are described in Fig EV1C.

B WB with intestine epithelium samples from mice presented in (A) confirm the absence of CDK8 protein in *Cdk8*$^{-/-}$ mice. Amido-black staining was used as loading control.

C, D IHC staining of (C) CDK8 and (D) Ki-67 in small intestine and colon samples from *Apc*$^{-/-}$/*Cdk8*$^{+/+}$ and *Apc*$^{-/-}$/*Cdk8*$^{-/-}$ mice. Scale bars, 100 μm. (D), right: quantification of the Ki-67 positive area (% of the total area of the intestine presenting positive staining, quantified using QuPath (Bankhead *et al*, 2017) and Image J software; mean ± SD). Two-tailed *P*-value of unpaired *t*-test is indicated: ns, not significant ($P > 0.05$; $n = 6$ biological replicates).

Figure EV5. CDK8 and CDK19 are largely redundant but regulate a subset of genes in an opposite manner.

A Comparison of the Log 2 of estimated fold change in expression for each individual gene using a spike-in normalisation method vs. the internal Deseq2 normalisation (library-normalised). From left to right the results for *Cdk8*$^{-/-}$, *Cdk19*$^{-/-}$, and *Cdk8*$^{-/-}$/*Cdk19*$^{-/-}$ organoids. Each black dot represents a gene; the dashed red line indicates the identity diagonal; in blue, the linear regression of the data described by the equation shown in the plot.

B, C Standard deviation from the replicates of each gene (B) and mean of the normalised read counts of each gene (C) in the single *Cdk8* and *Cdk19* knockouts vs the *Cdk8/Cdk19* double knockout. Each grey point represents a gene; genes differentially expressed only in a single knockout are highlighted in black. The identity diagonal is represented as a red dashed line.

D, E PCA (Principal component analysis) plots displaying the two axes that explain 72 and 77% of the variance between differentially expressed genes in *Cdk8* knockout (D) or *Cdk19* knockout (E), respectively, and the other genotypes.

 © 2022 The Authors

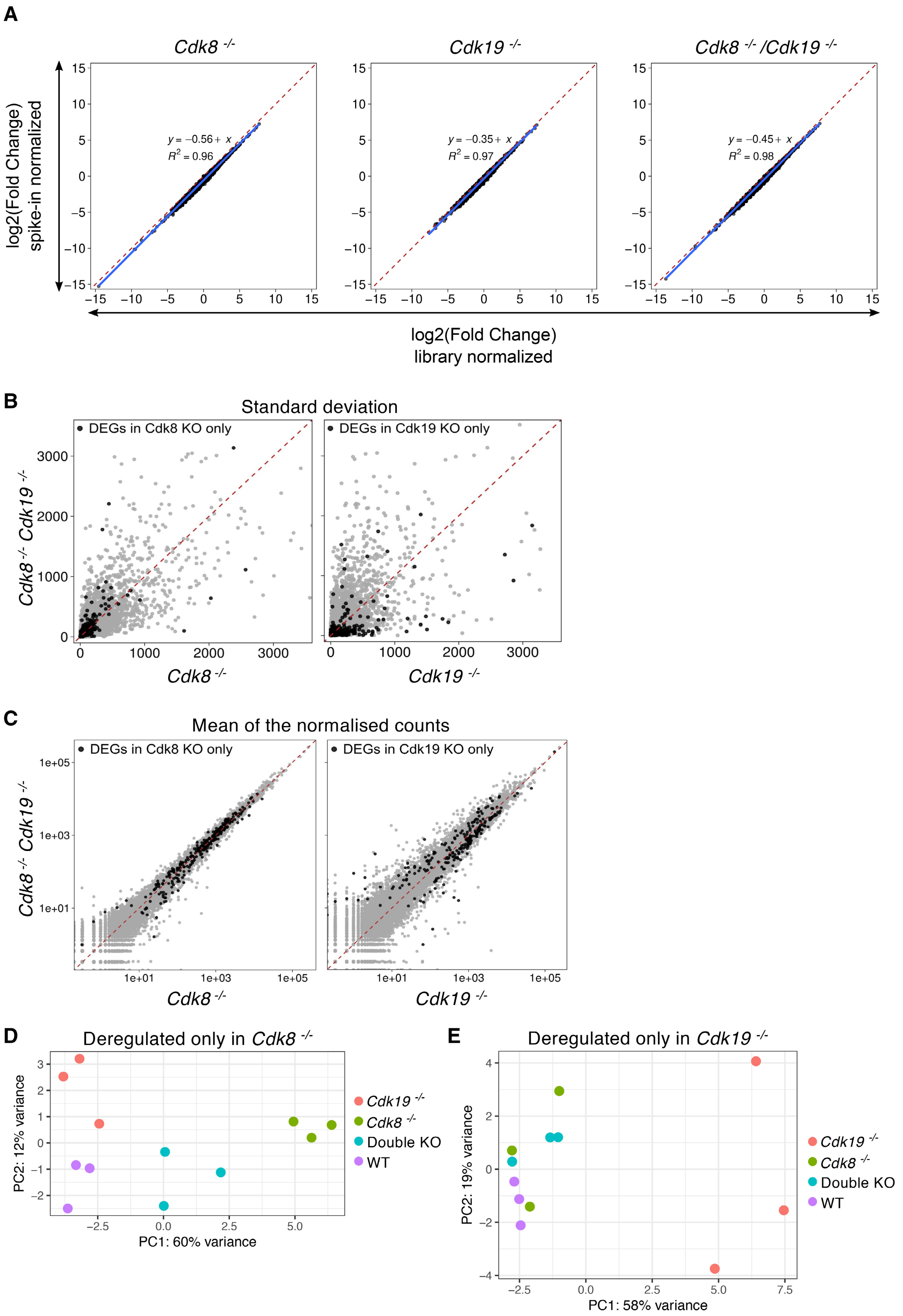
A
Cdk8 -/-
Cdk19 -/-
Cdk8 -/- /Cdk19 -/-
y = −0.56 + x
R2 = 0.96
y = −0.35 + x
R2 = 0.97
y = −0.45 + x
R2 = 0.98
log2(Fold Change)
spike-in normalized
log2(Fold Change)
library normalized
B
Standard deviation
DEGs in Cdk8 KO only
DEGs in Cdk19 KO only
Cdk8 -/- Cdk19 -/-
Cdk8 -/-
Cdk19 -/-
C
Mean of the normalised counts
DEGs in Cdk8 KO only
DEGs in Cdk19 KO only
Cdk8 -/- Cdk19 -/-
Cdk8 -/-
Cdk19 -/-
D
Deregulated only in Cdk8 -/-
PC2: 12% variance
PC1: 60% variance
Cdk19 -/-
Cdk8 -/-
Double KO
WT
E
Deregulated only in Cdk19 -/-
PC2: 19% variance
PC1: 58% variance
Cdk19 -/-
Cdk8 -/-
Double KO
WT

Figure EV5.
