# Supplementary material for: CDK8 and CDK19 act redundantly to control the CFTR pathway in the intestinal epithelium
Source: EMBO Rep. 2022 Dec 22;24(2):e54261. doi: 10.15252/embr.202154261 (PMC10549226; doi:10.15252/embr.202154261)
Supplement: Supplementary file 5 — Movie EV1 [file EMBR-24-e54261-s004.zip › Movie_EV1/EMBOR-2021-54261V4_MovieEV1_legend.docx]

**Movie EV1.** Live-cell microscopy shows a rapid expansion of both the lumen and total organoid surface area in WT organoids after the addition of forskolin. *Cdk8^-/-^/Cdk19^-/-^* organoids do not swell after forskolin addition. Three different sizes of organoids are presented in each condition: big, (top); medium (middle) and small (bottom). Scale bar, 200 μm.
