# Supplementary material for: CDK8 and CDK19 act redundantly to control the CFTR pathway in the intestinal epithelium
Source: EMBO Rep. 2022 Dec 22;24(2):e54261. doi: 10.15252/embr.202154261 (PMC10549226; doi:10.15252/embr.202154261)
Supplement: Supplementary file 9 — Source Data for Expanded View and Appendix [file EMBR-24-e54261-s007.zip › EMBR_2333_EMBOR202154261V3_Source_data_AppendixFigS3.pdf]

Appendix Figure S3

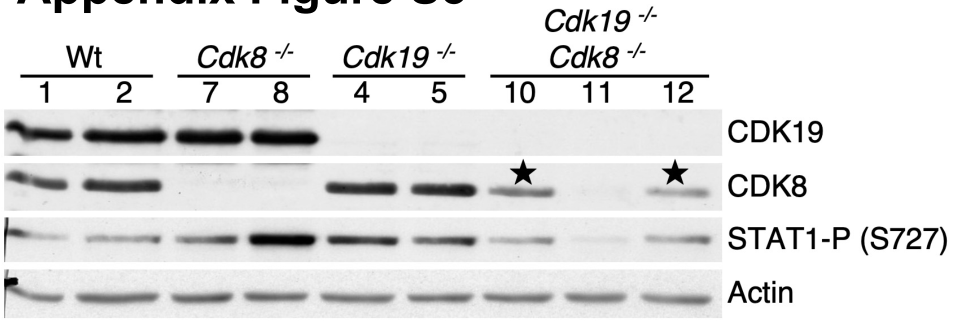

Original films for this figure

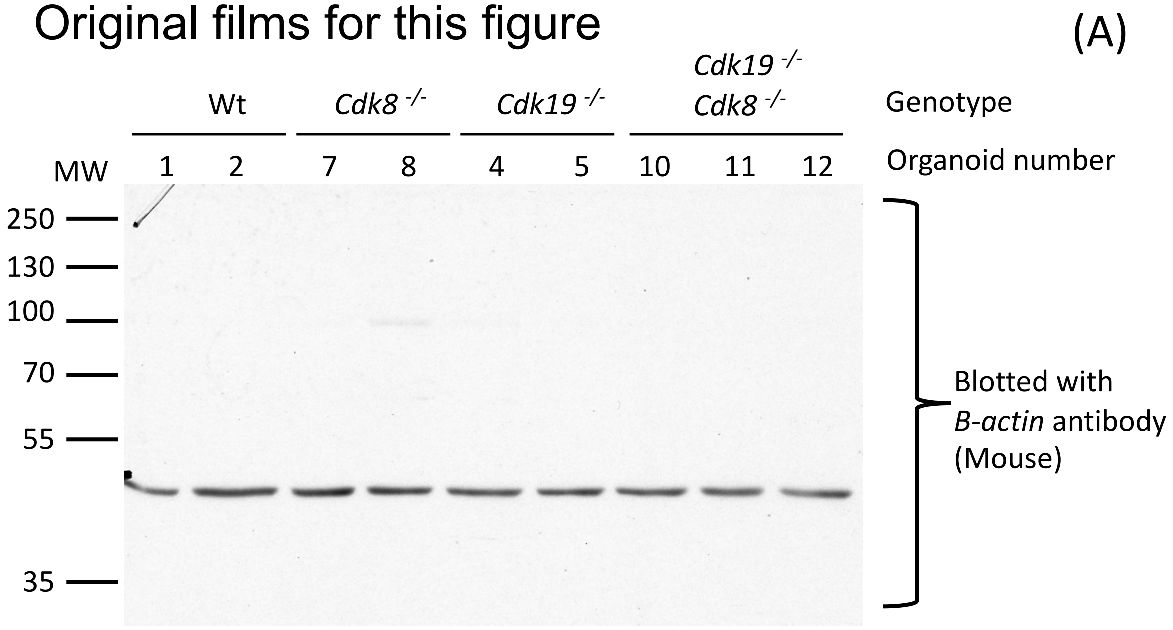

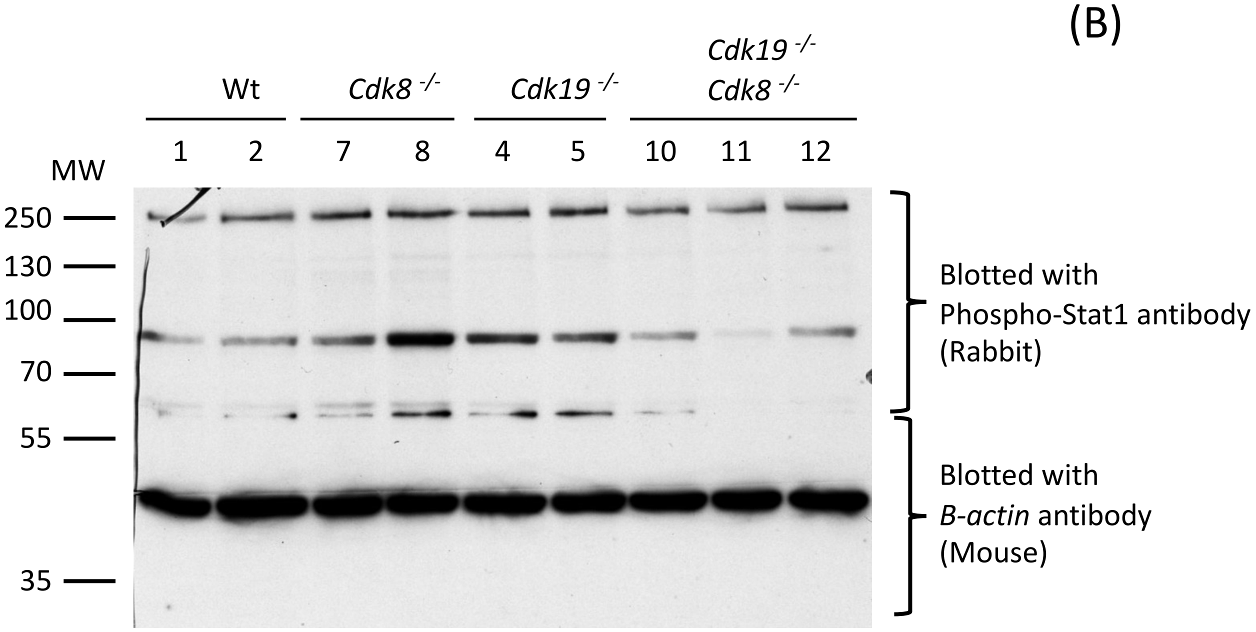

(C)

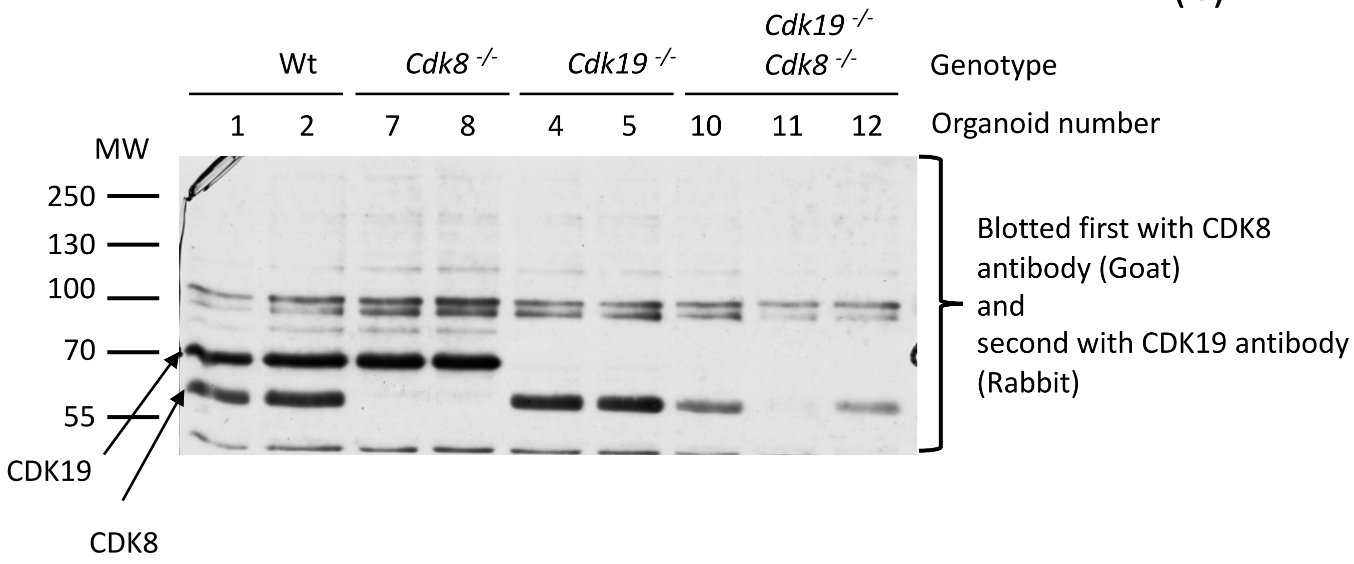

Samples presented in Appendix Figure S3 come from 2 different gels. The same volume of each sample
was loaded in each gel and both gels were migrated in parallel. Proteins from each gel were transferred to 2 different membranes. Membrane (A) was first blotted with *B*-actin antibody, and secondly with phosphor-Stat1 antibody (B). The second membrane (C) containing the same samples and loaded in the same order as the first one was first blotted with CDK8 antibody (Goat) and secondly with CDK19 antibody (Rabbit). Numbers indicated on top of each membrane correspond to the organoids numbers indicated in Appendix Fig. S3.
