# Supplementary material for: CDK8 and CDK19 act redundantly to control the CFTR pathway in the intestinal epithelium
Source: EMBO Rep. 2022 Dec 22;24(2):e54261. doi: 10.15252/embr.202154261 (PMC10549226; doi:10.15252/embr.202154261)
Supplement: Supplementary file 9 — Source Data for Expanded View and Appendix [file EMBR-24-e54261-s007.zip › EMBR_2333_EMBOR202154261V3_Source_data_EV2A.pdf]

Figure EV2.

A

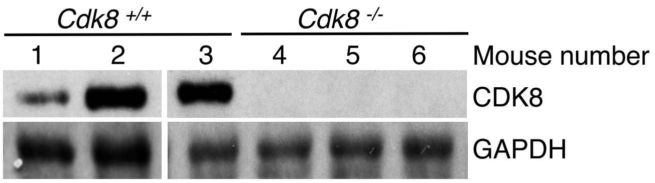

Original films for this figure

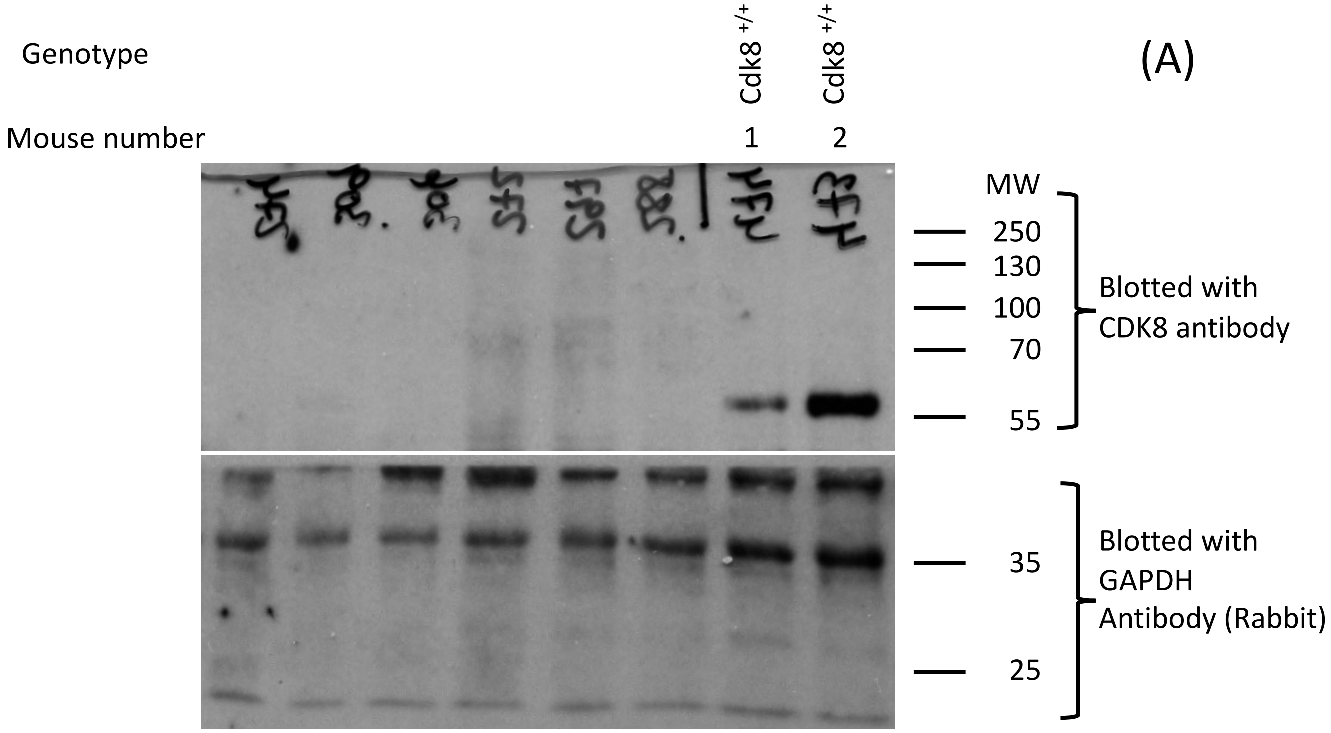

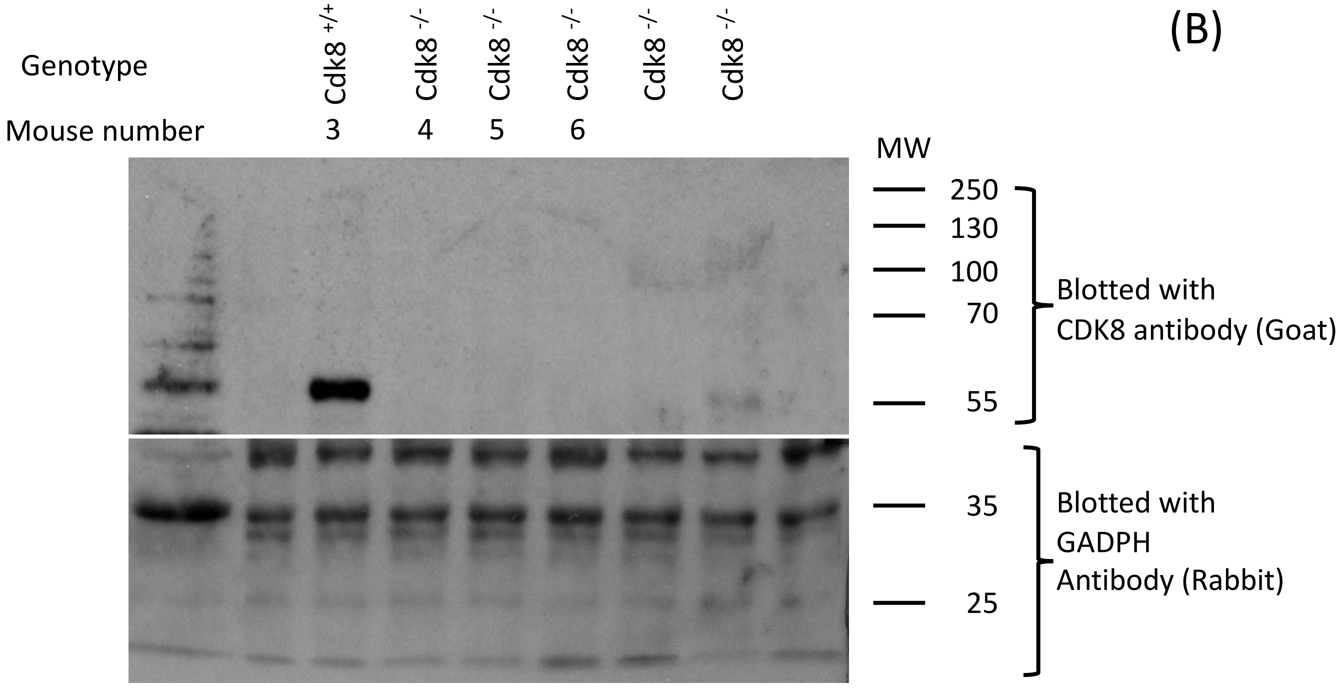

Samples presented in Figure EV2A come from 2 different membranes. A white vertical lane has been drawn between sample 2 and 3 in the final figure EV2A to indicate that samples on the left of the white lane come from one gel and samples on the right of the lane come from another gel.
Each membrane was divided into 2 parts. The upper part of each membrane was blotted with CDK8 antibody and the bottom part was blotted with GADPH antibody. To create Figure EV2A samples 1 and 2 were taken from the top membrane (A) and samples 3, 4, 5 and 6 were taken from bottom membrane (B). Numbers indicated on top of each membrane correspond to the mouse numbers indicated in Fig. EV2-A.
