# Supplementary material for: CDK8 and CDK19 act redundantly to control the CFTR pathway in the intestinal epithelium
Source: EMBO Rep. 2022 Dec 22;24(2):e54261. doi: 10.15252/embr.202154261 (PMC10549226; doi:10.15252/embr.202154261)
Supplement: Supplementary file 10 — PDF+ [file EMBR-24-e54261-s001.pdf]

Article

SOURCE DATA TRANSPARENT PROCESS OPEN ACCESS

EMBO reports

CDK8 and CDK19 act redundantly to control the CFTR pathway in the intestinal epithelium

Susana Prieto[1,2], Geronimo Dubra[1,2], Alain Camasses[1,2], Ana Bella Aznar[1,2], Christina Begon-Pescia[1,†], Elisabeth Simboeck[1,2,‡], Nelly Pirot[4,5], François Gerbe[2,3], Lucie Angevin[1,2], Philippe Jay[2,3], Liliana Krasinska[1,2] & Daniel Fisher[1,2,*]

Abstract

CDK8 and CDK19 form a conserved cyclin-dependent kinase subfamily that interacts with the essential transcription complex, Mediator, and also phosphorylates the C-terminal domain of RNA polymerase II. Cells lacking either CDK8 or CDK19 are viable and have limited transcriptional alterations, but whether the two kinases redundantly control cell proliferation and differentiation is unknown. Here, we find in mice that CDK8 is dispensable for regulation of gene expression, normal intestinal homeostasis, and efficient tumourigenesis, and is largely redundant with CDK19 in the control of gene expression. Their combined deletion in intestinal organoids reduces long-term proliferative capacity but is not lethal and allows differentiation. However, double-mutant organoids show mucus accumulation and increased secretion by goblet cells, as well as downregulation of expression of the cystic fibrosis transmembrane conductance regulator (CFTR) and functionality of the CFTR pathway. Pharmacological inhibition of CDK8/19 kinase activity in organoids and in mice recapitulates several of these phenotypes. Thus, the Mediator kinases are not essential for cell proliferation and differentiation in an adult tissue, but they cooperate to regulate specific transcriptional programmes.

Keywords CDK19; CDK8; CFTR; intestinal epithelium; Mediator
Subject Categories Chromatin; Transcription; Genomics
DOI 10.15252/embr.202154261 | Received 2 November 2021 | Revised 28 November 2022 | Accepted 28 November 2022 | Published online 22 December 2022
EMBO Reports (2023) 24: e54261

Introduction

CDK8 was discovered as a kinase that binds cyclin C and, like CDK7-cyclin H and CDK9-cyclin T, can promote transcription by phosphorylating the C-terminal repeat domain (CTD) of RNA polymerase II (Pol II; Rickert *et al*, 1999). CDK8 and cyclin C are exceptionally highly conserved in vertebrates, as illustrated by 97% and 98% amino acid identity over the whole sequence between *Xenopus* and human CDK8 and cyclin C, respectively. This unusual level of cross-species conservation implies critical functions in fundamental cellular processes. In complex with Med12 and Med13, CDK8-cyclin C forms the canonical cyclin-dependent kinase module (CKM) of the Mediator transcriptional co-regulator complex, a function that is conserved with the more divergent yeast homologues of CKM subunits (Jeronimo *et al*, 2016). The latter were revealed as suppressors of a CTD truncation, suggesting a transcription-repressive activity of the CKM (Liao *et al*, 1995). In vertebrates, a second member of the CDK8 subfamily, CDK19, almost identical in the kinase domain with CDK8, also binds cyclin C and interacts with Mediator, in a manner generally thought to be exclusive with CDK8 (Sato *et al*, 2004; Tsutsui *et al*, 2008; Knuesel *et al*, 2009).

Mediator is a large multi-subunit complex required for Pol II-dependent transcription in all eukaryotes (Malik & Roeder, 2010). Acute ablation of vertebrate Mediator is lethal for cells and results in a rapid downregulation of the entire transcriptome (El Khattabi *et al*, 2019). The tail subunits integrate enhancer-associated transcription factor activity into conformational changes of the head and middle complex, which controls Pol II interactions with the basal transcriptional machinery at promoters as well as phosphorylation of the CTD (Malik & Roeder, 2010). Biochemical analysis in yeast provided evidence that the CKM negatively regulates Mediator. *In vitro* experiments suggest that it hinders basal transcription by sterically blocking CTD-dependent recruitment of Pol II to Mediator middle subunits (Elmlund *et al*, 2006; Tsai *et al*, 2013); while *in vivo* data show that the CKM binds to the same promoters as core Mediator but with low stoichiometry (Andrau *et al*, 2006). This appears to be due to negative regulation of Mediator binding to upstream enhancer sequences and release of the CKM module upon Mediator-Pol II interactions (Jeronimo *et al*, 2016).

1 IGMM, University of Montpellier, CNRS, Inserm, Montpellier, France
2 Equipe Labellisée LIGUE 2018, Ligue Nationale Contre le Cancer, Paris, France
3 IGF, University of Montpellier, CNRS, Inserm, Montpellier, France
4 IRCM, University of Montpellier, ICM, INSERM, Montpellier, France
5 BioCampus, RHEM, University of Montpellier, CNRS, INSERM, Montpellier, France
*Corresponding author. Tel: +33 434 359 694; E-mail: daniel.fisher@igmm.cnrs.fr
†Present address: LPHI, University of Montpellier, Montpellier, France
‡Present address: UAS Technikum Wien, Vienna, Austria

© 2022 The Authors. Published under the terms of the CC BY 4.0 license.

In contrast to Mediator, the activity of the CKM is apparently non-essential in many cell types, as genes encoding CDK8, CDK19, and cyclin C are not required for survival and proliferation of most cell types in different organisms (Kuchin *et al*, 1995; Loncle *et al*, 2007; Li *et al*, 2014; Postlmayr *et al*, 2020). However, CDK8 is required for normal development. Germline ablation of *Cdk8* is lethal at the pre-implantation stage in mice (Westerling *et al*, 2007), while conditional deletion using a *Sox2* Cre driver is lethal around embryonic day 10.5 (Postlmayr *et al*, 2020). The difference in lethality stage between the two genotypes suggests that CDK8 might be essential in zygotes, prior to *Sox2* expression.

Deletions of other CKM subunits in mice have variable phenotypes. Cyclin C gene deletion is embryonic lethal at day 10.5 with severe growth defects, and its deletion in adults affects T-cell differentiation (Li *et al*, 2014), while deletion of *Med12* is lethal at late embryonic stages, preventing neural-tube closure, axis elongation, and organ morphogenesis (Rocha *et al*, 2010). Mice with a CRISPR-mediated CDK19 deletion were recently reported to be viable and generally healthy (Dannappel *et al*, 2022). An essential requirement for CKM subunits in transcriptional regulation in animals cannot, however, be completely ruled out, since differences in the lethality stage of CKM subunit deletions could be due to differential maternal mRNA contributions.

Consistent with a repressive role for the CKM in transcription, we recently reported that inhibition of CDK8 and CDK19 in human and mouse pluripotent stem cells is associated with a global overactivation of enhancers and a stabilisation of the naive state (Lynch *et al*, 2020). Similarly, in acute myeloid leukaemia, CDK8/19 bind superenhancers and their chemical inhibition further activates enhancer activity (Pelish *et al*, 2015).

CDK8 has been attributed oncogenic functions in different cancers, including Wnt-dependent colorectal cancer, melanoma, breast and prostate cancer, acute myeloid leukaemia, and B-cell leukaemia (Firestein *et al*, 2008; Morris *et al*, 2008; Kapoor *et al*, 2010; Pelish *et al*, 2015; McDermott *et al*, 2017; Nakamura *et al*, 2018; Menzl *et al*, 2019). Originally proposed to act in intestinal cancers by promoting Wnt transcription (Firestein *et al*, 2008), CDK8 is also involved in transcription dependent on Notch signalling (Li *et al*, 2014), NFκB (Chen *et al*, 2017), HIF1α (Galbraith *et al*, 2013), the serum-response (Donner *et al*, 2010), the interferon-γ response (Bancerek *et al*, 2013; Steinparzer *et al*, 2019), p53 (Donner *et al*, 2007), superenhancers (Pelish *et al*, 2015), histone variant incorporation into chromatin (Kapoor *et al*, 2010), in pluripotency maintenance (Adler *et al*, 2012), and the senescence-associated tumour-promoting secretory phenotype (Porter *et al*, 2012). It also restrains NK-mediated cell toxicity and tumour surveillance (Hofmann *et al*, 2020). CDK8 is thought to act either by directly phosphorylating transcription factors, such as Notch1 (Li *et al*, 2014) or STAT1 (Bancerek *et al*, 2013), or by being co-recruited to promoters via specific transcription factors, where it phosphorylates Pol II CTD (Galbraith *et al*, 2013; Chen *et al*, 2017; Steinparzer *et al*, 2019). Yet genetic confirmation of requirements for CDK8 in cancers *in vivo* has been lacking. Conditional knockout of *Cdk8* in the intestinal epithelium using the constitutive Villin-Cre driver (expressed from embryonic day 12.5 onwards) did not hinder intestinal tumour development in *Apc* mutant mice; rather, it appeared to enhance tumourigenesis, and knockouts were reported to have lost the Polycomb group 2-mediated repressive histone mark, H3 lysine-27 trimethylation, thus upregulating oncogenic transcription (McClelland *et al*, 2015). However, differentiation in the intestinal epithelium of *Cdk8* knockouts was not assessed nor were effects on gene expression *in vivo*, and the role of CDK8 in restraining intestinal carcinogenesis has not been confirmed using inducible Cre drivers nor other means of tumour induction.

In contrast to CDK8, almost nothing is known about CDK19 roles in cancer, and whether it compensates for loss of CDK8 remains unknown. *In vitro* inhibition or knockdown experiments have suggested that CDK8 and CDK19 control different sets of target genes (Tsutsui *et al*, 2008; Galbraith *et al*, 2013; Poss *et al*, 2016). By genetic ablation in mice liver cells, we recently found that CDK8 and CDK19 are both required for hepatic carcinogenesis, and highlighted genetic interaction with p53 as critical for their roles in tumourigenesis (preprint: Bacevic *et al*, 2019).

A number of potent CDK8 pharmacological inhibitors have been developed (Porter *et al*, 2012; Dale *et al*, 2015; Pelish *et al*, 2015; Bergeron *et al*, 2016; Koehler *et al*, 2016; Schiemann *et al*, 2016; Hofmann *et al*, 2020), which are expected to also target CDK19. Anti-cancer activity of CDK8/19 inhibitors has been somewhat limited, and it has been reported that there may be only a small therapeutic window, due to systemic toxicity. However, debate about whether CDK8/19 inhibitor toxicity is on-target, *i.e.* due to inhibition of CDK8 and CDK19 (Clarke *et al*, 2016), or off-target, due to inhibition of other kinases (Chen *et al*, 2019), continues. Furthermore, at least some CDK8/19-mediated phenotypes appear to be kinase-independent (Audetat *et al*, 2017; Menzl *et al*, 2019; Steinparzer *et al*, 2019).

Thus, despite their established roles as regulators of Mediator, and considerable interest in their therapeutic targeting in cancer, we do not yet fully understand the redundant and specific roles of CDK8 and CDK19. We therefore used gene targeting in mice to address these questions, and determine whether their combined deletion is lethal. We confirm that knockout of the *Cdk8* gene in the intestinal epithelium has little or no effect on cell proliferation or differentiation. Furthermore, double deletion of *Cdk8* and *Cdk19* is compatible with cell proliferation in intestinal organoids. However, the double knockout reveals redundant functions in long-term control of cell proliferation and gene expression programmes. We uncover an unexpected requirement for these kinases in control of the CFTR pathway, a key player in cystic fibrosis, and show that CDK8/19 inhibition can modulate mucus production in organoids and *in vivo*.

Results

To evaluate the possible requirements for CDK8 for cell proliferation and survival in adult vertebrates, we designed and generated a conditional knockout allele of *Cdk8* in the mouse by Lox/Cre targeting exon 2 (Fig EV1A and B). This removes the critical catalytic lysine-52 and results in a frameshift that truncates over 90% of the protein. A similar conditional *Cdk8* allele was independently generated (McCleland *et al*, 2015). We studied the requirement for CDK8 in the adult mouse intestine since this is one of the most highly proliferative tissues in adults. We crossed *Cdk8*$^{lox/lox}$ mice to mice expressing a tamoxifen-inducible Cre under the control of the Villin promoter (el Marjou *et al*, 2004), and verified efficient deletion of *Cdk8* in the intestinal epithelium by genotyping and Western

 © 2022 The Authors

blotting (Figs EV1C and EV2A). The deletion was maintained after 2 months, showing that there is no counter-selection for non-recombined intestinal crypts (Fig EV2A). Mice lacking CDK8 were healthy and did not present any phenotypes in the intestine; there was no difference in the number of proliferating cells nor in cell cycle distribution as assessed by the number of BrdU positive cells after a two-hour pulse (Figs 1A and B, and EV2B). The number of stem cells, goblet cells, tuft cells, and Paneth cells was similar to wild-type mice, indicating that differentiation programmes were not affected (Figs 1B and EV2B). We performed RNA sequencing from the intestinal epithelium of wild-type and knockout mice, but, due to biological variability between animals and the limited number of replicates, could identify only 31 genes with statistically significant expression alterations (Table EV1). This nevertheless suggests that effects of CDK8 loss on gene expression may be somewhat restricted.

CDK8, like CDK7, can phosphorylate Pol II CTD (Rickert *et al*, 1999). Since cyclin C deletion in mice abolishes CDK8 activity yet does not affect Pol II CTD phosphorylation (Li *et al*, 2014), while CTD S5 phosphorylation is normal in a non-proliferative tissue (liver) of *Cdk7* knockout mice (Ganuza *et al*, 2012), we asked whether CDK7 and CDK8 can compensate for each other. We crossed single floxed *Cdk7* and *Cdk8* mice to generate $Cdk7^{lox/lox}$; $Cdk8^{lox/lox}$ mice, with Cre-expressed under control of a ubiquitous promoter (*Rpb1*). While complete CDK8 loss occurred in both intestine and liver, CDK7 loss was incomplete in both tissues (Fig EV3A and B). In the intestine, this incomplete deletion was expected since CDK7 is required for cell proliferation; thus, rare non-recombined crypts repopulate the epithelium (Ganuza *et al*, 2012). The strong reduction of CDK7 combined with ablation of CDK8 only led to a slight reduction in phosphorylation of Pol II CTD S5, while S2 and S7 phosphorylation were normal (Fig EV3A and B). These results do not indicate a critical role for CDK8 in this phosphorylation *in vivo*, and show that a low level of CDK7 is sufficient for Pol II CTD-phosphorylation. Furthermore, they highlight the difficulty of studying essential processes by combined gene deletions *in vivo*, since mosaicism provides a deletion bypass mechanism.

CDK8 was described as an oncogene in colorectal cancer where it promotes beta-catenin-dependent transcription (Firestein *et al*, 2008; Morris *et al*, 2008), suggesting that its deletion should inhibit intestinal tumorigenesis. Yet one study reported that constitutive *Cdk8* deletion in the intestine rather promotes tumourigenesis induced by Apc^{min} mutation (McCleland *et al*, 2015). To further investigate roles of CDK8 in intestinal tumourigenesis, we used a colitis-associated chemical model of intestinal tumourigenesis (azoxymethane-dextran sodium sulphate, AOM-DSS), combined with acute deletion of *Cdk8*. We thus treated adult control mice or mice lacking *Cdk8* with AOM-DSS, and sacrificed them 2 months after the first DSS treatment (Appendix Fig S1A). We observed no effect of *Cdk8* deletion on colitis-induced weight loss (Appendix Fig S1B) and no counter-selection for *Cdk8* deletion in the tumours (Appendix Fig S1C and D). There was no difference in number or area of tumours between WT and knockout animals (Fig 1C–E). We next tested whether CDK8 loss affects acute activation of the Wnt pathway, by concomitant homozygous deletion of the *Adenomatous polyposis coli* (*Apc*) tumour suppressor gene. Both $Apc^{-/-}$; $Cdk8^{+/+}$ and $Apc^{-/-}$; $Cdk8^{-/-}$ mice showed rapid morbidity necessitating sacrifice 5 days after tamoxifen treatment, and hyperplastic intestinal epithelium. There was no difference in number of Ki-67-positive cells in the intestinal epithelium lacking both *Cdk8* and *Apc* when compared to mutant *Apc* alone, indicating similar cell proliferation (Fig EV4A–D). Taken together, our data do not support a major role for CDK8 in controlling intestinal tumourigenesis in the mouse.

The lack of striking phenotypes of *Cdk8* deletion in the adult intestine, along with the almost complete conservation of the kinase domain between CDK8 and CDK19 and across vertebrate species (Appendix Fig S2A), suggested that if CDK8 has essential functions, CDK19 might be able to compensate for its loss. We therefore sought to generate a conditional double knockout. Since CDK8 and CDK19 are the only two kinases known to regulate the essential Mediator complex, we suspected that double *Cdk8/Cdk19* knockout in adult mice might be lethal. We therefore undertook a conditional deletion using intestinal organoids, which recapitulate many features of intestinal development, morphology and physiology while facilitating genetic manipulation *in vitro* (Clevers, 2016). We thus generated intestinal organoids from WT and $Cdk8^{lox/lox}$; *Vill::*$Cre^{ERT2/+}$ mice and disrupted *Cdk19* by CRISPR-Cas9-directed gene targeting (Appendix Fig S2B), using retroviral transduction of Cas9 and a synthetic guide RNA-expressing plasmid. *Cdk8* removal was efficient after 7 days of OH-tamoxifen treatment (Fig 2A and B). Cyclin C was lost specifically in double knockout organoids (Fig 2B) and this correlated with a loss of STAT1 phosphorylated on S727, a previously described CDK8 substrate (Bancerek *et al*, 2013), confirming redundancy of the two kinases. Growth appeared somewhat slower only in double knockout organoids (Fig 2C and D), with a corresponding larger fraction of non-proliferating cells, as demonstrated by loss of Ki-67 staining (Fig 2E and F). Consistent with slower growth, *Cdk8* deletion was counter-selected in the population of organoids with additional *Cdk19* knockout, as long-term culture resulted in reappearance of CDK8 in two out of three double knockout organoid populations, presumably due to expansion of a minor unrecombined population (Appendix Fig S3). Like double knockout of *Cdk8* and *Cdk19*, pharmacological inhibition of both CDK8 and CDK19 using Senexin B (Porter *et al*, 2012) also slowed, but did not abolish, growth of organoids (Fig 2G).

To determine effects of loss of the Mediator kinases on gene expression, we performed RNA-sequencing analysis of stable populations of single and double *Cdk8/Cdk19* knockout organoids. We performed spike-ins with ERCC RNA standards in proportion to genomic DNA content of each sample in case loss of Mediator kinases had global effects on total RNA or mRNA levels. This was not the case since changes were comparable whether samples were library normalised or spike-in normalised (Fig EV5A). CDK8 loss had a stronger effect (716 genes upregulated, 575 downregulated) than CDK19 loss (158 up, 151 down), while double knockout organoids (1,819 up, 1,363 down; Fig 3A and B; Dataset EV1) revealed functional redundancy between CDK8 and CDK19 in regulating gene expression. However, most expression alterations were minor, with only 830 genes deregulated by a factor of two or more. This is consistent with previous studies, none of which have shown sweeping changes in the transcriptome upon downregulation or inhibition of CDK8 or CDK19, but rather, alteration of a limited number of specific gene sets, including super-enhancer associated genes (Galbraith *et al*, 2013; Pelish *et al*, 2015; Clarke *et al*, 2016; Poss *et al*, 2016; El Khattabi *et al*, 2019; Steinparzer *et al*, 2019). In terms of genes controlling cell proliferation, cyclin A (*Ccna2*), cyclin B (*Ccnb2*) and

© 2022 The Authors

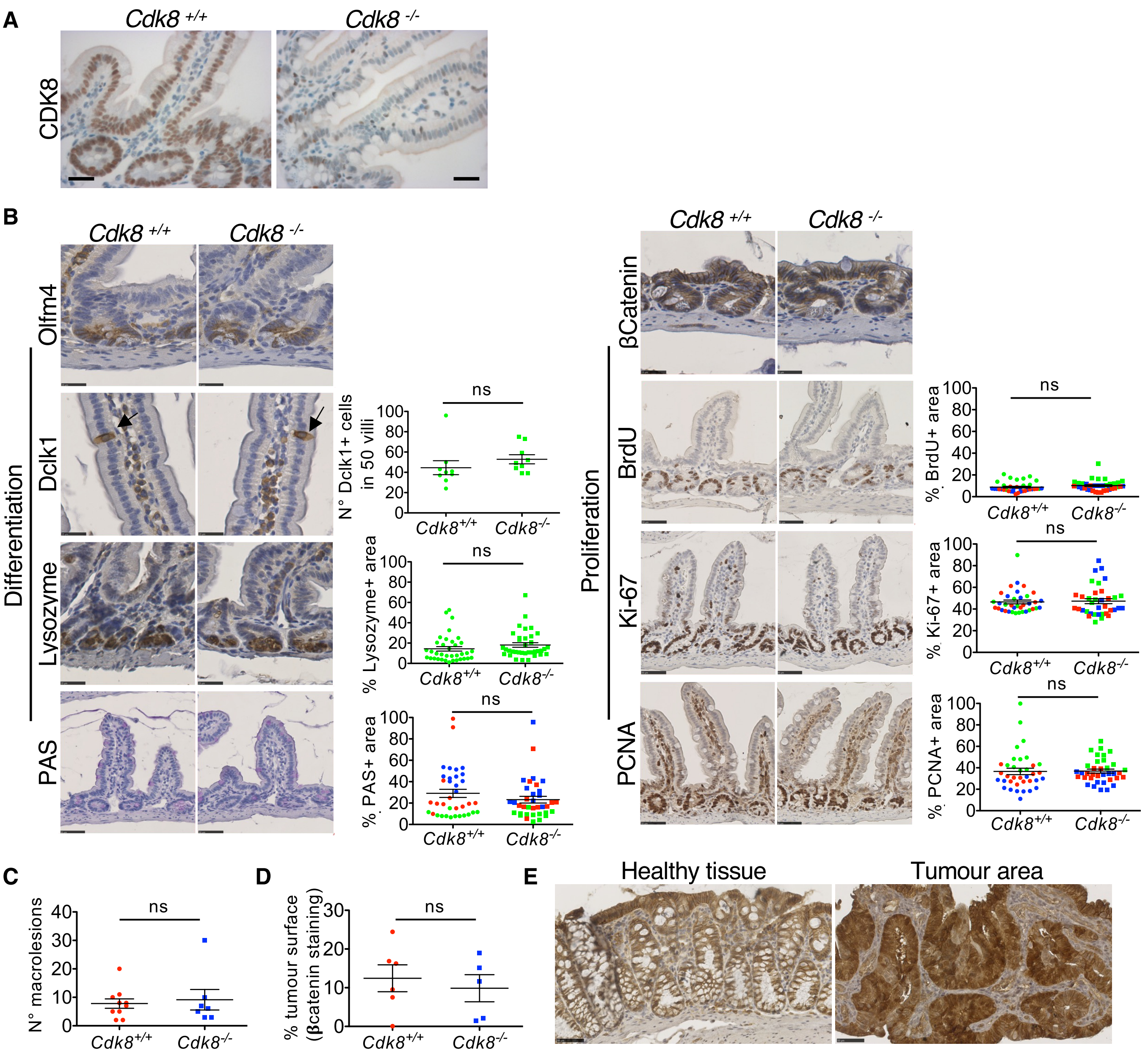

Figure 1. CDK8 knockout does not affect adult mouse intestine homeostasis nor chemically-induced carcinogenesis.

A Immunohistochemical staining of CDK8 in mouse small intestines collected two months after tamoxifen treatment. CDK8$^{+/+}$ depict mice with floxed Cdk8 alleles. Ten mice were used for the experiment (five males and five females). Scale bar, 25 μm.

B Analysis of cell differentiation (left) and proliferation (right) in the intestine after CDK8 deletion as in (A). Olfm4, Lysozyme, PAS, and Dclk1 staining were used to reveal, respectively, stem, Paneth, goblet, and tuft cells. β-Catenin staining allows detection of cancer cells (cytoplasmic vs nuclear localisation). Cell proliferation was assessed by PCNA, Ki-67, and BrdU (2 h pulse) staining. Scatter plots represent the percentage of the area stained by each antibody (relative to the area occupied by haematoxylin). For Paneth cells, BrdU, PCNA, and Ki67, only crypts were analysed. For goblet cells, crypts and villi were analysed. For Tuft cell quantification, Dclk1-positive cells were counted in 50 villi. Colour code depicts small intestine (green), proximal colon (blue), and distant colon (red). Mean ± SEM is shown. *P*-value of unpaired two-tailed *t*-test is indicated (ns, not significant; $P > 0.05$). Scale bars, 25 μm (Olfm4, Lysozyme, Dclk1, and β-Catenin) and 50 μm (PAS, BrdU, Ki-67 and PCNA; $n = 9$ biological replicates).

C Analysis of mouse colon after AOM/DSS treatment. Quantification of the number of neoplastic lesions ($n = 10$ for *Cdk8*$^{+/+}$, and $n = 7$ for *Cdk8*$^{-/-}$ mice). *P*-value of unpaired *t*-test is indicated: ns, not significant ($P > 0.05$). Mean ± SD is shown.

D Quantification of the percentage of the colon surface occupied by tumours. Intestine samples were stained for β-Catenin, and tumour regions with nuclear β-Catenin localisation were quantified using NDP.view software. Two-tailed *P*-value of unpaired *t*-test is indicated; ns, not significant ($P > 0.05$). Mean ± SD is shown ($n = 6$ biological replicates).

E Example of IHC with β-Catenin staining of tumour-free regions with membrane β-catenin localisation (left) and tumour regions with nuclear β-catenin localisation (right). Scale bars, 50 μm.

 © 2022 The Authors

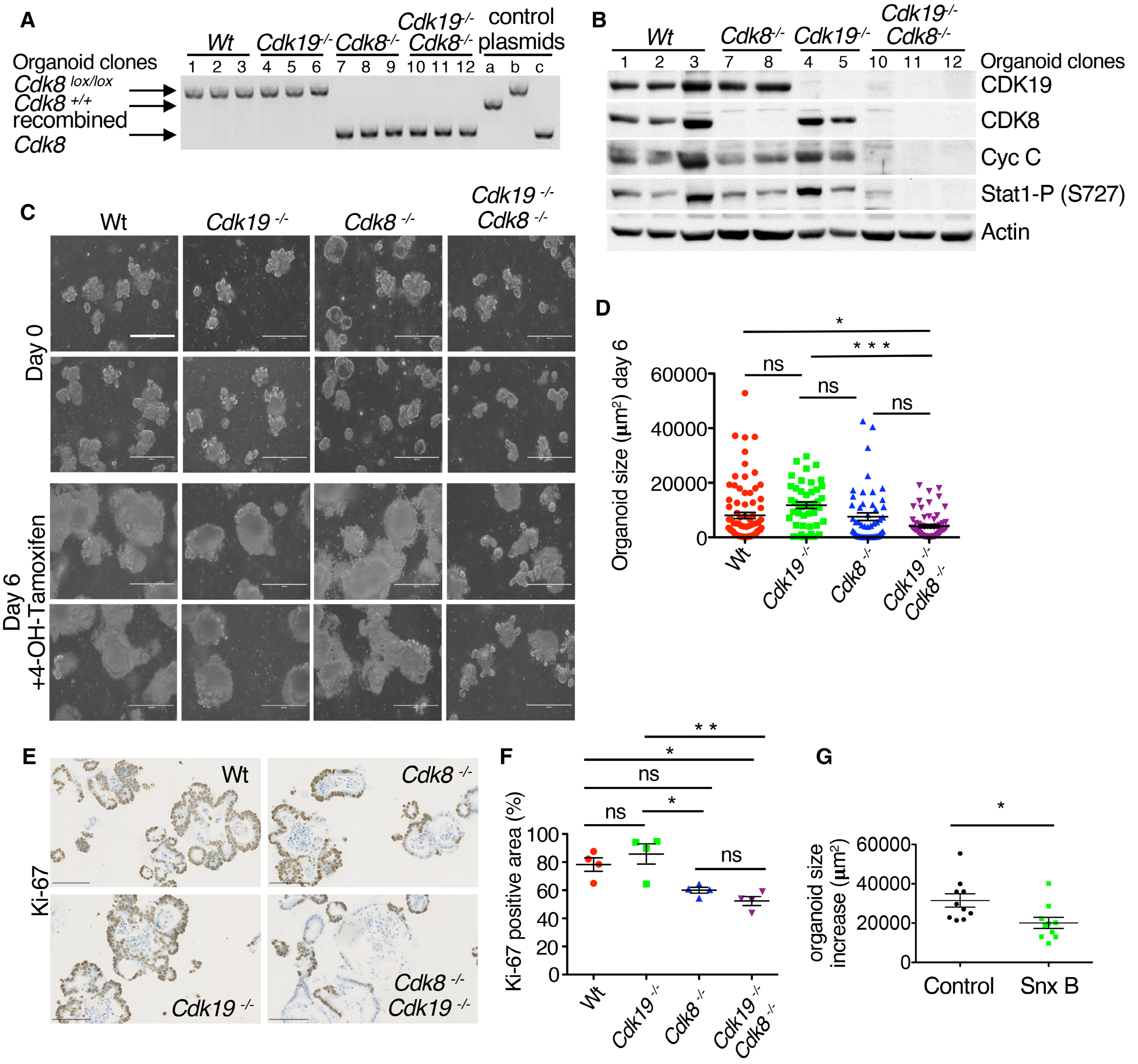

Figure 2. Double CDK8/CDK19 knockout intestinal organoids show decreased cell proliferation.

A Genotyping confirms the loss of *Cdk8* exon 2 in *Cdk8*$^{-/-}$ and *Cdk8*$^{-/-}$/*Cdk19*$^{-/-}$ organoids after 7 days of OH-tamoxifen treatment. Control plasmids (a, b, and c) are described in Fig EV1C.

B WB of organoid samples after 7 days of OH-tamoxifen treatment; β-actin was used as loading control.

C Phase contrast images of organoids before and after 6 days of OH-tamoxifen treatment. Scale bars, 150 μm.

D Quantification of organoid size at day 6, shown in (C; mean ± SEM are shown). *P*-value, one-way ANOVA (Bonferroni test): (*) $P \leq 0.05$, (***) $P \leq 0.001$; ns, not significant ($P > 0.05$; $n = 47$ biological replicates).

E IHC staining of organoids (day 7 of OH-tamoxifen treatment) with Ki-67 antibody. Scale bars, 100 μm.

F Quantification of Ki-67 positive area (% of the total area of the organoids; mean ± SEM) in the four different genotypes presented in (E). Areas with positive Ki-67 signal were detected and quantified using QuPath and ImageJ programs. Four technical replicates from two biological replicates of each genotype were analysed. Adjusted *P*-values of ordinary one-way ANOVA (Bonferroni test) are indicated: (**) *P*-values ≤0.01; (*) *P*-values ≤0.05; ns, not significant ($P > 0.05$).

G Quantification of the increase in size over 9 days of control and Senexin B-treated (10 μM) organoids ($n = 10$ biological replicates, mean ± SEM are shown). *P*-value of unpaired two-tailed *t*-test is indicated: (*) $P \leq 0.05$.

© 2022 The Authors

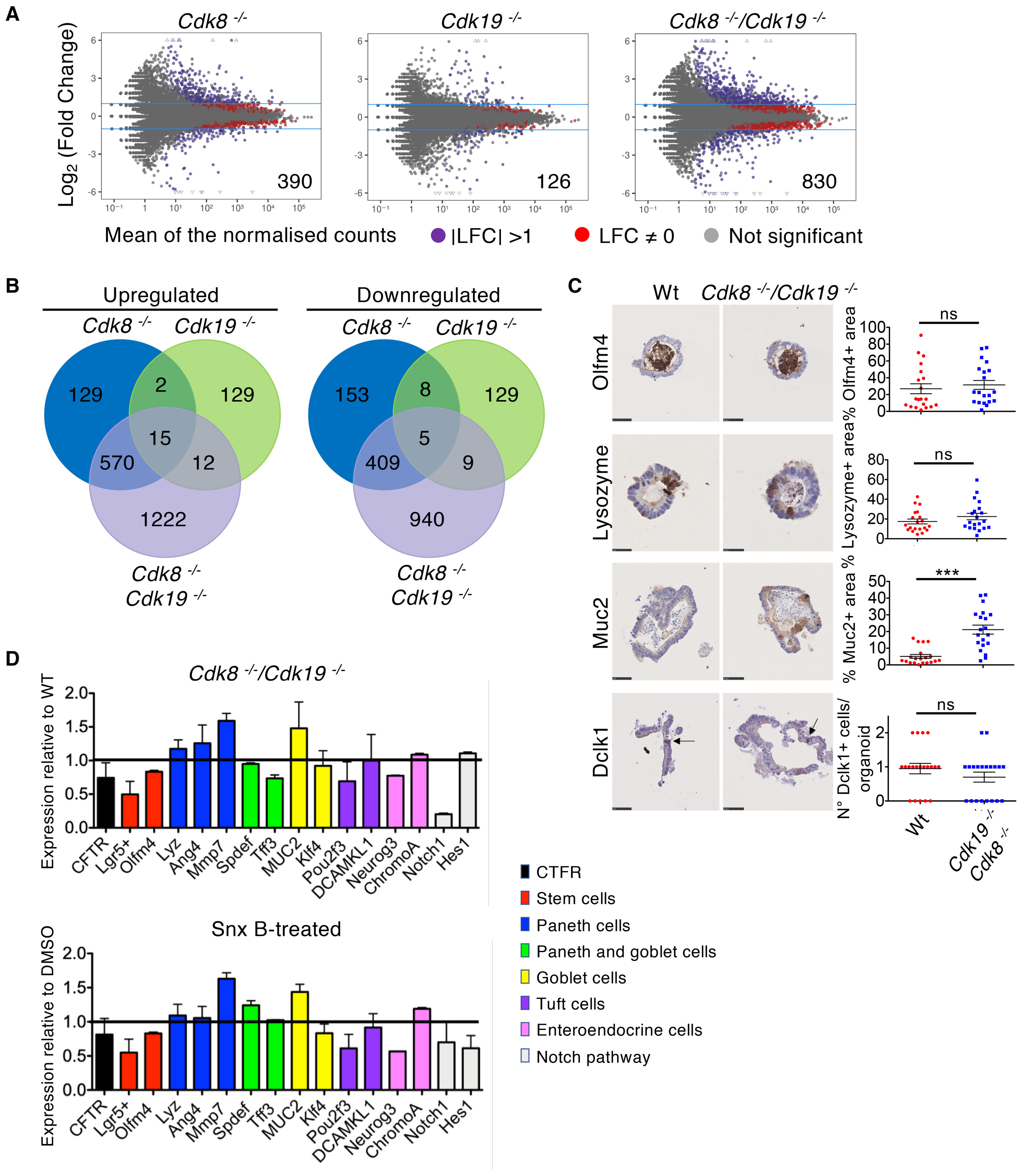

Figure 3.

cyclin E (*Ccne2*) were slightly downregulated in double knockouts, but this is likely to be a consequence rather than a cause of reduced cell proliferation. CDK8 has previously been found to regulate the p53 and c-Myc pathways (Donner *et al*, 2007; Adler *et al*, 2012), but intestinal cells lacking both kinases showed only a slight (though significant) downregulation of c-Myc, while p53 was not affected. In

© 2022 The Authors

Figure 3. CDK8 and CDK19 show functional redundancy in regulation of gene expression, while their knockout does not perturb differentiation in intestinal organoids.

A Dot plot analysis of differentially expressed genes (DEGs). Red dots: DEGs with *P*-value ≤ 0.05; purple dots: $\log_2$ fold change (LFC) > 1 or < −1, *P*-value ≤ 0.05; grey dots: not significant. Numbers inside plots indicate the number of genes deregulated more than 2-fold. Three biological replicates of each genotype were used for this analysis.

B Venn diagrams indicating intersection of genes with altered expression in the indicated genotypes.

C Left, IHC staining of WT and *Cdk8*$^{-/-}$/*Cdk19*$^{-/-}$ organoids with Olfm4 (stem cells), Lysozyme (Paneth cells), Muc2 (goblet cells), and Dclk1 (tuft cells) antibodies. Scale bars, 50 μm (Olfm4, Muc2, Dclk1) and 25 μm (Lysozyme). Right, quantification of Olfm4, Lysozyme and Muc2 positive area (% of the total organoid area as indicated by haematoxylin staining; mean ± SEM), or the number of Dclk1 positive cells per organoid. *P*-value, unpaired two-tailed *t*-test: (***) *P*-value ≤ 0.001; ns, not significant (*P* > 0.05). Twenty different organoids from two biological replicates of each genotype were used for the analysis.

D qRT–PCR analysis of indicated mRNA levels in Wt and *Cdk8*$^{-/-}$/*Cdk19*$^{-/-}$ (top) or Senexin B-treated (10 μM, for 24 h; bottom) organoids, relative to WT or vehicle (DMSO)-treated organoids, respectively, showing mean ± SEM. Three technical replicates were used for this analysis.

contrast, cyclin G1, a positive mediator of p53 responses and RB functions with a role in cell cycle arrest (Zhao *et al*, 2003), and p21 (*Cdkn1A*), a p53 target that inhibits cyclin-dependent kinases to induce cell cycle arrest, were more strongly upregulated in the double knockout organoids. Interestingly, while most genes significantly deregulated in single *Cdk8* knockouts were also deregulated in double knockout, 282 out of 1,291 (22%) were only identified as deregulated in the single *Cdk8* knockout. This was not due to bias in variance or low read counts (Fig EV5B and C). Further analysis showed that in double knockouts, the read counts for these genes always showed the same trend as in single *Cdk8* knockouts and they were approximately halfway between the single *Cdk8* knockout and WT or *Cdk19* knockout organoids (Fig EV5D and E). In contrast, most genes (258 of 309, 83%) identified as deregulated only in *Cdk19* knockouts were indeed not deregulated in *Cdk8* knockouts or double knockouts. Furthermore, of these genes, double knockouts generally had similar expression levels to WT or *Cdk8* knockout (Fig EV5D and E). Taken together, we conclude that CDK8 and CDK19 co-regulate the majority of their target genes, but a subset of genes appears to be controlled in an opposite manner by these kinases, with CDK8 being dominant over CDK19.

We assessed whether the changes in gene expression upon combined loss of CDK8 and CDK19 might be accompanied by changes in differentiation. We first performed IHC analyses on particular marker genes for different cell types in WT and double *Cdk8/19* knockout organoids. Muc2 staining (goblet cells) was strikingly elevated in knockouts, while staining for proteins specific for stem cells (the luminally secreted Olfm4), Paneth cells (lysozyme), and tuft cells (Dlck1) was not altered (Fig 3C), indicating that differentiation into all cell types analysed occurs. We next correlated global gene expression changes of double-knockout organoids with mouse single cell RNA-seq data that identify different intestinal cell types (Haber *et al*, 2017). Most of the signature genes for each cell type were not significantly deregulated in organoids lacking CDK8 and CDK19 (Appendix Fig S4), also suggesting that differentiation is robust in the absence of Mediator kinases. Nevertheless, a subset (227 out of 459) of enterocyte-signature genes were significantly affected, most of which (219 out of 227) were upregulated, while a smaller proportion (43 out of 122) of stem cell signature genes were identified, most of them being downregulated (32 of 43). To investigate this further, we quantified expression of genes characteristic for different intestinal cell-types by qRT–PCR on WT and double-knockout organoids, and organoids treated with Senexin B to inhibit both kinases. We also analysed Notch1 and one of its key targets, Hes1, since Notch signalling inhibition causes reduced stem cell proliferation and increased secretory cell type differentiation in the mouse intestine (VanDussen *et al*, 2012). This analysis showed that there was no systematic increase or decrease of all markers of any particular lineage, and no changes for any gene of more than about two-fold (with the exception of Notch1). There were, nevertheless, slight but reproducible changes, for example, a decrease in expression of the stem cell marker *Lgr5* and an increase of the goblet cell marker *Muc2* (Fig 3D). Importantly, these changes were similar between organoids deleted for *Cdk8/19* and WT organoids treated with the CDK8/19 inhibitor Senexin B (Fig 3D). Since these data were derived from total epithelial RNA, it is possible that larger changes might occur in specific cell sub-types. Taken together, our results show that differentiation into all cell types occurs in the absence of Mediator kinases, but do not rule out minor changes in differentiation or stem cell numbers.

Geneset Enrichment Analysis (GSEA) in double-knockout organoids unexpectedly revealed a striking alteration of genes also modulated in intestinal knockouts of the cystic fibrosis transmembrane conductance regulator, CFTR (Fig 4A), with 98% of intestinal genes deregulated in *Cftr* knockout also deregulated in *Cdk8/19* knockout. Most genes upregulated in the intestine in *Cftr* knockout (70% in the ileum and 86% in the duodenum) were similarly upregulated in *Cdk8/19* knockout, with 22–25% of downregulated genes similarly diminished (Fig 4B, Dataset EV2). Specifically, in *Cdk8/19* knockout, expression of genes involved in mucus production, *Muc2*, *Muc3*, *Muc13*, *Nlrp6*, *Agr2*, *Gcnt4*, *Tff1*, were upregulated, while *Cftr* was reduced. We validated changes of selected genes by qRT–PCR in organoids of all genotypes (Fig 4C). The loss of *Cftr* mRNA was more pronounced at the protein level, since CFTR protein was lost in double mutant organoids (Fig 4D). To see if similar changes occur upon inhibiting CDK8/19 kinase activity, we performed RNA–seq analysis of WT organoids compared with WT organoids treated with Senexin B for 16 h or 24 h. 1,505 genes were identified as deregulated after 16 h of CDK8/19 inhibition, 189 of which by a factor of two or more, compared to 4,268 genes after 24 h (486 changes more than two-fold; Fig 4E, Dataset EV3). Since longer Senexin B treatments might have indirect effects, we performed GSEA on genes with altered expression in 16-h samples. This showed a strong overlap with genes deregulated in knockout of *Cftr* as well as with genes downregulated in the human colorectal cancer cell line HCT-116 upon treatment with a different CDK8/19 inhibitor, cortistatin A (Fig 4F).

Cystic fibrosis is a disease of mucosal epithelia which also affects the intestine, and is characterised by excessive mucus accumulation and frequent inflammation (Ehre *et al*, 2014). We thus wanted to

© 2022 The Authors

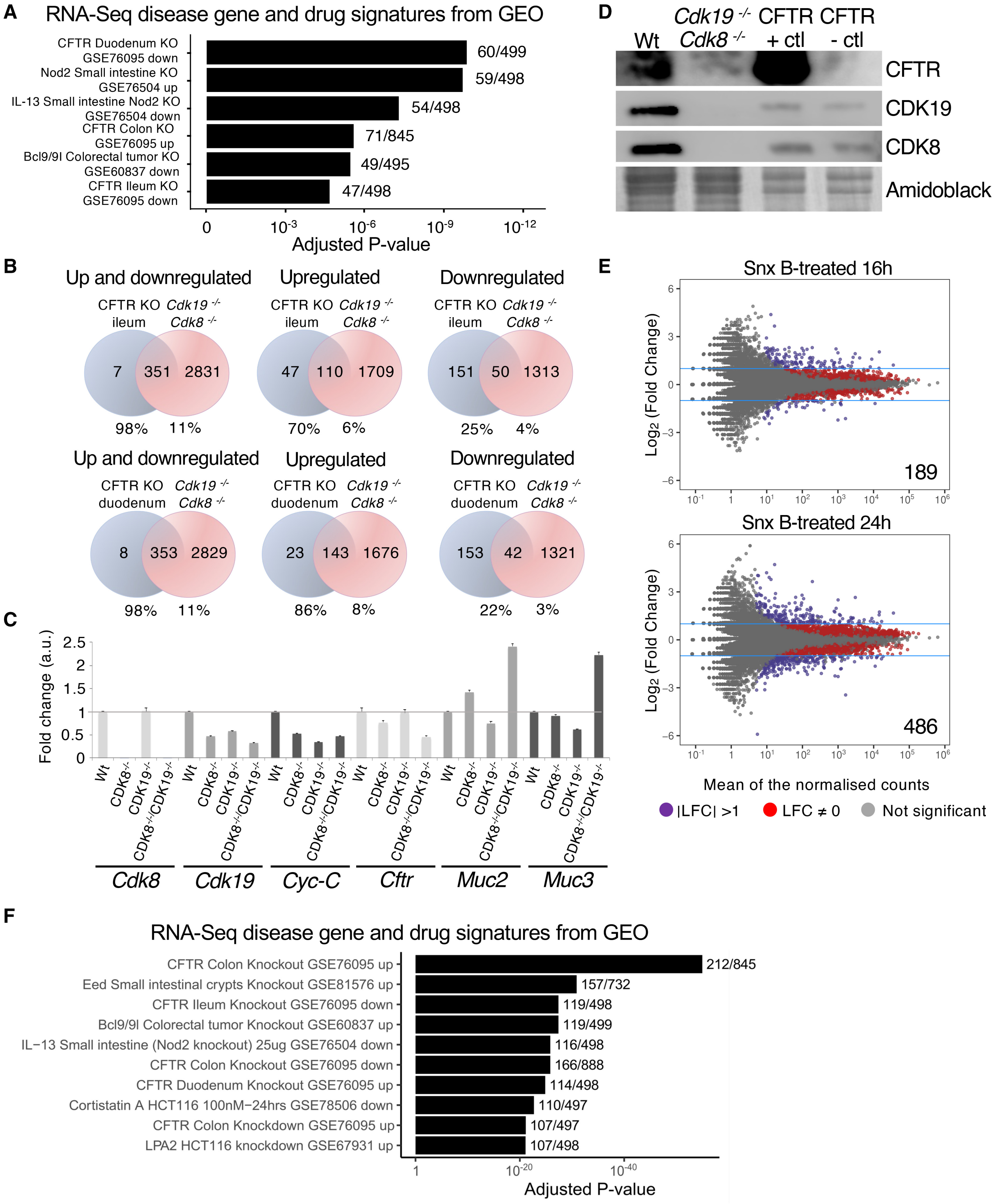

Figure 4.

© 2022 The Authors

Figure 4. CDK8 and CDK19 regulate the CFTR pathway in the small intestine.

A Gene set enrichment analysis (using Enrichr database) of highly deregulated genes in *Cdk8*$^{-/-}$/*Cdk19*$^{-/-}$ organoids. Manually curated signatures extracted from RNA-seq studies in GEO where gene expression was measured before and after drug treatment, gene perturbation or disease.

B Venn diagrams indicating intersection of genes with altered expression in the *Cftr* mouse knockout small intestine (ileum, top, and duodenum, bottom) and *Cdk8*$^{-/-}$/*Cdk19*$^{-/-}$ organoids. Numbers below give the percentage of each gene set in the intersection.

C qRT–PCR analysis of indicated mRNA levels in Wt, *Cdk8*$^{-/-}$, *Cdk19*$^{-/-}$ and *Cdk8*$^{-/-}$/*Cdk19*$^{-/-}$ organoids, showing mean ± SEM. Three technical replicates were used for the analysis.

D WB of indicated proteins extracted from Wt and *Cdk8*$^{-/-}$/*Cdk19*$^{-/-}$ organoids. Included are CFTR positive and negative control cell line samples obtained from the Cystic Fibrosis Foundation (CFF). Amidoblack was used as loading control.

E Dot plot analysis of differentially expressed genes (DEGs) in Senexin B (Snx B)-treated (10 μM; for 16 h, top, and 24 h, bottom) organoids. Red dots: DEGs with *P*-value ≤ 0.05; purple dots: $\log_2$ fold change (LFC) > 1 or < −1, *P*-value ≤ 0.05; grey dots: not significant, NS. Numbers inside plots indicate the number of genes deregulated more than 2-fold.

F Gene set enrichment analysis (using Enrichr database) of highly deregulated genes in Senexin B-treated (16 h) organoids. The signatures were extracted from RNA-seq studies in GEO where gene expression was measured before and after drug treatment, gene perturbation or disease, and an additional step of manual curation was performed to select those related to the intestine.

see whether the transcriptome alterations in mutant intestinal organoids translate into a cystic fibrosis (CF)-like phenotype. Staining mucin polysaccharides by periodic acid-Schiff (PAS) showed intense mucus accumulation in goblet cells specifically in *Cdk8*$^{-/-}$ *Cdk19*$^{-/-}$ organoids (Fig 5A and B). Time-lapse video microscopy confirmed accelerated mucus release from double-mutant organoids (Fig 5C and D). Functionality of CFTR can be tested in intestinal organoids using forskolin, an adenylate cyclase activator that, if CFTR is functional, induces luminal fluid secretion and organoid swelling (Dekkers *et al*, 2013). We found that forskolin-induced swelling (FIS) occurs in wild-type but not double-mutant organoids (Fig 5E and F; Movie EV1), indicating that CFTR downregulation upon loss of CDK8 and CDK19 is accompanied by a *Cftr* mutant phenotype.

These results from genetically modified organoids implicate CDK8 and CDK19 as functionally redundant regulators of the CFTR pathway in the small intestine. Since similar transcriptional changes occur upon CDK8/19 inhibition, we investigated whether Senexin B treatment would also affect forskolin-induced swelling. We thus treated wild-type organoids with Senexin B, for 1 h or 24 h before adding forskolin. We reasoned that if effects of CDK8/19 inhibition depend on transcriptional changes, they might take 24 h to become detectable, whereas if they depend only on post-transcriptional regulation, they might be seen after 1 h. Figure 5F shows that there is a dose-dependent reduction of swelling after 24 h, but not 1 h, of Senexin B treatment, indicating that loss of CDK8/19 kinase activity at least partly recapitulates a *Cftr*-mutant phenotype. To see whether this also correlates with downregulation of CFTR expression, we performed qRT–PCR analysis on organoids treated with Senexin B over a time course. We found that Senexin B treatment for 24 h led to the downregulation of *Cftr* and upregulation of *Muc3* expression (Fig 5G) as seen upon genetic ablation of both *Cdk8* and *Cdk19*, suggesting that these changes are indeed dependent on CDK8/19 kinase activity. Whether or not the loss of FIS can be directly attributed to loss of CFTR expression itself is uncertain. FIS was visibly less inhibited by 24 h Senexin B treatment than by genetic knockout of *Cdk8/19*, which is consistent with only a minor drop of CFTR protein expression upon Senexin B treatment (Fig 5H).

Finally, we tested whether CDK8/19 inhibition *in vivo* would recapitulate CF-like phenotypes seen in intestinal organoids. We fed WT mice for 4 or 11 days with a new potent CDK8/9 inhibitor, SNX-631, which has been validated *in vivo* (Ding *et al*, 2022), or vehicle. The drug was well tolerated and mice did not lose weight during the experiment (Appendix Fig S5A). We did not observe any severe CF-like pathological manifestations such as intestinal occlusion or shortening of the colon (Appendix Fig S5B). Surprisingly, there was a strong upregulation of both CDK8 and CDK19 protein expression in the intestinal epithelium of mice treated with the inhibitor, suggesting feedback regulation to compensate for the kinase inhibition, and providing a potential biomarker for CDK8/19 inhibition *in vivo* (Fig 6A). While CFTR protein levels were lower than in organoids and could not be detected by immunoblotting in control mice, CFTR was also upregulated upon CDK8/19 inhibition,

Figure 5. Knockout or inhibition of CKD8/19 leads to *Cftr* mutant-like phenotype.

A Histological PAS staining of organoids treated for 7 days with OH-tamoxifen. Scale bar, 50 μm.

B Quantification of PAS signal (% of total organoid area; mean ± SD are shown) in the four different genotypes presented in (A). Areas containing positive PAS staining were detected and quantified using QuPath and ImageJ programs. Adjusted *P*-values of ordinary one-way ANOVA followed by Tukey's multiple comparison test are indicated: (***) *P*-value ≤ 0.001; ns: not significant ($P > 0.05$). Four technical replicates from two biological replicates of each genotype were analysed.

C Representative phase contrast images of organoids at the indicated time points after 7 days of OH-tamoxifen treatment are shown. Scale bar, 100 μm.

D Quantification of the time needed for mucus release (observed as a dark staining in the centre of the organoid; mean ± SD are shown). Adjusted *P*-values of ordinary one-way ANOVA followed by Tukey's multiple comparison test are indicated: (***) *P*-value ≤ 0.001; ns: not significant ($P > 0.05$); ($n = 17$ for Wt, 11 for *Cdk8*$^{-/-}$, 18 for *Cdk19*$^{-/-}$, and 12 for *Cdk8*$^{-/-}$/*Cdk19*$^{-/-}$, all biological replicates).

E Fluorescence confocal microscopy images of Calcein green–labelled WT and Cdk8$^{-/-}$/Cdk19$^{-/-}$ organoids treated with forskolin. Scale bars, 100 μm.

F Quantification of forskolin-induced swelling in WT organoids treated for 1 h or 24 h with 0.1, 1 or 10 μM Senexin B (SenB), as indicated, or double KO organoids; DMSO vehicle was used as control. The surface area of individual organoids at different time points relative to the area at $t = 0$ (100%) was measured (mean ± SD, $n = 8$ biological replicates). Linear regression lines are shown.

G qRT–PCR analysis of *Cftr* and *Muc3* mRNA levels in WT organoids either not treated ($t = 0$), or treated with 10 μM Senexin B for 2 or 24 h, showing mean ± SEM. Three technical replicates were used for the analysis.

H WB of indicated proteins extracted from Senexin B-treated (10 μM, 2 h and 24 h) organoids (left), and WT and *Cdk8*$^{-/-}$/*Cdk19*$^{-/-}$ organoids (right). Amidoblack was used as loading control.

© 2022 The Authors

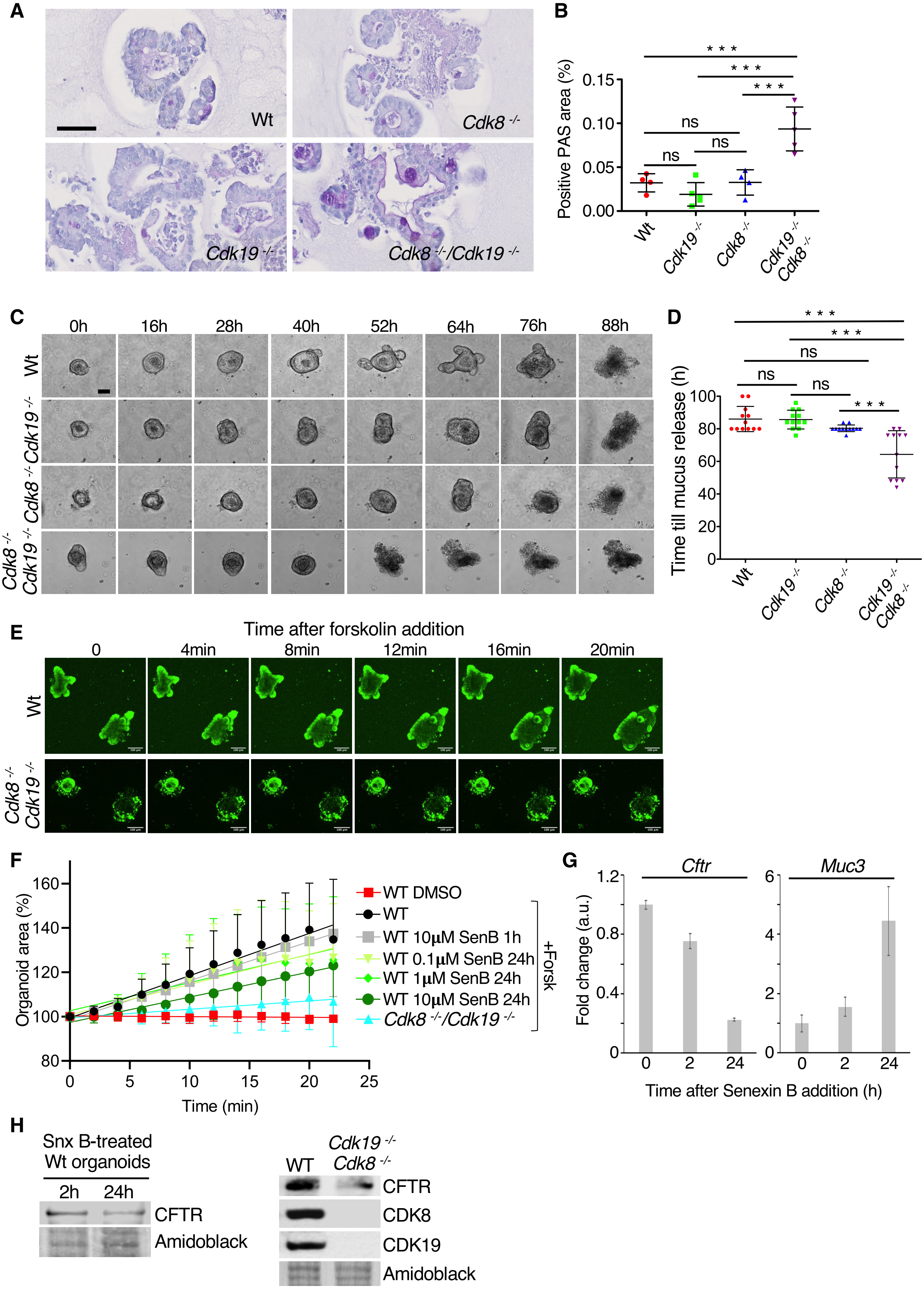

Figure 5.

© 2022 The Authors

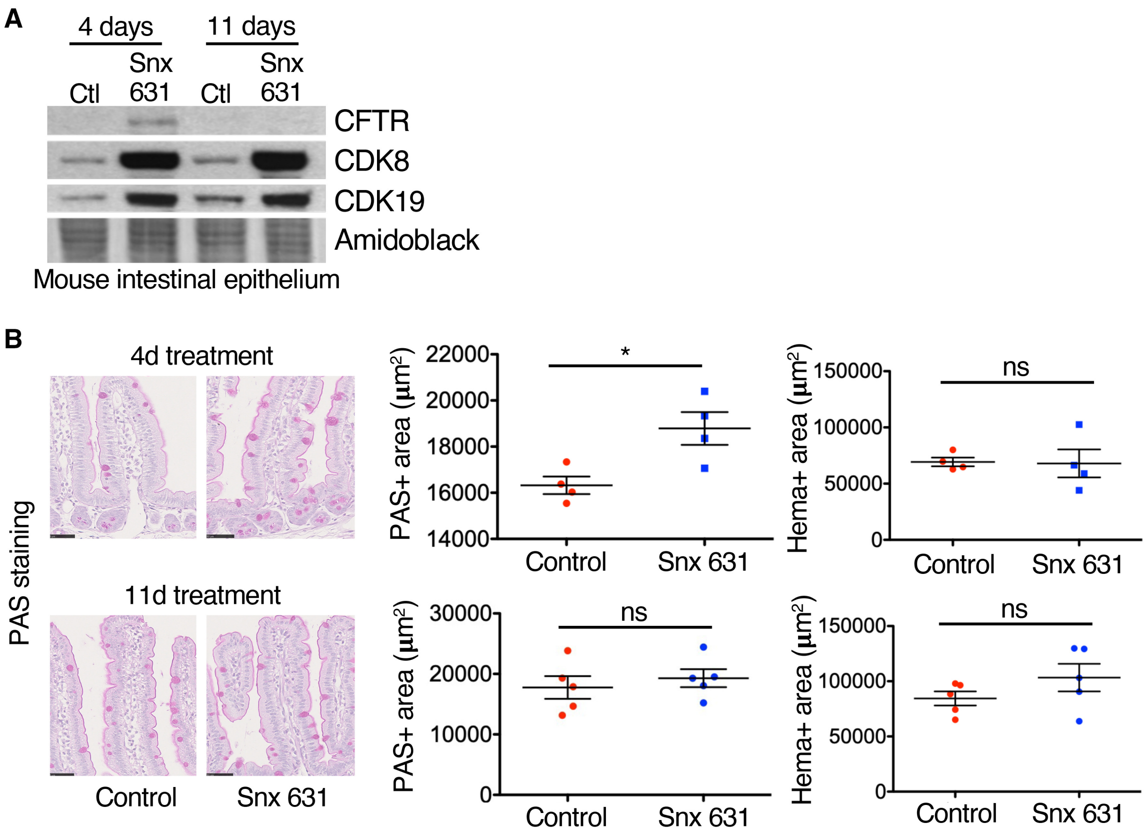

Figure 6. CDK8/19 inhibition in mice causes increase of goblet cells in the intestine and compensatory upregulation of both kinases.

A WB of indicated proteins extracted from intestinal epithelium of control mice or mice treated with SNX-631 for 4 or 11 days.

B Left, immunohistochemical PAS and haematoxylin staining of mouse small intestines collected at 4 and 11 days of SNX-631 treatment; scale bars 50 μm. Right, quantification of PAS- and haematoxylin (Hema)-positive area (mean ± SEM), using QuPath and ImageJ software. Adjusted *P*-values of unpaired *t*-test are shown: (*) $P \leq 0.05$; ns, not significant ($P > 0.05$). Eight mice (4 treated with SNX-631 and 4 control) were used in this quantification.

correlating with the increased CDK8/19 protein. This may also tend to counteract effects of the inhibitors on intestinal physiology. Nevertheless, after 4 days of SNX-631 treatment, there was a clear increase of mucin in the intestinal epithelium, recapitulating the most obvious phenotype of *Cdk8* and *Cdk19* deletion in organoids (Fig 6B). However, this was transient as differences had largely disappeared by 11 days, likely due to adaptation and the compensatory increase in CDK8/19 expression.

Discussion

This study shows that Mediator kinases are both functionally redundant and largely dispensable for cell survival, proliferation, and differentiation, but may be essential for regulation of specific gene sets in particular cell types; in this case, the CFTR pathway in the intestinal epithelium. Nevertheless, while cell proliferation defects have not previously been reported in HCT-116 cancer cells lacking both kinases (Koehler *et al*, 2016), our data suggest that in the intestinal epithelium, cells devoid of both CDK8 and CDK19 have an increased tendency to become quiescent, implying that CDK8 and CDK19 provide a growth advantage.

Our *in vivo* results do not support an oncogenic role for CDK8 in intestinal tumourigenesis, in contrast to early *in vitro* studies (Firestein *et al*, 2008; Morris *et al*, 2008). Another more recent study using the heterozygous germline Apc^{min} mutant model of intestinal tumourigenesis also concluded that *Cdk8* deletion does not hinder tumourigenesis; on the contrary, in this model, while there was no difference in micro-adenoma formation, detectable increases in tumour number, size, and fraction of proliferating cells were observed upon deletion of *Cdk8* (McCleland *et al*, 2015). The reasons for the slight difference in effects of *Cdk8* deletion between chemical carcinogenesis and Apc^{min} mutation are currently unclear, but, taken together, these studies suffice to conclude that *Cdk8* has neither oncogenic nor strong tumour suppressor activity in the mouse intestine.

Our results indicate that Mediator kinases are not essential for controlling the expression of all genes. In contrast to knockouts of essential Mediator subunits that caused an approximately 7-fold global downregulation of the entire transcriptome (El Khattabi *et al*, 2019), we found that knockout of Mediator kinases has much more modest effects. Slightly more genes were upregulated than downregulated upon loss of either CDK8 alone or both kinases, while effects of combined deletion were more than additive of

© 2022 The Authors

effects of single deletions, indicating functional redundancy. Organoid growth and differentiation were not prevented by knockout of both CDK8 and CDK19, suggesting that, generally, they are not essential for implementation of new transcriptional programmes. Interestingly, we found that there was a strong overlap between transcriptome changes of double-knockout organoids and intestinal knockout of the gene encoding CFTR, a chloride and bicarbonate ion-channel that regulates fluid homeostasis in epithelia, and whose mutation causes cystic fibrosis, a disease associated with mucus retention and inflammation of epithelia. Double-knockout organoids showed increased mucin expression and strong accumulation of mucins in goblet cells, coupled with a precocious secretion of mucus, as well as a lack of forskolin-induced swelling, which depends on CFTR (Dekkers *et al*, 2013), indicating that CDK8/19 regulate fluid and/or mucus homeostasis. This appears to depend on their kinase activity, as specific inhibition of both kinases for 24 h using Senexin B impaired forskolin-induced swelling in a dose-dependent manner, albeit less so than knockout of both kinases. Since acute CDK8/19 inhibition in organoids for 1 h prior to the forskolin assay had no effect, the loss of swelling upon CDK8/19 inhibition is likely to be a consequence of effects on transcription. While changes in FIS and in CFTR expression are consistent with CFTR being directly involved in the phenotype, we cannot at this stage conclude that CFTR is the sole player. Indeed, there are 95 solute carriers whose expression is deregulated in CDK8/19 knockouts (Table EV2), including four inorganic anion transporters of the Slc26 family, all of which are upregulated, and which might contribute to the phenotype.

While this study was still ongoing, a report was published in which, using a similar genetic approach in mice and intestinal organoids, CDK8 and CDK19 were found to be dispensable for intestinal morphology and viability but were observed to redundantly control intestinal lineage specification (Dannappel *et al*, 2022). In particular, a decrease in number of paneth cells, tuft cells, and goblet cells were reported in intestinal tissue lacking both kinases, a result that we do not reproduce. Consistent with previous publications highlighting tuft cells as a rare (and only relatively recently identified) population in the intestinal epithelium (Gerbe *et al*, 2009, 2011, 2016), we found on average one tuft cell per villus in WT or *Cdk8*$^{-/-}$ mice, rather than the average of 5 reported in control mice of the study (Dannappel *et al*, 2022). Similarly, when analysing the entire intestinal epithelium, we did not observe the large numbers of goblet cells presented in control tissue in the same study. Interestingly, secretory lineage hyperplasia occurs upon parasitic infections and is mediated by pro-inflammatory cytokines (Gerbe *et al*, 2016), while CDK8 and CDK19 potentiate NFκB-mediated pro-inflammatory transcription (Chen *et al*, 2017) and limit production of anti-inflammatory cytokines (Johannessen *et al*, 2017). Thus, it is plausible that dampened responses to the microbiome underlie the reported requirement for CDK8 and CDK19 in controlling the balance of secretory cells and other cell types in the intestinal epithelium (Dannappel *et al*, 2022). Further independent studies will undoubtedly resolve this issue. Nevertheless, both this report (Dannappel *et al*, 2022) and our results highlight the fact that cell proliferation and differentiation in a complex tissue can occur in the simultaneous absence of both Mediator kinases.

How CDK8 and CDK19 act mechanistically to control specific transcriptional pathways remains unknown. They may bind directly to promoters of target genes and potentiate effects of transcription factors on transcriptional elongation by inducing phosphorylating of RNA polymerase 2 CTD Ser2 (Galbraith *et al*, 2013; Chen *et al*, 2017), but the key question of how they are directed to specific genes has not yet been resolved, and further studies should address this gap in our molecular understanding.

Whether or not CDK8 or CDK19 are implicated in the pathogenesis of cystic fibrosis also remains an open question. Almost all CF patients harbour genetic mutations in the *CFTR* gene, yet identical mutations do not have identical disease severity, and the variability between patients is associated with different genetic loci (Wright *et al*, 2011; Corvol *et al*, 2015). There are also significant but variable gene expression alterations in *CFTR* mutant cells and upon therapeutic interventions, some of which may influence disease phenotypes (Hodos *et al*, 2020). The genes encoding CDK8, CDK19, and cyclin C have not so far been associated with CF. *CDK19* is downregulated upon several model therapeutic interventions, including overexpression of the micro-RNA miR-138, which promotes CFTR expression (Ramachandran *et al*, 2012; Hodos *et al*, 2020). However, in our study, CDK19 knockout alone was insufficient to cause a CF phenotype in intestinal organoids, suggesting that variation in CDK19 expression does not affect CFTR. Identifying the mechanisms by which CDK8 and CDK19 affect expression of genes in the CFTR pathway may improve understanding the pathophysiology of cystic fibrosis, but will require further studies. There is an intriguing connection between CFTR and cell proliferation in the intestine, as CFTR null mice have increased cell proliferation (Gallagher & Gottlieb, 2001). However, this is unlikely to be related to CDK8/19, whose inhibition or knockout rather causes a decrease in cell proliferation, at least in organoids.

Finally, our study shows that CDK8/19 inhibitors are well tolerated in mice and, at least in short term treatments, affect intestinal epithelium physiology by inducing increased mucin expression. We also showed that they trigger upregulation of CDK8 and CDK19 expression, which constitutes a promising biomarker for CDK8/19 inhibitor target engagement *in vivo*. CFTR inhibitors have been proposed to treat secretory diarrhoea (Ma *et al*, 2002), a major cause of infant mortality in developing countries as well as being a frequent complication of cancer drug treatments. We suggest that it will be important to test whether CDK8/19 inhibitors have therapeutic benefit in such settings.

Materials and Methods

Animal studies

All animal experiments were performed in accordance with international ethics standards and were subjected to approval by the Animal Experimentation Ethics Committee of Languedoc Roussillon. They were housed in an SPF animal facility according to international guidelines, with a maximum of five mice per cage (501 cm^2), sawdust and woodshavings, and unlimited access to food and water. ARRIVE guidelines for experimental design were followed.

Cdk8 conditional knockout mice

Cdk8$^{lox/lox}$ mice were generated as follows: An 8,076 bp genomic fragment (mouse chromosome 5: 146,254,503 to 146,262,579)

 © 2022 The Authors

enclosing the essential exon 2 (whose deletion results in loss of the essential catalytic lysine residue and causes a frameshift truncating over 90% of the protein) of the CDK8 gene was amplified by PCR from genomic DNA of 129/Sv embryonic stem cells and cloned into pGEM-T-easy. The diphtheria toxin A gene was cloned into the SacII site. 64 bp to the 3′ of exon 2, the sequence CTCTAT was mutated to CTCGAG, generating an XhoI site. LoxP sites flanking exon 2 were generated by a combination of conventional cloning and recombineering, using a recombineering approach (Liu *et al*, 2003). The loxP PGK-Neo cassette was amplified from pL452 plasmid with flanking AvrII/HindIII sites at each end and cloned into the AvrII site upstream of exon 2. Fragment orientation was confirmed by the generation of 3.5 kb HindIII and 2.0 kb NheI sites, and the vector was recombined in *E. coli* strain SW106 with inducible Cre recombinase expression followed by HindII digestion, generating a single loxP site upstream of exon 2. Into this recombined vector, the FRT-PGK-Neo-FRT-LoxP cassette (amplified from pL451 with flanking XhoI sites) was cloned in the newly generated XhoI site downstream of exon 2, resulting in the "deletion construct". The orientation was confirmed by the generation of 2.2 kb Nhe1 and 3.4 kb BamHI sites. Functionality of the two recombination sites was tested as follows: the FRT site was confirmed by recombination in *E. coli* strain SW105 with inducible FlpE recombinase expression, deleting the FRT-Neo cassette and generating a 1.4 kb BamHI fragment; the resulting plasmid was transformed in *E. coli* strain SW106 with inducible Cre recombinase expression, deleting exon 2 and resulting in a 1.1 kb BamHI fragment. The NotI linearised fragment of the deletion construct was transfected by electroporation into 129/Sv embryonic stem cells. 244 Neomycin-resistant colonies were genotyped by PCR and Southern blotting. Two probes were used: one outside the 3′ end of the deletion construct, with HindIII digestion site giving a single 9 kb fragment for the WT and a 7 kb fragment for the correctly-integrated deletion cassette, and one to the 5′ end of the deletion cassette, again giving the same 9 kb fragment for the Wt but a 3.5 kb fragment for the deletion cassette. Ten colonies showed a correct integration by homologous recombination. These ES cells were injected into blastocysts obtained from pregnant BALB/C mice, and chimeric mice were crossed with C57/Bl6J mice constitutively expressing FlpE recombinase, removing the FRT-Neo cassette. Agouti mice were genotyped by PCR, showing correct insertion of the LoxP sites around exon 2.

Cdk8$^{lox/lox}$ mice were crossed with *Villin-Cre*-$^{ERT2+/-}$ mice to generate the inducible conditional knockout.

Tamoxifen treatment of mice to induce Lox recombination

Mice were first injected intraperitoneally (IP) with 100 μl of 20 mg/ml tamoxifen solution (in corn oil). After the injection, they were fed during 5 days with cookies containing 400 mg tamoxifen citrate per kg diet (Envigo, Ref TD.130859).

Apc/Cdk8 conditional knockout mice

C57BL/6 *Apc$^{lox/lox}$* mice (Colnot *et al*, 2004) were provided by Philippe Jay (IGF, Montpellier). These mice were crossed with *Cdk8$^{lox/lox}$/Villin-Cre*-$^{ERT2+/-}$ to obtain *Apc$^{lox/lox}$/Cdk8$^{lox/lox}$/Villin-Cre*-$^{ERT2\ +/-}$ mice. IP injection with tamoxifen during 5 days induced *Cdk8* exon 2 deletion and *Apc* exon 14 deletion in the intestines of mice containing the *Villin-Cre*-^{ERT2} gene. Small intestine and colon samples from these mice were genotyped and analysed by IHC and Western blotting.

Cdk7/Cdk8 conditional knockout mice

Cdk8$^{lox/lox}$ mice were crossed with RERT mutant mice expressing the inducible Cre-ERT2 from the endogenous *Polr2a* locus (Guerra *et al*, 2003). The Cdk8$^{lox/lox}$ RERT mice were then crossed with Cdk7$^{lox/lox}$ mice (Ganuza *et al*, 2012) to obtain Cdk8$^{lox/lox}$/Cdk7$^{lox/lox}$, RERT mice in which CDK8 and CDK7 proteins should be removed from the whole body after tamoxifen treatment. Animals were sacrificed after tamoxifen treatment and intestinal epithelium was collected as indicated in the Sample preparation section below. Proteins were extracted and analysed by Western blotting.

AOM/DSS-induced colon carcinogenesis

Eleven *Cdk8$^{lox/lox}$* and 11 *Cdk8$^{lox/lox}$/Villin-Cre*-$^{ERT2+/-}$ mice were treated with tamoxifen as described above. 4 days later, mice (*Cdk8$^{lox/lox}$* and *Cdk8$^{-/-}$*) were given a single intraperitoneal injection of AOM (10 mg/kg in 0.9% saline; A5486, Sigma-Aldrich); 5 days later, 2.5% Dextran Sodium Sulfate (DSS; MP Biomedicals) was administered in the drinking water during 5 consecutive days. DSS treatment was repeated two more times with 16 days intervals without DSS for recovery (see scheme, Appendix Fig S1A). Mice were sacrificed 16 days after the third DSS treatment. Colons were flushed with PBS and either used for intestinal epithelium extraction (see Sample preparation for details) or used for IHC studies. Colons used for IHC were fixed overnight in neutral buffered formalin (10%) before paraffin embedding. Briefly, 4 μm thick sections were dewaxed in xylene and rehydrated in graded alcohol baths. Slides were incubated in 3% H_2O_2 for 20 min and washed in PBS to quench endogenous peroxidase activity. Antigen retrieval was performed by boiling slides for 20 min in 10 mM sodium citrate buffer, pH 6.0. Nonspecific binding sites were blocked in blocking buffer (TBS, pH 7.4, 5% dried milk, 0.5% Triton X-100) for 60 min at RT. Sections were incubated with anti-β-catenin antibody diluted in blocking buffer overnight at 4°C. Envision+(Dako) was used as a secondary reagent. Signals were developed with Fast DAB (Sigma-Aldrich). After dehydration, sections were mounted in Pertex (Histolab), imaged using the Nanozoomer-XR Digital slide Scanner C12000-01 (Hamamatsu) and analysed using NDP.view 2 program (Hamamatsu).

CDK8/19 inhibitor treatment of mice

SNX-631(50 mg/kg dissolved in 70% PEG-4000, 30% Propylen Glycol, 100 μl final volume) or solvent alone was administered daily by oral gavage to 10 C57BL6 mice (five females and five males) during 4 or 11 days. After this time, mice were sacrificed, the intestine was extracted and either fixed 24 h in neutral buffered formalin 10%, dehydrated, and embedded in paraffin for IHC, or frozen for RNA or protein extraction.

Small intestine organoids and CRISPR-Cas9-mediated Cdk19 targeting

The genetic background of intestinal organoids and mice used in this study was identical. *Cdk8$^{lox/lox}$* and *Cdk8$^{lox/lox}$/Villin-Cre*-^{ERT2}

© 2022 The Authors

mice were used to obtain small intestine organoids. Establishment, expansion, and maintenance of organoids were performed as described previously (Sato *et al*, 2009). To induce the Cre-mediated recombination of *Cdk8 in vitro*, organoids were cultured during 7 days in medium supplemented with 600 nM 4-Hydroxytamoxifen (Sigma H7904) resuspended in ethanol. Evaluation of knockout efficiency was performed using genotyping, qPCR, and Western blotting.

CRISPR/Cas9-mediated genome editing was employed to remove CDK19 from the organoids. CRISPR single guide RNA (sgRNA) targeting exon 1 in mouse *Cdk19* (CDK19_Mouse: 5′-AAAGTGGGACGCGGCACCTA-3′ from Zhang lab database) was cloned as synthetic dsDNA into lentiCRISPRv2 vector as described (Sanjana *et al*, 2014; provided by F. Zhang, Addgene plasmid #52961). Lentiviruses encoding the sgRNA targeting sequence were produced in HEK 293T cells transfected with LentiCRISPRv2 (+sgRNA *Cdk19*), pMD2.G, and psPAX2. The viral supernatant (collected in organoids culture media) was passed through a 0.45-μm filter and used the same day for infection. Lentiviral-mediated transduction and antibiotic selection was performed as described previously (Onuma *et al*, 2013). Briefly, for lentiviral infection, organoids (5 days after seeding) were diluted into 10 ml of PBS and dissociated into single cells by passing them 10–15 times through a needle with an insulin syringe. A volume containing 1–5 × 10^5 intestinal cells was centrifuged at 300 *g* for 5 min and resuspended with 1 ml of the viral supernatant produced in HEK 293T cells. This mixture (virus + single stem cells) was layered on top of a Matrigel-covered well (12 well plate). 24 h later, virus and dead cells containing media were removed and the Matrigel-attached cells were covered with 200 μl of Matrigel +200 μl of culture medium to create a "sandwich" containing the infected cells inside. After polymerisation of the second Matrigel layer, 1 ml of organoid media per well was used to allow organoid formation inside the Matrigel. 24 h later, Puromycin was added (5 μg/ml) and selection was conducted for 4 days. Once the organoids appeared (4–5 days after seeding the infected single cells), single organoids were picked for clonal expansion. Effects of targeted deletion were verified by sequencing. Genomic DNA was extracted from organoids using KAPA Mouse genotyping kit from Clinisciences (KK7352). The 2 primers used for amplification of the *Cdk19* allele are indicated in the table as CDK19 CRISP-R22 Fw and CDK19 CRISP-R22 Rev (sequencing). They were amplified as follows: 95°C for 5 min; 30 cycles at 95°C for 45″; 57°C for 30″; 72°C for 1 min 30 s. Absence of CDK19 protein in the organoids was also verified by Western blotting. DNA sequencing confirmed the deletion of a fragment of DNA around the sequence corresponding to *Cdk19* sgRNA, and qPCR confirmed the loss of *Cdk19* mRNA.

Mouse intestine epithelium, colon tumour, and organoid sample preparation

For intestine epithelium samples, a fragment of intestine was cut and flushed with PBS. It was incubated for 5–10 min in EDTA-containing buffer (500 ml RPMI, 20 mM Hepes, 1% Penicillin/Streptomycin (Sigma P4333), 12.5 μg/ml DTT, 2 mM EDTA pH 7.4 and 10% FBS) to allow easy detachment of the intestine epithelium. The intestinal tube was opened longitudinally and put over a horizontal plate to allow scrapping of the epithelial cells by trawling two needles in opposite directions over the tissue. Cells were recovered from the plate by wetting them with a small volume (200 μl) of PBS. They were put into an Eppendorf tube where they were spun down to remove most of the PBS. Pellets were snap frozen and conserved at −80°C. Colon tumours were cut from the colon, washed with PBS and frozen in liquid nitrogen.

Frozen intestinal epithelium and colon tumour samples were resuspended in 250 μl of lysis buffer with protease and phosphatase inhibitors (5 mM Tris, pH 7.4, 100 mM NaCl, 50 mM NaF, 40 mM beta-glycerophosphate, 2.5 mM Na-Vanadate, 5 mM EDTA, 1 mM EGTA, 1 mM DTT, 1% Triton X-100 and Protease inhibitor cocktail diluted 1/400 (Sigma P8340)) and, after addition of 100 μl of stainless-steel beads (0.2 mm diameter, 1 lb, Next Advance, SSB02), they were disrupted in a bullet blender storm 24 (Next Advance) by shaking during 4 min at 4°C with an intensity level of 8. The lysate was incubated during 20 more minutes at 4°C (without shaking) and the solubilised proteins were recovered from the supernatant by centrifugation at 16,000 *g* for 20 min at 4°C, frozen in liquid nitrogen and stored at −80°C until use. Protein concentrations were determined by BCA protein assay (Pierce Biotechnology).

For organoids samples, Matrigel was disrupted by pipetting up and down several times the media in each well over the dome of Matrigel. This mix was spun down at 200 *g* for 5 min at 4°C and the pellet was washed twice with 1 ml of PBS. Pellets were snap frozen in liquid nitrogen and kept at −80°C until use.

For organoids extracts, frozen pellets were lysed by incubation at 4°C for 20 min in lysis buffer with protease and phosphatase inhibitors (50 mM Tris, pH 7.4, 100 mM NaCl, 50 mM NaF, 40 mM beta-glycerophosphate, 2.5 mM Na-Vanadate, 5 mM EDTA, 1 mM EGTA, 1 mM DTT, 1% Triton X-100 and Protease inhibitor cocktail (Sigma P8340) diluted 1/400). The solubilised proteins were recovered from the supernatant after centrifugation at 16,000 *g* for 20 min at 4°C, snap frozen in liquid nitrogen and stored at −80°C until use. Protein concentrations were determined by BCA protein assay (Pierce Biotechnology).

Western blotting

For intestinal epithelium, colon tumours, and organoids, 30 μg of total proteins were separated by SDS–polyacrylamide gel electrophoresis (SDS–PAGE; 7% 10% and 12.5% gels) and transferred to Immobilon membranes (Milipore) at 90 volts for 120 min with a wet-blotting apparatus. Membranes were blocked in TBS-T pH 7.6 (20 mM Tris, 140 mM NaCl, 0.1% Tween-20) containing non-fat dry milk (5%), incubated with the primary antibody in TBS-T + 3% BSA for 2 h at RT or over-night at 4°C, washed three times with TBS-T for a total of 15 min, incubated with secondary antibody at 1/10,000 dilution in TBS-T + 5% nonfat dried milk for 30 min at RT, and washed 3 times in TBS-T for a total of 15 min. Signals were detected using Western Lightning Plus-ECL (PerkinElmer) and Amersham Hyperfilm™ ECL (GE Healthcare).

RNA isolation and qRT–PCR

RNA was extracted from organoids and purified using RNeasy mini kit (Qiagen) according to manufacturer's protocol. For reverse transcription (cDNA synthesis), 1 μg of purified RNA in total volume of

 © 2022 The Authors

13 μl, extracted by RNeasy Mini Kit (Qiagen), was mixed with 1 μl of 10 mM dNTPs mix (LifeTechnologies) and 1 μl of 10 μM oligo (dT) 20-primer. Samples were incubated at 65°C for 5 min, transferred to ice. 4 μl of 5× First Strand Buffer, 1 μl of 100 mM DTT, 1 μl of RNase OUT RNase Inhibitor (Invitrogen), and 1 μl of SuperScript® III Reverse Transcriptase (LifeTechnologies) were added to each sample and incubated at 50°C for 1 h. The reaction was inactivated at 70°C for 15 min.

qPCR was performed using LightCycler® 480 II (Roche). The reaction contained 5 μl of a 1/10 dilution of the cDNA obtained after RT (25 ng of cDNA), 1 μl of 10 μM qPCR primer pair, 10 μl 2× Master Mix in a final volume made up to 20 μl with DNase free water. qPCR was conducted at 95°C for 5 min, followed by 45 cycles of 95°C 10 s, 59°C 20 s and 72°C 15 s. The specificity of the reaction was verified by melting curve analysis. β2-microglobulin (B2M) was used as housekeeping gene.

Genomic DNA extraction and genotyping

For colon tumours, genomic DNA was extracted using the KAPA Mouse genotyping kit from Clinisciences (KK7352) directly over 3–4 tumours that had been removed from the colon before the extraction of intestinal epithelium.

Genomic DNA was extracted from intestinal epithelium pellets or organoids using KAPA Mouse genotyping kit from Clinisciences (KK7352).

The forward and reverse primers used for amplification of the *Cdk8* wild-type allele (650 bp), the *Cdk8*$^{Lox/Lox}$ (850 bp), and the fragment obtained after tamoxifen-induced Cre recombination of *Cdk8*$^{Lox/Lox}$ (*Cdk8* Lox + Cre (−); 340 bp) are indicated in the table below as CDK8 Fw (genotyping) and CDK8 Rev (genotyping). These fragments were amplified from genomic DNA using the following PCR protocol: 95°C for 5 min; 35 cycles at 95°C for 15 s; 57°C for 15 s; 72°C for 1 min 30 s. Amplified DNA fragments were migrated in a 1.5% agarose gels and stained with Ethidium bromide for detection.

Primers used for qPCR and genotyping

Gene name	Sequence	Use
Mouse B2M Fw<br>Mouse B2M Rev	5′-GGTCTTTCTGGTGCTTGTCT-3′<br>5′-GCAGTTCAGTATGTTCGGCTT-3′	qRT–PCR
CDK8 (1,2)-3 Fw<br>CDK8 (1,2)-3 Rev	5′-GTGGGAGA@AGGAAGGACGAT<br>5′-GCCATACTTTCCGATCAGCA-3′	qRT–PCR
CDK19 (5–6) Fw<br>CDK19 (5–6) Rev	5′-TTCTCCCCTAAAGCCACTCG-3′<br>5′-ATGGGTTCTGAAGTCAAGAGTT-3′	qRT–PCR
Cyc C (10–11) Fw<br>Cyc C (10–11) Rev	5′-CCGAAACCAAAACCACCTCC-3′<br>5′-TCCCAATATGCTTGACAGAAACA-3′	qRT–PCR
CFTR (1–2) Fw<br>CFTR (1–2) Rev	5′-TAAAAGGGACGAGCCAAAAG-3′<br>5′-CCCTTTCCTCAAAATTGGTG-3′	qRT–PCR
Muc2 (15–16) Fw<br>Muc2 (15–16) Rev	5′-AACAACGAGGACTGCATGTG-3′<br>5′-ACAGGTGCAAATCCCTTGAG-3′	qRT–PCR
Muc3 (2–3) Fw<br>Muc3 (2–3) Rev	5′-GTCCGTGGAAGTGAGTGTGA-3′<br>5′-ATAACCCCTTCATACTCCGGTA-3′	qRT–PCR
CDK8 Fw<br>CDK8 Rev	5′-ACATGCCTTACAGCCTAGTCTTAC-3′<br>5′-CCAAATAAATGTATACTCTGCAAG-3′	genotyping
CDK19 CRISP-R22 Fw<br>CDK19 CRISP-R22 Rev	5′-GAGGAGTCCCTTGCTGAAG-3′<br>5′-CAGTGCCTCCGAGTTAGC-3′	sequencing
APC Genotyping Fw<br>APC Genotyping Rev	5′-CTGTTCTGCAGTATGTTATCA-3′<br>5′-CTATGAGTCAACACAGGATTA-3′	genotyping
Villin-Cre-^{ERT2} Fw<br>Villin-Cre-^{ERT2} Rev	5′-CAAGCCTGGCTCGACGGCC-3′<br>5′-CGCGAACATCTTCAGGTTCT-3′	genotyping
Lgr5+ Fw<br>Lgr5+ Rev	GACAATGCTCTCACAGAC<br>GGAGTGGATTCTATTATTATGG	qRT–PCR
Olfm4 Fw<br>Olfm4 Rev	GCCACTTTCCAATTTCAC<br>GAGCCTCTTCTCATACAC	qRT–PCR
Lyz Fw<br>Lyz Rev	GCCAAGGTCTACAATCGTTGTGAGTTG<br>CAGTCAGCCAGCTTGACACCACG	qRT–PCR
Ang4 Fw<br>Ang4 Rev	CTCTGGCTCAGAATGTAAGGTACGA<br>GAAATCTTTAAAGGCTCGGTACCC	qRT–PCR
Mmp7 Fw<br>Mmp7 Rev	TTTGATGGGCCAGGGAACAC<br>GGAAGTTCACTCCTGCGTCC	qRT–PCR
Spdef Fw<br>Spdef Rev	GCTGGCTCAACAAGGAGAAA<br>TGTGGCTCTCAGACTGGATG	qRT–PCR
Tiff3 Fw<br>Tiff3 Rev	TCAAACCTCTGCAGGAGACA<br>TCAGATCAGCCTTGTGTTGG	qRT–PCR
Muc2 Fw<br>Muc2 Rev	TCCTGACCAAGAGCGAACAC<br>ACAGCACGACAGTCTTCAGG	qRT–PCR
Klf4 Fw<br>Klf4 Rev	GAAATTCGCCCGCTCCGATGA<br>CTGTGTGTTTGCGGTAGTGCC	qRT–PCR
Pou2f3 Fw<br>Pou2f3 Rev	GCAGCAATGAACTCCTCCTC<br>CCTATGTGGAAGGCACGACT	qRT–PCR
DCAMKL1 Fw<br>DCAMKL1 Rev	CAGCCTGGACGAGCTGGTGG<br>TGACCAGTTGGGGTTCACAT	qRT–PCR
Neurog3 Fw<br>Neurog3 Rev	GCTGCTTGACACTGACCCTA<br>ATGAGGCGCCATCCTAGTTC	qRT–PCR
ChromoA Fw<br>ChromoA Rev	CAAGGTGATGAAGTGCGTC<br>GAGAGCCAGGTCTTGAAGTTC	qRT–PCR
Notch1 (1–3) Fw<br>Notch1 (1–3) Rev	CAGGAAAGAGGGCATCAGAG<br>CTGAGGCAAGGATTGGAGTC	qRT–PCR
Hes1 (1–4) Fw<br>Hes1 (1–4) Rev	ACACCGGACAAACCAAAGAC<br>TGATCTGGGTCATGCAGTTG	qRT–PCR

Antibodies

Name	Clone	Source	Species	Cat#
CDK8 (C19)	Polyclonal	Santa Cruz	Goat	sc-1521
CDK19	Polyclonal	Sigma	Rabbit	HPA007053
Cyclin C	Polyclonal	gift	Rabbit	
PCNA	Monoclonal	Santa Cruz	Mouse	sc-56
Lysozyme	Polyclonal	Dako	Rabbit	A0099
Anti-Dclk1	Polyclonal	Abcam	Rabbit	Ab31704
Olfm4	Monoclonal	Cell signaling	Rabbit	39141
β-Catenin	Monoclonal	BD BioSciences	Mouse	610,154
BrdU	Monoclonal	DSHB	Mouse	G3G4

© 2022 The Authors

Name	Clone	Source	Species	Cat#
RNA pol II Ser2P	Monoclonal	JC Andrau's lab	Rat	(3E10)
RNA pol II Ser5P	Monoclonal	JC Andrau's lab	Rat	(3E8)
RNA pol II Ser7P	Monoclonal	JC Andrau's lab	Rat	(4E12)
CDK7	Monoclonal	Santa Cruz	Mouse	sc-7344
Ki-67	SP6	Spring Bioscience	Rabbit	M3064
Ki-67	Monoclonal	Abcam	Rabbit	Ab16667
p53	CM5	Novocastra	Rabbit	NCL-p53-CM5p
Cleaved Caspase 3	ASP176	Cell signaling	Rabbit	9661S
Phospho-Stat1 (Ser727)	Polyclonal	Cell signaling	Rabbit	#9177
CFTR	Monoclonal	Cystic Fibrosis Foundation	Mouse	570
β-Actin	Monoclonal	Sigma	Mouse	a5441
GAPDH	Polyclonal	Sigma	Rabbit	G9545

Cyclin C antibody purification: Rabbit anti-Cyclin C serum was a kind gift from Jacques Piette (IGMM Montpellier, France; Barette *et al*, 2001). Cyclin C-specific antibodies were purified from serum by incubation with a membrane containing Cyclin C protein (ProQinase GmbH). The antibodies were eluted with 200 µl of 0.2 M glycine pH 2.5 and neutralised rapidly with 21 µl of 1 M Tris. RNA Pol II Ser2P (3E10), RNA Pol II Ser5P (3E8), and RNA Pol II Ser7P (4E12) antibodies were a kind gift from Jean-Christophe Andrau's lab (IGMM, CNRS Montpellier, France; Chapman *et al*, 2007).

Immunohistochemistry (IHC) and tissue staining

Whole intestines were flushed with PBS and turned inside-out on a wooden stick. They were collected and fixed 24 h in neutral buffered formalin 10%, dehydrated, and embedded in paraffin.

Organoids were collected and fixed for 1 h in 4% paraformaldehyde at room temperature. They were washed with PBS (×2) and resuspended into 100 µl of Histogel (Fisher Scientific, Ref 12006679), previously thawed in a hot water bath at 60°C. Each drop containing the organoids and Histogel was dried on top of a flat surface and embedded in paraffin. Paraffin-embedded intestines or organoids were cut into 3-µm-thick sections, mounted on slides, then dried at 37°C overnight. Tissue sections were stained with haematoxylin–eosin (HE) with HMS 740 autostainer (MM France) for preliminary analysis. For mucosubstances, tissue sections were stained with Periodic Acid Schiff's (PAS) staining (Bancroft & Stevens, 1982).

For Ki67, p53, and Caspase 3, IHC was performed as described previously (Rahmanzadeh *et al*, 2007), on a VENTANA Discovery Ultra automated staining instrument (Ventana Medical Systems), using VENTANA reagents, according to the manufacturer's instructions. Briefly, slides were de-paraffinised, then epitope retrieval was performed with CC1 solution (cat# 950-124) at high temperature (95–100°C) or for CC2 solution (cat# 950-123) at high temperature (91°C) for a period time that is suitable for each specific antibody. Endogenous peroxidases were blocked with Discovery Inhibitor (cat# 760-4840).

For CDK8 immunostaining, signal enhancement was performed using the rabbit antibody anti-goat (Vector Laboratories, cat#BA-500, 1:2,000) for 16 min at 37°C then using DISCOVERY OmniMap anti-rabbit HRP detection Kit (cat# 05269679001) according to the manufacturer's instructions. The same kit was used for all other rabbit primary antibodies to amplify the signal. Slides were incubated with DAB (cat# 05266645001), then counterstained with haematoxylin II (cat# 790-2208) for 8 min, followed by Bluing reagent (cat# 760-2037) for 4 min. Slides were then dehydrated with Leica autostainer and coverslipped with Pertex mounting medium with CM6 coverslipper (Microm). Brightfield stained slides were digitalised with a Hamamatsu NanoZoomer 2.0-HT scanner and images were visualised with the NDP.view 1.2.47 software.

For Paneth cells, tuft cells, β-catenin, and Brdu detection, the IHCs were performed manually. After deparaffination and rehydration, demasking of antigenic sites was performed by boiling the slides for 20′ in 10 mM Na-Citrate pH 6.4. After cooling down, slides were treated with 3% H_2O_2, 5′ at RT for peroxidase inhibition. Samples were blocked in blocking solution (TBS, 0.5% Triton, 5% dry milk) for 20′. First antibody was diluted 1/400 in blocking solution and the slides were incubated O/N at 4°C in a humid chamber. Slides were then washed with TBS + 0.1% Tween 20 (3 times) and once with TBS without Tween. Secondary antibodies (ImmPRESS™ reagent kit peroxidase anti-rabbit (MP-7451) or anti-Mouse (MP-7402), from Vector Laboratories) were incubated for 30′ at RT. Slides were washed twice with TBS + 0.1% Tween 20 and the final wash was done in H_2O. Peroxidase staining was performed using Sigma Fast DAB tablet set (D4293-50SET). After Haematoxylin staining of the nucleus with Gill's Haematoxylin solution N°2 (CAS 517-28-2) from Santa Cruz (SC-24973), slides were rehydrated and mounted in Pertex^R (Histolab 00811).

Sequence alignment

CDK8 and CDK19 protein sequences from different species were aligned using the BoxShade server.

RNA sequencing

After 7 days of treatment with 600 nM 4-Hydroxytamoxifen, organoids were lysed and RNA was extracted following the Trizol RNA isolation protocol (W.M. Keck Foundation Biotechnology Microarray Resource Laboratory at Yale University) until the end of the phase-separation step. Total RNA cleanup with DNase digestion was performed by addition of 1.5 volumes of absolute ethanol on top of the aqueous phase obtained after the phase-separation and following the Qiagen RNeasy protocol (W.M. Keck Foundation Biotechnology Microarray Resource Laboratory at Yale University). RNA integrity was analysed on Agilent 2100 Bioanalyzer. All conditions were prepared as biological triplicates and sent to BGI Tech Solutions (Hong Kong) Co for library preparation and RNA sequencing. Purification of mRNA from total RNA was achieved using oligo(dT)-

© 2022 The Authors

attached magnetic beads and then fragmented for random hexamer-primed reverse transcription, followed by a second-strand cDNA synthesis. Sequencing was performed with the BGISEQ, SE50 platform to obtain an average of 50 million single-end, 50 bp reads per sample.

Bioinformatic analysis

The raw reads obtained in fastq format were subject to quality control using the FastQC software. The reads passing the quality control were aligned to the mouse reference genome (GRCm38.p6) and the counts per gene were quantified using the tool STAR 2.6.0a(2). The Ensembl mouse genome annotations (release 93) were used to map the reads to each gene and their corresponding transcripts. Differential gene expression analysis was performed in R using the DESeq2 library. MA-plots showing the deferentially expressed genes were generated with an in-house script and the gene set enrichment analysis were performed using the "enrichR" library, in both cases using the R programming language.

Correlation between global gene expression changes of organoids and mouse intestinal single cell RNA-seq data

We compared the results of the transcriptional changes observed by bulk RNA-seq upon double *Cdk8/Cdk19* knockout and gene signatures of each cell type obtained by single cell RNA-seq (Haber *et al*, 2017), using data from the plate-based full-length scRNA-Seq. After clustering and identifying the different cell types, gene signatures were defined as those genes in a given cell type that were significantly more expressed when compared against all other cell types, in a pairwise manner.

Time-lapse microscopy

Organoids (Wt, $Cdk8^{Lox/Lox}$, $Cdk19^{-/-}$ and $Cdk8^{Lox/Lox}/Cdk19^{-/-}$) were treated with 600 nM 4-hydroxytamoxifen during 7 days. Once recombination of the LoxP sites flanking *Cdk8* exon 2 was obtained, images were taken every 4 h during 7 days on an inverted microscope (Axio Observer, Zeiss) equipped with a heated chamber allowing constant temperature (37°C) and CO_2 flow (5% CO_2). CCD camera (Princeton Instruments (Micromax), pixel = 6.7 µm), with 10x/0.3 DRY PH1objective, correction ECPLAN Neofluar, 5.2 mm working distance. Acquisition software was MetaMorph 7.8 (Molecular Devices, LLC). Images were analysed using Image J software to calculate the time for release of mucus in the lumen of the *organoid* (observed as a dark staining in the centre of the organoid).

Forskolin-induced swelling

To remove exon 2 of *Cdk8* from $Cdk8^{lox/lox}$/Villin-Cre-ERT2$^{+/-}$/$Cdk19^{-/-}$, organoids were treated with 600 nM 4-hydroxytamoxifen for 7 days. Once the *Cdk8/Cdk19* double KO was obtained, forskolin-induced swelling was measured as indicated (Dekkers *et al*, 2013). Organoids were transferred to CELLview culture dishes PS 35/10 mm, glass bottom, 4 compartments (Greiner Bio-One, 627870), two days before imaging. Confocal spinning disk (Dragonfly, Andor, Oxford Instruments) microscope equipped with heated chamber allowing constant temperature (37°C) and CO_2 flow (5% CO_2), EMCCD iXon888 camera (Lifer Andor, pixel = 13 µm), objective 10x/0.45 DRY, correction Plan Apo Lambda, 4 mm working distance, was used for imaging, with Fusion acquisition software. Images of a single organoid, previously selected, were taken every 2 min during 20 min after forskolin addition (5 µM, or DMSO vehicle control) to the media. For data analysis, a macro was created using Fiji software. It consisted of recognising and filling the structures imaged through the alexa-488 track, to calculate the increase of total organoid area in single organoids over the different time points.

Statistics

Graphs and statistical analyses were performed using Microsoft Excel 16.50 and GraphPad Prism6 using analyses described in legends.

Data availability

The RNA-sequencing data have been deposited in the Gene Expression Omnibus (GEO, NCBI) repository, and are accessible through GEO Series accession number GSE186377 (https://www.ncbi.nlm.nih.gov/geo/query/acc.cgi?acc=GSE186377).

Expanded View for this article is available online.

Acknowledgements
LA was funded by the Montpellier FHU Cancer programme and by the Fondation pour la Recherche Medicale; GD was funded by the French National Cancer Institute (INCa); SP, CB-P, and FG are CNRS employees; DF, PJ, and LK are Inserm employees. This work was undertaken with support from INCa (PLBIO10-068 and PLBIO15-005), and the LNCC (EL2013.LNCC/DF and EL2018.LNCC/DF). RHEM histology facility was supported by SIRIC Montpellier Cancer Grant INCa_Inserm_DGOS_12553, the European regional development foundation and the Occitanie region (FEDER-FSE 2014-2020 Languedoc Roussillon). We thank Sylvain de Rossi and Volker Baecker of the Montpellier Resources in Imaging (MRI) platform for help with microscopy and analysis. We thank Max Chen and Igor Roninson for providing Senexin B, SNX-631 and for helpful advice, and J.-C. Andrau for providing RNA pol II antibodies.

Author contributions
Susana Prieto: Conceptualization; formal analysis; investigation; writing – review and editing. **Geronimo Dubra:** Data curation; formal analysis; investigation; methodology. **Alain Camasses:** Investigation; methodology. **Ana Bella Aznar:** Methodology. **Christina Begon-Pescia:** Investigation; methodology. **Elisabeth Simboeck:** Methodology. **Nelly Pirot:** Investigation. **François Gerbe:** Investigation. **Lucie Angevin:** Validation. **Philippe Jay:** Conceptualization; methodology. **Liliana Krasinska:** Conceptualization; formal analysis; writing – original draft; writing – review and editing. **Daniel Fisher:** Conceptualization; supervision; funding acquisition; validation; methodology; writing – original draft; project administration; writing – review and editing.

Disclosure and competing interests statement
The authors declare that they have no conflict of interest.

© 2022 The Authors

References

Adler AS, McCleland ML, Truong T, Lau S, Modrusan Z, Soukup TM, Roose-Girma M, Blackwood EM, Firestein R (2012) CDK8 maintains tumor dedifferentiation and embryonic stem cell pluripotency. *Cancer Res* 72: 2129–2139

Andrau J-C, van de Pasch L, Lijnzaad P, Bijma T, Koerkamp MG, van de Peppel J, Werner M, Holstege FCP (2006) Genome-wide location of the coactivator mediator: Binding without activation and transient Cdk8 interaction on DNA. *Mol Cell* 22: 179–192

Audetat KA, Galbraith MD, Odell AT, Lee T, Pandey A, Espinosa JM, Dowell RD, Taatjes DJ (2017) A Kinase-independent role for cyclin-dependent Kinase 19 in p53 response. *Mol Cell Biol* 37: e00626-16

Bacevic K, Prieto S, Caruso S, Camasses A, Dubra G, Ursic-Bedoya J, Lozano A, Butterworth J, Zucman-Rossi J, Hibner U *et al* (2019) CDK8 and CDK19 kinases have non-redundant oncogenic functions in hepatocellular carcinoma. *bioRxiv* https://doi.org/10.1101/789586 [PREPRINT]

Bancerek J, Poss ZC, Steinparzer I, Sedlyarov V, Pfaffenwimmer T, Mikulic I, Dölken L, Strobl B, Müller M, Taatjes DJ *et al* (2013) CDK8 kinase phosphorylates transcription factor STAT1 to selectively regulate the interferon response. *Immunity* 38: 250–262

Bancroft J, Stevens A (1982) *Theory and practice of histological techniques*, pp 188–190. New York: Churchill Livingstone

Bankhead P, Loughrey MB, Fernández JA, Dombrowski Y, McArt DG, Dunne PD, McQuaid S, Gray RT, Murray LJ, Coleman HG *et al* (2017) QuPath: Open source software for digital pathology image analysis. *Sci Rep* 7: 16878

Barette C, Jariel-Encontre I, Piechaczyk M, Piette J (2001) Human cyclin C protein is stabilized by its associated kinase cdk8, independently of its catalytic activity. *Oncogene* 20: 551–562

Bergeron P, Koehler MFT, Blackwood EM, Bowman K, Clark K, Firestein R, Kiefer JR, Maskos K, McCleland ML, Orren L *et al* (2016) Design and development of a series of potent and selective type II inhibitors of CDK8. *ACS Med Chem Lett* 7: 595–600

Chapman RD, Heidemann M, Albert TK, Mailhammer R, Flatley A, Meisterernst M, Kremmer E, Eick D (2007) Transcribing RNA polymerase II is phosphorylated at CTD residue serine-7. *Science* 318: 1780–1782

Chen M, Liang J, Ji H, Yang Z, Altilia S, Hu B, Schronce A, McDermott MSJ, Schools GP, Lim C *et al* (2017) CDK8/19 mediator kinases potentiate induction of transcription by NFκB. *Proc Natl Acad Sci U S A* 114: 10208–10213

Chen M, Li J, Liang J, Thompson ZS, Kathrein K, Broude EV, Roninson IB (2019) Systemic toxicity reported for CDK8/19 inhibitors CCT251921 and MSC2530818 is not due to target inhibition. *Cell* 8: 1413

Clarke PA, Ortiz-Ruiz M-J, TePoele R, Adeniji-Popoola O, Box G, Court W, Czasch S, Bawab SE, Esdar C, Ewan K *et al* (2016) Assessing the mechanism and therapeutic potential of modulators of the human mediator complex-associated protein kinases. *Elife* 5: e20722

Clevers H (2016) Modeling development and disease with organoids. *Cell* 165: 1586–1597

Colnot S, Niwa-Kawakita M, Hamard G, Godard C, Plenier SL, Houbron C, Romagnolo B, Berrebi D, Giovannini M, Perret C (2004) Colorectal cancers in a new mouse model of familial adenomatous polyposis: influence of genetic and environmental modifiers. *Lab Invest* 84: 1619–1630

Corvol H, Blackman SM, Boëlle P-Y, Gallins PJ, Pace RG, Stonebraker JR, Accurso FJ, Clement A, Collaco JM, Dang H *et al* (2015) Genome-wide association meta-analysis identifies five modifier loci of lung disease severity in cystic fibrosis. *Nat Commun* 6: 8382

Dale T, Clarke PA, Esdar C, Waalboer D, Adeniji-Popoola O, Ortiz-Ruiz M-J, Mallinger A, Samant RS, Czodrowski P, Musil D *et al* (2015) A selective chemical probe for exploring the role of CDK8 and CDK19 in human disease. *Nat Chem Biol* 11: 973–980

Dannappel MV, Zhu D, Sun X, Chua HK, Poppelaars M, Suehiro M, Khadka S, Lim Kam Sian TC, Sooraj D, Loi M *et al* (2022) Mediator kinase regulates intestinal differentiation and homeostasis via the chromatin remodeling complex SWI/SNF. *J Clin Invest* 132: e158593

Dekkers JF, Wiegerinck CL, de Jonge HR, Bronsveld I, Janssens HM, de Winter-de Groot KM, Brandsma AM, de Jong NWM, Bijvelds MJC, Scholte BJ *et al* (2013) A functional CFTR assay using primary cystic fibrosis intestinal organoids. *Nat Med* 19: 939–945

Ding X, Sharko AC, McDermott MSJ, Schools GP, Chumanevich A, Ji H, Li J, Zhang L, Mack ZT, Sikirzhytski V *et al* (2022) Inhibition of CDK8/19 mediator kinase potentiates HER2-targeting drugs and bypasses resistance to these agents *in vitro* and *in vivo*. *Proc Natl Acad Sci U S A* 119: e2201073119

Donner AJ, Szostek S, Hoover JM, Espinosa JM (2007) CDK8 is a stimulus-specific positive coregulator of p53 target genes. *Mol Cell* 27: 121–133

Donner AJ, Ebmeier CC, Taatjes DJ, Espinosa JM (2010) CDK8 is a positive regulator of transcriptional elongation within the serum response network. *Nat Struct Mol Biol* 17: 194–201

Ehre C, Ridley C, Thornton DJ (2014) Cystic fibrosis: an inherited disease affecting mucin-producing organs. *Int J Biochem Cell Biol* 52: 136–145

El Khattabi L, Zhao H, Kalchschmidt J, Young N, Jung S, Van Blerkom P, Kieffer-Kwon P, Kieffer-Kwon K-R, Park S, Wang X *et al* (2019) A pliable mediator acts as a functional rather than an architectural bridge between promoters and enhancers. *Cell* 178: 1145–1158

Elmlund H, Baraznenok V, Lindahl M, Samuelsen CO, Koeck PJ, Holmberg S, Hebert H, Gustafsson CM (2006) The cyclin-dependent kinase 8 module sterically blocks mediator interactions with RNA polymerase II. *Proc Natl Acad Sci U S A* 103: 15788–15793

Firestein R, Bass AJ, Kim SY, Dunn IF, Silver SJ, Guney I, Freed E, Ligon AH, Vena N, Ogino S *et al* (2008) CDK8 is a colorectal cancer oncogene that regulates beta-catenin activity. *Nature* 455: 547–551

Galbraith MD, Allen MA, Bensard CL, Wang X, Schwinn MK, Qin B, Long HW, Daniels DL, Hahn WC, Dowell RD *et al* (2013) HIF1A employs CDK8-mediator to stimulate RNAPII elongation in response to hypoxia. *Cell* 153: 1327–1339

Gallagher AM, Gottlieb RA (2001) Proliferation, not apoptosis, alters epithelial cell migration in small intestine of CFTR null mice. *Am J Physiol Gastrointest Liver Physiol* 281: G681–G687

Ganuza M, Saiz-Ladera C, Canamero M, Gomez G, Schneider R, Blasco MA, Pisano D, Paramio JM, Santamaria D, Barbacid M (2012) Genetic inactivation of Cdk7 leads to cell cycle arrest and induces premature aging due to adult stem cell exhaustion. *EMBO J* 31: 2498–2510

Gerbe F, Brulin B, Makrini L, Legraverend C, Jay P (2009) DCAMKL-1 expression identifies tuft cells rather than stem cells in the adult mouse intestinal epithelium. *Gastroenterology* 137: 2179–2180

Gerbe F, van Es JH, Makrini L, Brulin B, Mellitzer G, Robine S, Romagnolo B, Shroyer NF, Bourgaux JF, Pignodel C *et al* (2011) Distinct ATOH1 and Neurog3 requirements define tuft cells as a new secretory cell type in the intestinal epithelium. *J Cell Biol* 192: 767–780

Gerbe F, Sidot E, Smyth DJ, Ohmoto M, Matsumoto I, Dardalhon V, Cesses P, Garnier L, Pouzolles M, Brulin B *et al* (2016) Intestinal epithelial tuft cells initiate type 2 mucosal immunity to helminth parasites. *Nature* 529: 226–230

 © 2022 The Authors

Guerra C, Mijimolle N, Dhawahir A, Dubus P, Barradas M, Serrano M, Campuzano V, Barbacid M (2003) Tumor induction by an endogenous K-ras oncogene is highly dependent on cellular context. *Cancer Cell* 4: 111 – 120

Haber AL, Biton M, Rogel N, Herbst RH, Shekhar K, Smillie C, Burgin G, Delorey TM, Howitt MR, Katz Y *et al* (2017) A single-cell survey of the small intestinal epithelium. *Nature* 551: 333 – 339

Hodos RA, Strub MD, Ramachandran S, Li L, McCray PB, Dudley JT (2020) Integrative genomic meta-analysis reveals novel molecular insights into cystic fibrosis and ΔF508-CFTR rescue. *Sci Rep* 10: 20553

Hofmann MH, Mani R, Engelhardt H, Impagnatiello MA, Carotta S, Kerenyi M, Lorenzo-Herrero S, Böttcher J, Scharn D, Arnhof H *et al* (2020) Selective and potent CDK8/19 inhibitors enhance NK-cell activity and promote tumor surveillance. *Mol Cancer Ther* 19: 1018 – 1030

Jeronimo C, Langelier M-F, Bataille AR, Pascal JM, Pugh BF, Robert F (2016) Tail and kinase modules differently regulate core mediator recruitment and function *in vivo*. *Mol Cell* 64: 455 – 466

Johannessen L, Sundberg TB, O'Connell DJ, Kolde R, Berstler J, Billings KJ, Khor B, Seashore-Ludlow B, Fassl A, Russell CN *et al* (2017) Small-molecule studies identify CDK8 as a regulator of IL-10 in myeloid cells. *Nat Chem Biol* 13: 1102 – 1108

Kapoor A, Goldberg MS, Cumberland LK, Ratnakumar K, Segura MF, Emanuel PO, Menendez S, Vardabasso C, Leroy G, Vidal CI *et al* (2010) The histone variant macroH2A suppresses melanoma progression through regulation of CDK8. *Nature* 468: 1105 – 1109

Knuesel MT, Meyer KD, Donner AJ, Espinosa JM, Taatjes DJ (2009) The human CDK8 subcomplex is a histone kinase that requires Med12 for activity and can function independently of mediator. *Mol Cell Biol* 29: 650 – 661

Koehler MF, Bergeron P, Blackwood EM, Bowman K, Clark KR, Firestein R, Kiefer JR, Maskos K, McCleland ML, Orren L *et al* (2016) Development of a potent, specific CDK8 kinase inhibitor which phenocopies CDK8/19 knockout cells. *ACS Med Chem Lett* 7: 223 – 228

Kuchin S, Yeghiayan P, Carlson M (1995) Cyclin-dependent protein kinase and cyclin homologs SSN3 and SSN8 contribute to transcriptional control in yeast. *Proc Natl Acad Sci U S A* 92: 4006 – 4010

Li N, Fassl A, Chick J, Inuzuka H, Li X, Mansour MR, Liu L, Wang H, King B, Shaik S *et al* (2014) Cyclin C is a haploinsufficient tumour suppressor. *Nat Cell Biol* 16: 1080 – 1091

Liao SM, Zhang J, Jeffery DA, Koleske AJ, Thompson CM, Chao DM, Viljoen M, van Vuuren HJ, Young RA (1995) A kinase-cyclin pair in the RNA polymerase II holoenzyme. *Nature* 374: 193 – 196

Liu P, Jenkins NA, Copeland NG (2003) A highly efficient recombineering-based method for generating conditional knockout mutations. *Genome Res* 13: 476 – 484

Loncle N, Boube M, Joulia L, Boschiero C, Werner M, Cribbs DL, Bourbon HM (2007) Distinct roles for mediator Cdk8 module subunits in drosophila development. *EMBO J* 26: 1045 – 1054

Lynch CJ, Bernad R, Martínez-Val A, Shahbazi MN, Nóbrega-Pereira S, Calvo I, Blanco-Aparicio C, Tarantino C, Garreta E, Richart-Ginés L *et al* (2020) Global hyperactivation of enhancers stabilizes human and mouse naive pluripotency through inhibition of CDK8/19 mediator kinases. *Nat Cell Biol* 22: 1223 – 1238

Ma T, Thiagarajah JR, Yang H, Sonawane ND, Folli C, Galietta LJV, Verkman AS (2002) Thiazolidinone CFTR inhibitor identified by high-throughput screening blocks cholera toxin–induced intestinal fluid secretion. *J Clin Invest* 110: 1651 – 1658

Malik S, Roeder RG (2010) The metazoan mediator co-activator complex as an integrative hub for transcriptional regulation. *Nat Rev Genet* 11: 761 – 772

el Marjou F, Janssen KP, Chang BH, Li M, Hindie V, Chan L, Louvard D, Chambon P, Metzger D, Robine S (2004) Tissue-specific and inducible Cre-mediated recombination in the gut epithelium. *Genesis* 39: 186 – 193

McCleland ML, Soukup TM, Liu SD, Esensten JH, de Sousa e Melo F, Yaylaoglu M, Warming S, Roose-Girma M, Firestein R (2015) Cdk8 deletion in the Apc(min) murine tumour model represses EZH2 activity and accelerates tumourigenesis. *J Pathol* 237: 508 – 519

McDermott MSJ, Chumanevich AA, Lim C, Liang J, Chen M, Altilia S, Oliver D, Rae JM, Shtutman M, Kiaris H *et al* (2017) Inhibition of CDK8 mediator kinase suppresses estrogen dependent transcription and the growth of estrogen receptor positive breast cancer. *Oncotarget* 8: 12558 – 12575

Menzl I, Zhang T, Berger-Becvar A, Grausenburger R, Heller G, Prchal-Murphy M, Edlinger L, Knab VM, Uras IZ, Grundschober E *et al* (2019) A kinase-independent role for CDK8 in BCR-ABL1^{+} leukemia. *Nat Commun* 10: 4741

Morris EJ, Ji JY, Yang F, Di Stefano L, Herr A, Moon NS, Kwon EJ, Haigis KM, Naar AM, Dyson NJ (2008) E2F1 represses beta-catenin transcription and is antagonized by both pRB and CDK8. *Nature* 455: 552 – 556

Nakamura A, Nakata D, Kakoi Y, Kunitomo M, Murai S, Ebara S, Hata A, Hara T (2018) CDK8/19 inhibition induces premature G1/S transition and ATR-dependent cell death in prostate cancer cells. *Oncotarget* 9: 13474 – 13487

Onuma K, Ochiai M, Orihashi K, Takahashi M, Imai T, Nakagama H, Hippo Y (2013) Genetic reconstitution of tumorigenesis in primary intestinal cells. *Proc Natl Acad Sci U S A* 110: 11127 – 11132

Pelish HE, Liau BB, Nitulescu II, Tangpeerachaikul A, Poss ZC, Da Silva DH, Caruso BT, Arefolov A, Fadeyi O, Christie AL *et al* (2015) Mediator kinase inhibition further activates super-enhancer-associated genes in AML. *Nature* 526: 273 – 276

Porter DC, Farmaki E, Altilia S, Schools GP, West DK, Chen M, Chang BD, Puzyrev AT, Lim CU, Rokow-Kittell R *et al* (2012) Cyclin-dependent kinase 8 mediates chemotherapy-induced tumor-promoting paracrine activities. *Proc Natl Acad Sci U S A* 109: 13799 – 13804

Poss ZC, Ebmeier CC, Odell AT, Tangpeerachaikul A, Lee T, Pelish HE, Shair MD, Dowell RD, Old WM, Taatjes DJ (2016) Identification of mediator kinase substrates in human cells using Cortistatin A and quantitative Phosphoproteomics. *Cell Rep* 15: 436 – 450

Postlmayr A, Dumeau CE, Wutz A (2020) Cdk8 is required for establishment of H3K27me3 and gene repression by Xist and mouse development. *Development* 147: dev175141

Rahmanzadeh R, Huttmann G, Gerdes J, Scholzen T (2007) Chromophore-assisted light inactivation of pKi-67 leads to inhibition of ribosomal RNA synthesis. *Cell Prolif* 40: 422 – 430

Ramachandran S, Karp PH, Jiang P, Ostedgaard LS, Walz AE, Fisher JT, Keshavjee S, Lennox KA, Jacobi AM, Rose SD *et al* (2012) A microRNA network regulates expression and biosynthesis of wild-type and ΔF508 mutant cystic fibrosis transmembrane conductance regulator. *Proc Natl Acad Sci U S A* 109: 13362 – 13367

Rickert P, Corden JL, Lees E (1999) Cyclin C/CDK8 and cyclin H/CDK7/p36 are biochemically distinct CTD kinases. *Oncogene* 18: 1093 – 1102

Rocha PP, Scholze M, Bleiss W, Schrewe H (2010) Med12 is essential for early mouse development and for canonical Wnt and Wnt/PCP signaling. *Development* 137: 2723 – 2731

Sanjana NE, Shalem O, Zhang F (2014) Improved vectors and genome-wide libraries for CRISPR screening. *Nat Methods* 11: 783 – 784

Sato S, Tomomori-Sato C, Parmely TJ, Florens L, Zybailov B, Swanson SK, Banks CA, Jin J, Cai Y, Washburn MP *et al* (2004) A set of consensus mammalian mediator subunits identified by multidimensional protein identification technology. *Mol Cell* 14: 685 – 691

© 2022 The Authors

Sato T, Vries RG, Snippert HJ, van de Wetering M, Barker N, Stange DE, van Es JH, Abo A, Kujala P, Peters PJ *et al* (2009) Single Lgr5 stem cells build crypt-villus structures *in vitro* without a mesenchymal niche. *Nature* 459: 262–265

Schiemann K, Mallinger A, Wienke D, Esdar C, Poeschke O, Busch M, Rohdich F, Eccles SA, Schneider R, Raynaud FI *et al* (2016) Discovery of potent and selective CDK8 inhibitors from an HSP90 pharmacophore. *Bioorg Med Chem Lett* 26: 1443–1451

Steinparzer I, Sedlyarov V, Rubin JD, Eislmayr K, Galbraith MD, Levandowski CB, Vcelkova T, Sneezum L, Wascher F, Amman F *et al* (2019) Transcriptional responses to IFN-γ require mediator kinase-dependent pause release and mechanistically distinct CDK8 and CDK19 functions. *Mol Cell* 76: 485–499

Tsai KL, Sato S, Tomomori-Sato C, Conaway RC, Conaway JW, Asturias FJ (2013) A conserved mediator-CDK8 kinase module association regulates mediator-RNA polymerase II interaction. *Nat Struct Mol Biol* 20: 611–619

Tsutsui T, Umemura H, Tanaka A, Mizuki F, Hirose Y, Ohkuma Y (2008) Human mediator kinase subunit CDK11 plays a negative role in viral activator VP16-dependent transcriptional regulation. *Genes Cells* 13: 817–826

VanDussen KL, Carulli AJ, Keeley TM, Patel SR, Puthoff BJ, Magness ST, Tran IT, Maillard I, Siebel C, Kolterud Å *et al* (2012) Notch signaling modulates proliferation and differentiation of intestinal crypt base columnar stem cells. *Development* 139: 488–497

Westerling T, Kuuluvainen E, Makela TP (2007) Cdk8 is essential for preimplantation mouse development. *Mol Cell Biol* 27: 6177–6182

Wright FA, Strug LJ, Doshi VK, Commander CW, Blackman SM, Sun L, Berthiaume Y, Cutler D, Cojocaru A, Collaco JM *et al* (2011) Genome-wide association and linkage identify modifier loci of lung disease severity in cystic fibrosis at 11p13 and 20q13.2. *Nat Genet* 43: 539–546

Zhao L, Samuels T, Winckler S, Korgaonkar C, Tompkins V, Horne MC, Quelle DE (2003) Cyclin G1 has growth inhibitory activity linked to the ARF-Mdm2-p53 and pRb tumor suppressor pathways. *Mol Cancer Res* 1: 195–206

License: This is an open access article under the terms of the Creative Commons Attribution License, which permits use, distribution and reproduction in any medium, provided the original work is properly cited.

© 2022 The Authors

Expanded View Figures

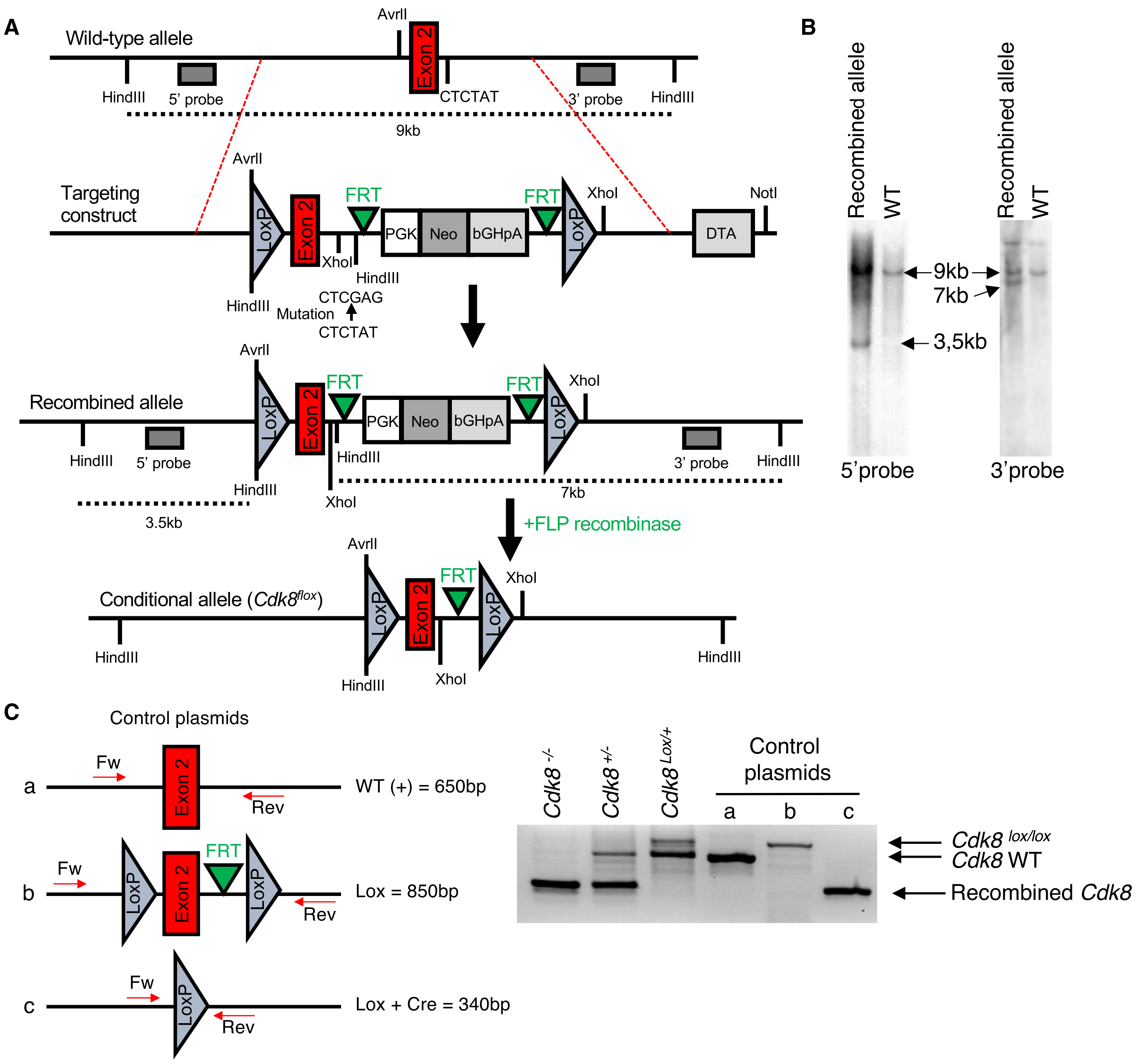

Figure EV1. Mouse *Cdk8* conditional knockout by Lox/Cre targeting of exon 2.

A Schematic representation of the strategy used for the generation of *Cdk8*^{Lox} alleles from a genomic fragment of mouse enclosing exon 2 of the *Cdk8* gene. See Materials and Methods section for details.

B Southern blot analysis of genomic DNA obtained from mice carrying the *Cdk8*^{Lox} and *Cdk8*^{WT} alleles. DNA was digested with HindIII and probed with 2 different probes (5′ and 3′), whose positions are shown in the scheme in (A). The length of the fragments obtained after HindIII digestion of the wild type and the recombinant alleles are indicated (see also the scheme in (A)).

C Left, scheme representing the control plasmids (a, b, and c) for WT, floxed and recombined *Cdk8* exon 2. The position of the oligos (Fw and Rev) used for PCR amplification of genomic DNA and control plasmids is indicated. Right, genotyping of *Cdk8* exon 2 in the mouse intestinal epithelium. All mice were treated with tamoxifen to induce recombination of the LoxP sites. The recombined fragment appears as a 340 bp band in the *Cdk8*$^{-/-}$ and *Cdk8*$^{+/-}$ mice (*VillinCre*^{ERT2} recombinase-positive), and is absent in the *Cdk8*$^{Lox/+}$ mouse that does not contain the *VillinCre*^{ERT2} gene.

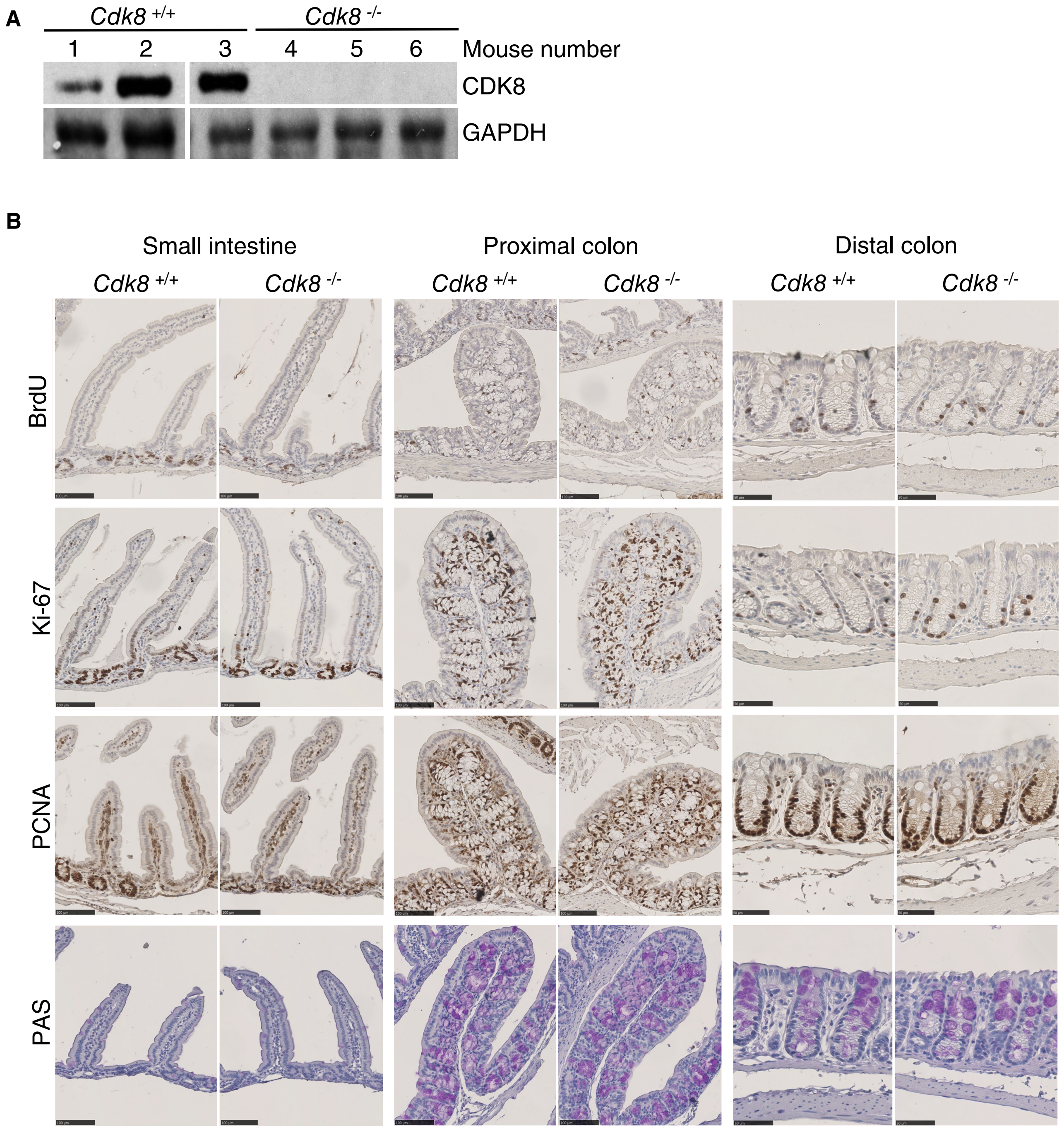

Figure EV2. CDK8 is not required for cell proliferation nor differentiation in mouse intestine.

A WB analysis of mouse intestine epithelium showing the absence of CDK8 protein 2 months after tamoxifen feeding. Mice 1, 2, and 3 did not have the *VillinCre*^{ERT2} gene; mice 4, 5, and 6 had the *VillinCre*^{ERT2}. GAPDH protein was used as loading control.

B Representative immunohistochemistry images of Fig 1B. Small intestine, proximal and distal colon samples were stained for BrdU, Ki-67 or PCNA antibodies. Goblet cells were detected with PAS staining. Scale bars: 100 μm for small intestine and distal colon, 50 μm for proximal colon. Twenty mice were used for the experiment (10 males and 10 females).

Source data are available online for this figure.

 © 2022 The Authors

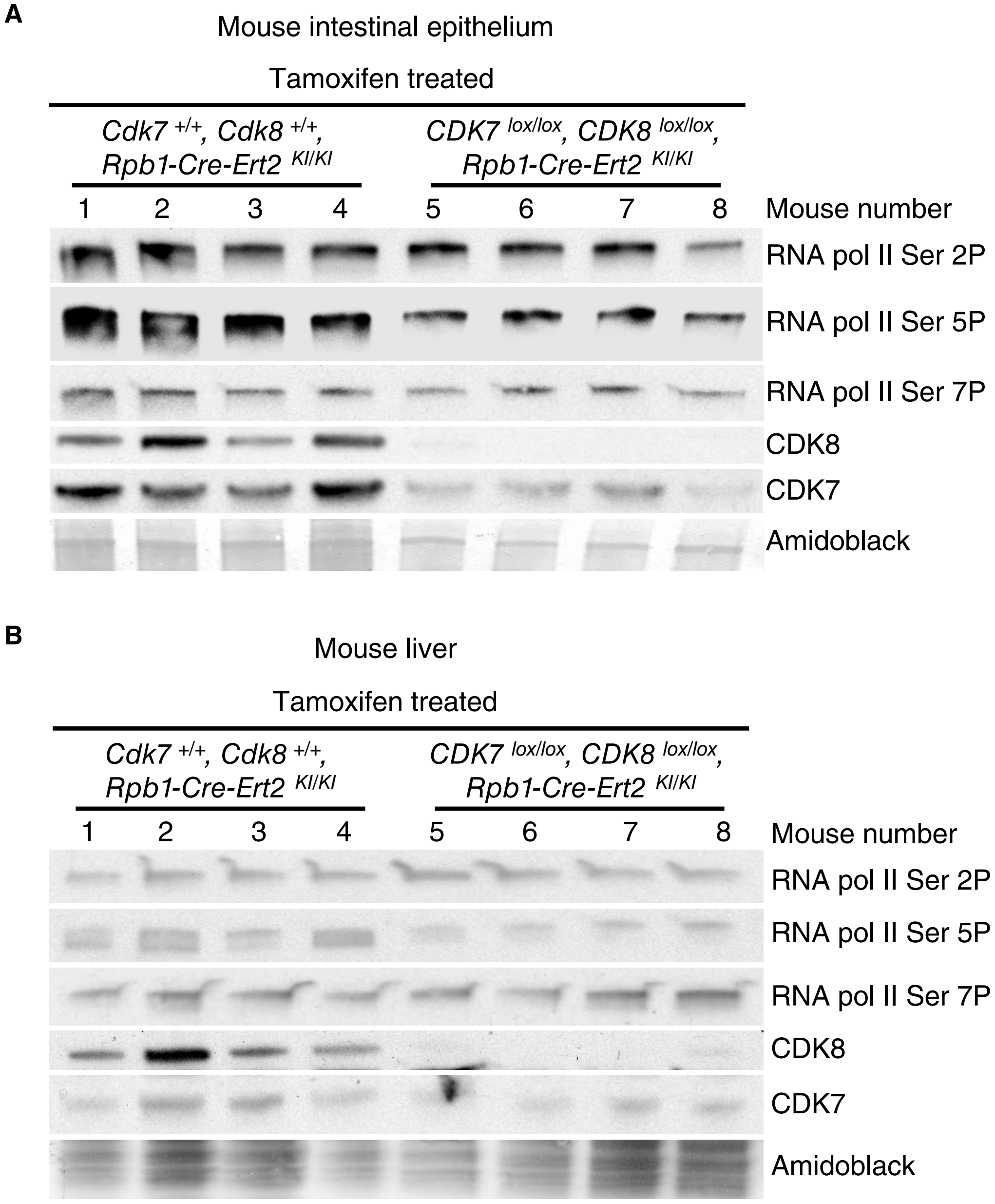

Figure EV3. Effects of CDK8 and CDK7 knockout on RNA pol II CTD phosphorylation.

A, B (A) WB analysis of the indicated proteins in mouse intestinal epithelium samples from WT and *Cdk7*$^{lox/lox}$, *Cdk8*$^{lox/lox}$, *Rpb-Cre-Ert2*$^{KI/KI}$ mice after tamoxifen treatment. Amido-black staining was used as loading control. Sixteen mice of each genotype (eight males and eight females) were used for this experiment. (B) As A but for liver.

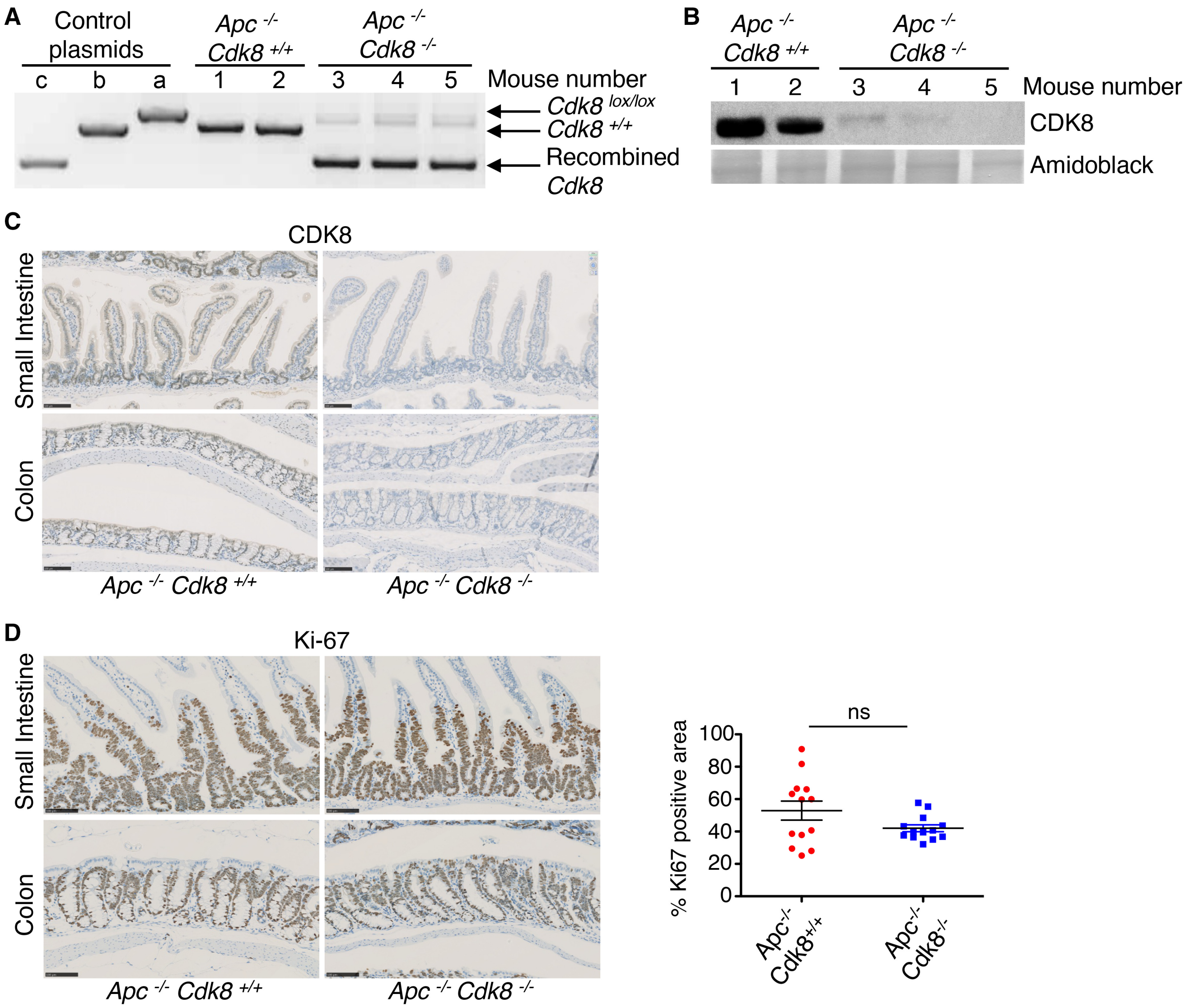

Figure EV4. CDK8 deletion does not prevent Apc-loss-dependent tumourigenesis in mouse intestine.

A Genotyping confirms the loss of *Cdk8* exon 2 in intestine epithelium from $Apc^{-/-}/Cdk8^{-/-}$ mice. Control plasmids (a, b, and c) are described in Fig EV1C.

B WB with intestine epithelium samples from mice presented in (A) confirm the absence of CDK8 protein in $Cdk8^{-/-}$ mice. Amido-black staining was used as loading control.

C, D IHC staining of (C) CDK8 and (D) Ki-67 in small intestine and colon samples from $Apc^{-/-}/Cdk8^{+/+}$ and $Apc^{-/-}/Cdk8^{-/-}$ mice. Scale bars, 100 μm. (D), right: quantification of the Ki-67 positive area (% of the total area of the intestine presenting positive staining, quantified using QuPath (Bankhead *et al*, 2017) and Image J software; mean ± SD). Two-tailed *P*-value of unpaired *t*-test is indicated: ns, not significant ($P > 0.05$; $n = 6$ biological replicates).

Figure EV5. CDK8 and CDK19 are largely redundant but regulate a subset of genes in an opposite manner.

A Comparison of the Log 2 of estimated fold change in expression for each individual gene using a spike-in normalisation method vs. the internal Deseq2 normalisation (library-normalised). From left to right the results for $Cdk8^{-/-}$, $Cdk19^{-/-}$, and $Cdk8^{-/-}/Cdk19^{-/-}$ organoids. Each black dot represents a gene; the dashed red line indicates the identity diagonal; in blue, the linear regression of the data described by the equation shown in the plot.

B, C Standard deviation from the replicates of each gene (B) and mean of the normalised read counts of each gene (C) in the single *Cdk8* and *Cdk19* knockouts vs the *Cdk8/Cdk19* double knockout. Each grey point represents a gene; genes differentially expressed only in a single knockout are highlighted in black. The identity diagonal is represented as a red dashed line.

D, E PCA (Principal component analysis) plots displaying the two axes that explain 72 and 77% of the variance between differentially expressed genes in *Cdk8* knockout (D) or *Cdk19* knockout (E), respectively, and the other genotypes.

 © 2022 The Authors

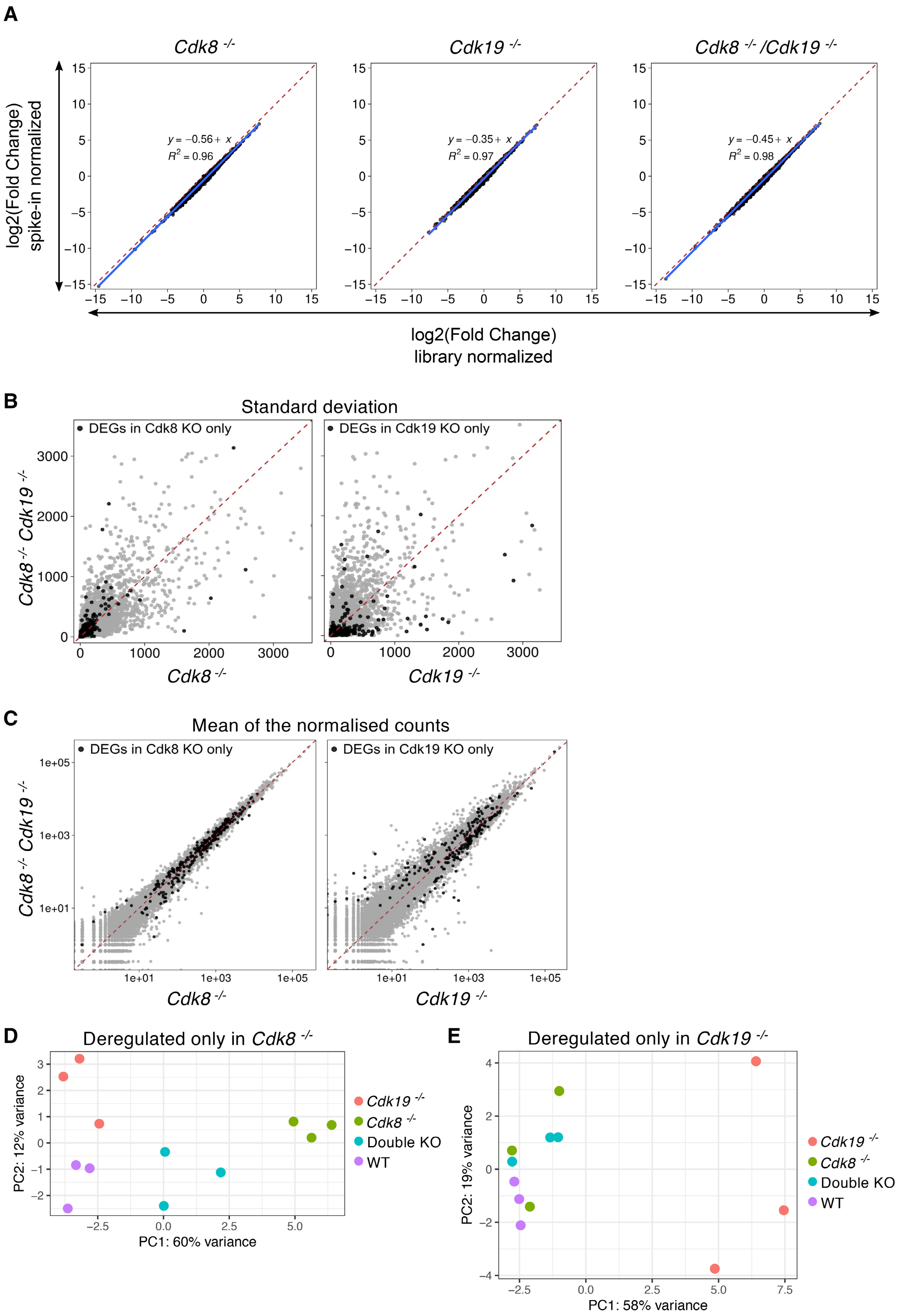
A
Cdk8 -/-
Cdk19 -/-
Cdk8 -/- /Cdk19 -/-
y = −0.56 + x
R2 = 0.96
y = −0.35 + x
R2 = 0.97
y = −0.45 + x
R2 = 0.98
log2(Fold Change) spike-in normalized
log2(Fold Change) library normalized
B
Standard deviation
DEGs in Cdk8 KO only
DEGs in Cdk19 KO only
Cdk8 -/- Cdk19 -/-
Cdk8 -/-
Cdk19 -/-
C
Mean of the normalised counts
DEGs in Cdk8 KO only
DEGs in Cdk19 KO only
Cdk8 -/- Cdk19 -/-
Cdk8 -/-
Cdk19 -/-
D
Deregulated only in Cdk8 -/-
PC2: 12% variance
PC1: 60% variance
Cdk19 -/-
Cdk8 -/-
Double KO
WT
E
Deregulated only in Cdk19 -/-
PC2: 19% variance
PC1: 58% variance
Cdk19 -/-
Cdk8 -/-
Double KO
WT

Figure EV5.

Appendix for:
CDK8 and CDK19 act redundantly to control the CFTR pathway in the intestinal epithelium

Contents .... Page

Appendix Fig S1. CDK8 loss does not affect chemically-induced intestinal carcinogenesis........... 2

Appendix Fig S2. Amino acid sequence conservation of CDK8 and its paralogue CDK19 between different vertebrates... 3

Appendix Fig S3. Cdk8/Cdk19 KO organoids are counter-selected. 4

Appendix Fig S4. Cdk8/Cdk19 double knockout does not significantly affect intestinal cell differentiation. 5

Appendix Fig S5. CDK8/19 inhibition is well tolerated by mice. 6

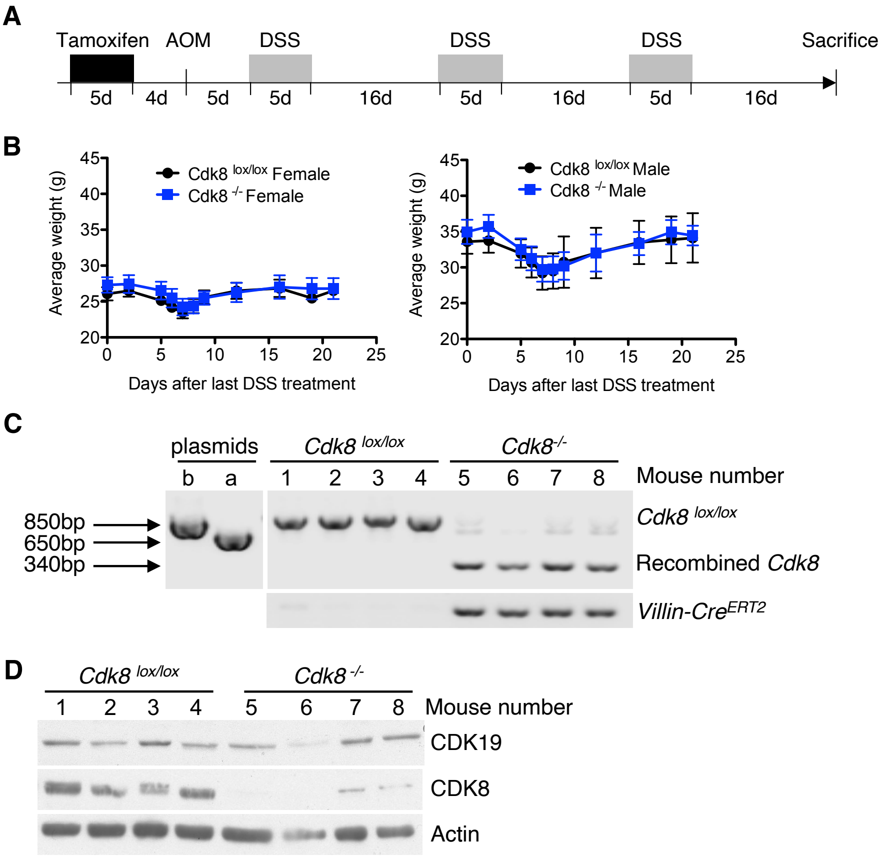

Appendix Fig S1. CDK8 loss does not affect chemically-induced intestinal carcinogenesis.

A Scheme showing the steps of the AOM/DSS carcinogenesis experiment. See Materials and Methods for more detailed information.

B Graphs showing female (left; n=6 mice in both groups*)* and male (right; n=5 mice in both groups) weight evolution over 21 days following the last DSS treatment.

C Genotyping after AOM/DSS treatment confirms the recombination and loss of *Cdk8* exon 2 in colon tumors from *Cdk8* $^{-/-}$ mice. Control plasmids (a and b), are described in Fig EV1C. PCR amplification with *Villin-Cre*^{ERT2}-specific primers confirms the presence of the *Cre*^{ERT2} recombinase gene.

D WB with the same colon tumour samples presented in (C) confirm the disappearance of CDK8 protein in the *Cdk8* $^{-/-}$ mice. β-actin was used as the loading control.

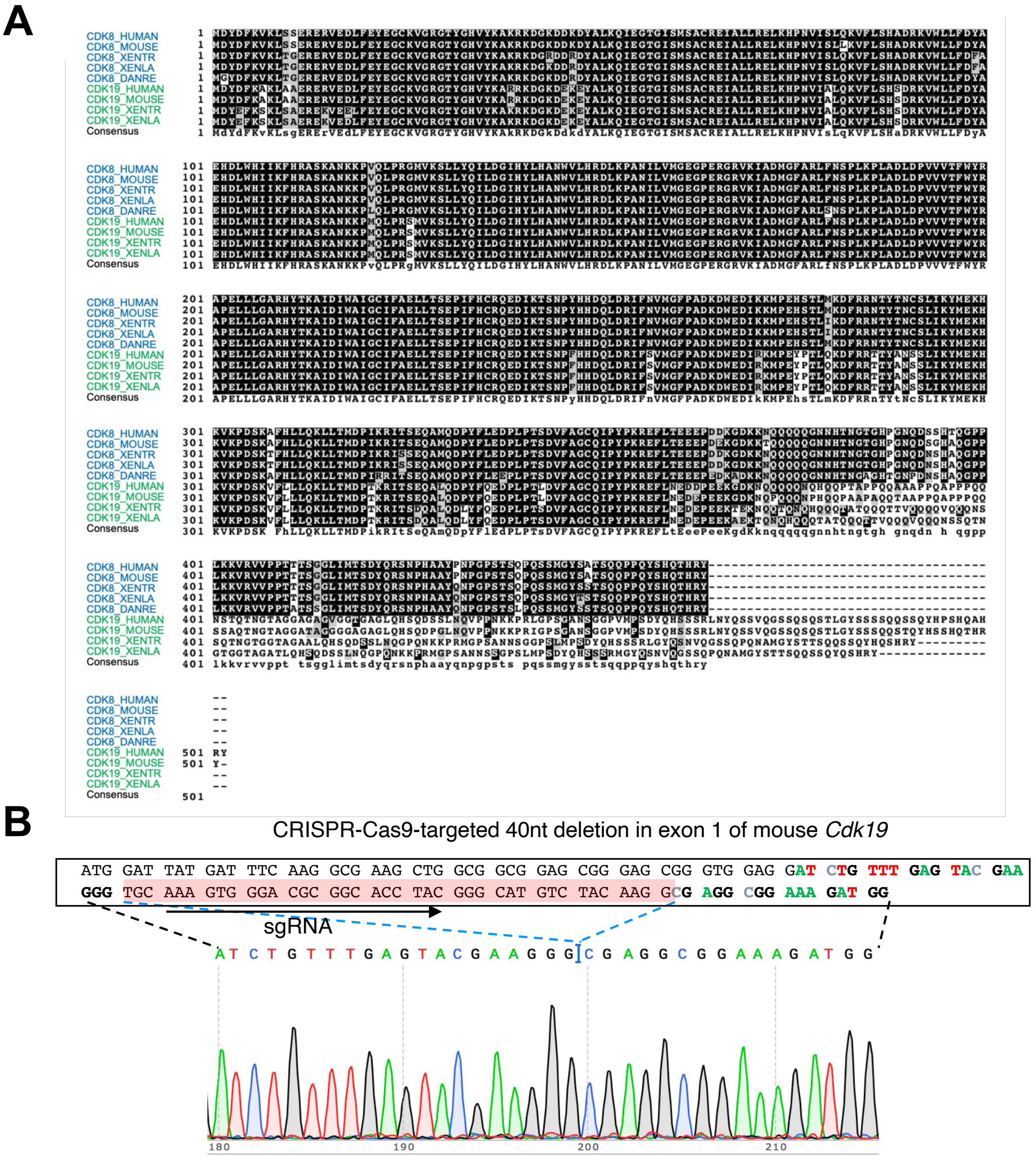

Appendix Fig S2. Amino acid sequence conservation of CDK8 and its paralogue CDK19 between different vertebrates.

A Sequence alignment of Cdk8 (blue) and Cdk19 (green) proteins from: *Homo sapiens, Mus musculus, Xenopus tropicalis, Xenopus laevis, and Danio rerio.* Homologous sequences are black. The consensus (> 80%) is presented below the alignment.

B Scheme indicating the fragment of *Cdk19* exon 1 removed by CRISPR-Cas9 (highlighted in red) in intestinal organoids. The arrow indicates the sequence of the sgRNA used. The sequence trace obtained after gene editing is presented below. The colour-code in the sequence in the box corresponds to the sequence trace.

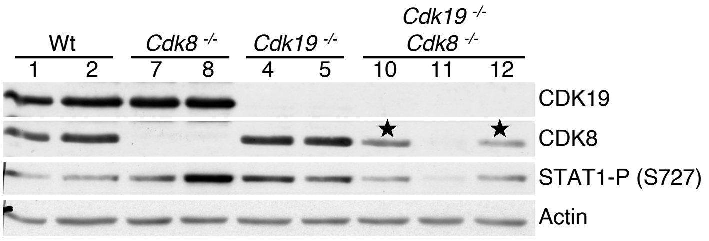

Appendix Fig S3. Cdk8/Cdk19 KO organoids are counter-selected.

WB indicating the levels of CDK8, CDK19 and phospho-STAT1-S727 in organoids after 14 days of OH-tamoxifen treatment. Two out of the three *Cdk8* $^{-/-}$/*Cdk19* $^{-/-}$ clones (numbers 10 and 12) show a reappearance of the CDK8 protein: compare with Fig. 2B where proteins were extracted from the same samples, but one week earlier. (★) indicates the two clones where CDK8 protein is detected; this was observed only in organoids where double KO had been induced.

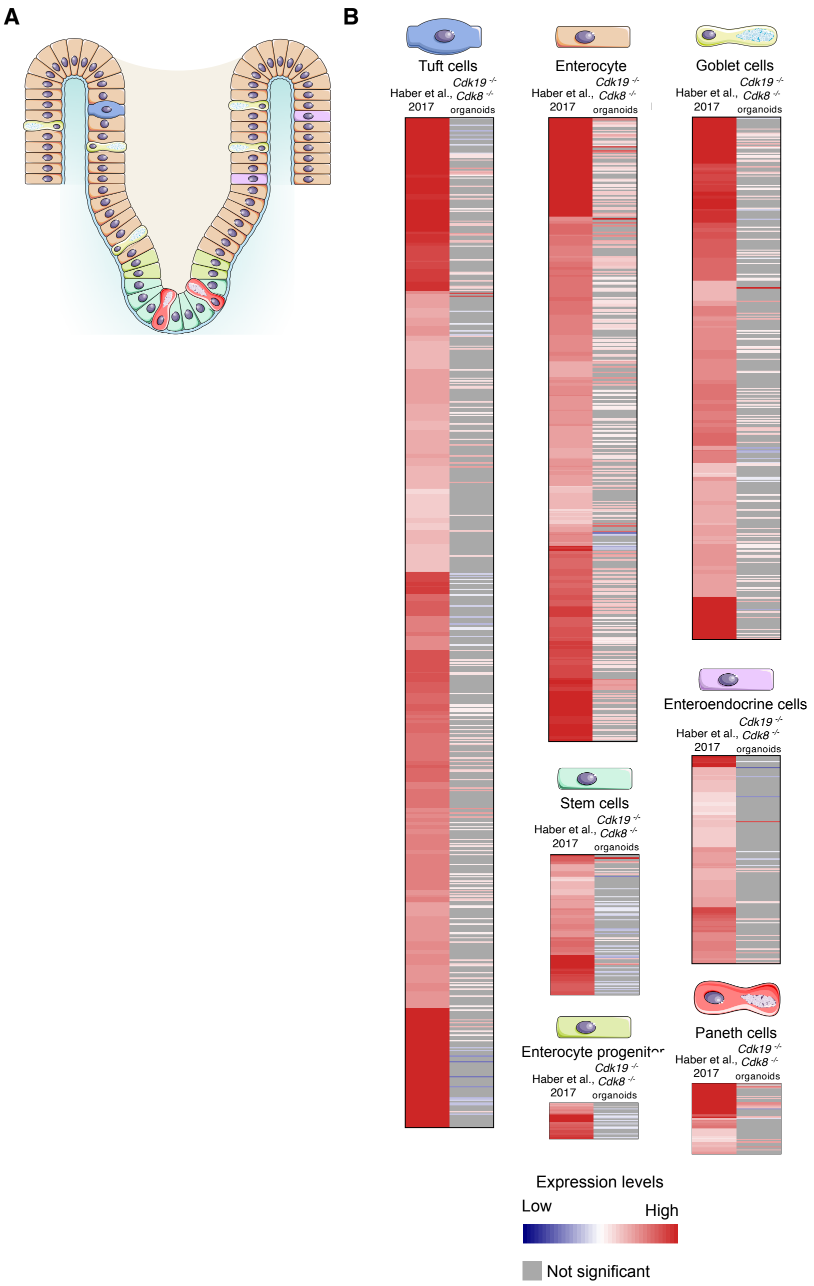

Appendix Fig S4. Cdk8/Cdk19 double knockout does not significantly affect intestinal cell differentiation.

A Schematic representation of the intestinal crypt and villus with all cell types indicated.
B Heatmaps visualising differences in gene expression in double *Cdk8/Cdk19* knockout organoids (bulk RNA-seq) and in indicated intestinal cell types obtained by single-cell RNAseq (Haber et al., 2017). After clustering and identifying the different cell types, gene signatures were defined as those genes in a given cell type that were significantly more expressed when compared against all other cell types, in a pairwise manner. Most signature genes for each cell type are not significantly deregulated (grey, right column of heatmaps) in organoids lacking CDK8 and CDK19.

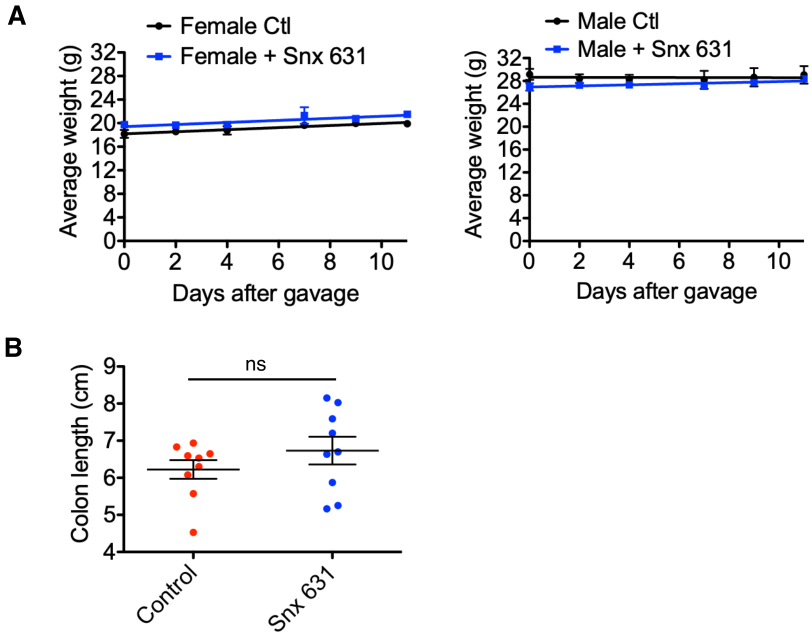

Appendix Fig S5. CDK8/19 inhibition is well tolerated by mice.

A, B Mice were treated with Senexin 631 (Snx 631, n=10) or vehicle (n=10) and mouse weight **(A)** during the experiment, and colon length **(B)** after 11 days of treatment were measured.
