# Supplementary material for: CDK8 and CDK19 act redundantly to control the CFTR pathway in the intestinal epithelium
Source: EMBO Rep. 2022 Dec 22;24(2):e54261. doi: 10.15252/embr.202154261 (PMC10549226; doi:10.15252/embr.202154261)
Supplement: Supplementary file 11 [file EMBR-24-e54261-s008.pdf]

EMBO reports

CDK8 and CDK19 act redundantly to control the CFTR pathway in the intestinal epithelium

Susana Prieto, Geronimo Dubra, Alain Camasses, Ana Bella Aznar, Christina Begon-Pescia, Elisabeth Simboeck, Nelly Pirot, François Gerbe, Lucie Angevin, Philippe Jay, Liliana Krasinska, and Daniel Fisher
DOI: 10.15252/embr.202154261

Corresponding author(s): Daniel Fisher (daniel.fisher@igmm.cnrs.fr)

Review Timeline:

Submission Date:	2nd Nov 21
Editorial Decision:	8th Nov 21
Revision Received:	11th Nov 21
Editorial Decision:	4th Feb 22
Revision Received:	15th Sep 22
Editorial Decision:	23rd Nov 22
Revision Received:	28th Nov 22
Accepted:	28th Nov 22

Editor: Esther Schnapp

Transaction Report:

(Note: With the exception of the correction of typographical or spelling errors that could be a source of ambiguity, letters and reports are not edited. Depending on transfer agreements, referee reports obtained elsewhere may or may not be included in this compilation. Referee reports are anonymous unless the Referee chooses to sign their reports.)

8th Nov 2021
Dear Dr. Fisher,

Thank you for the submission of your manuscript to EMBO reports. I have now read and discussed it with my colleagues here, and I am sorry to say that we all agree that it is not well suited for us.

We note that your study reports that CDK8 and CDK19 regulate the expression of CFTR pathway genes in mouse intestinal organoids. You show that they promote CFTR expression and repress genes with a role in mucus production. We think that these are interesting observations. However, we also note that it remains unclear how CDK8 and CDK19 regulate CFTR pathway genes, and whether CDK8 and CDK19 are linked to cystic fibrosis in vivo. It was also reported before that CDK8 and CDK19 regulate the expression of specific gene sets, versus a more global role in transcription, as you mention. We think that the manuscript is not sufficiently developed for consideration for publication here, and we have therefore decided not to proceed with in-depth review.

That said, we think that your work is an excellent candidate for our partner journal Life Science Alliance (http://www.life-science-alliance.org/; our broad scope Open Access journal published in partnership between the EMBO-, Rockefeller University-, and Cold Spring Harbor Laboratory Presses). Eric Sawey, Executive Editor of Life Science Alliance (e.sawey@life-science-alliance.org) would be pleased to send your manuscript for in-depth peer review; no reformatting is required. We very much hope that you will be interested in this option: please follow the link below for transfer.

For EMBO reports, I am sorry that I cannot be more positive this time, and I thank you once more for your interest in our journal.

Yours sincerely,

Esther Schnapp, PhD
Senior Editor
EMBO reports

** As a service to authors, EMBO Press provides authors with the ability to transfer a manuscript that one journal cannot offer to publish to another journal, without the author having to upload the manuscript data again. To transfer your manuscript to another EMBO Press journal using this service, please click on Link Not Available

Dear editors,

Please find enclosed an original research article entitled "*CDK8 and CDK19 act redundantly to control the CFTR pathway in the intestinal epithelium*", which we wish to submit to EMBO Reports. CDK8 and CDK19 are the only two members of the most highly conserved CDK subfamily in vertebrates (and their cognate cyclin, cyclin C, is also the most highly conserved vertebrate cyclin), suggesting that they have essential functions. They form part of the cyclin-dependent kinase module (CKM) of the Mediator transcriptional co-regulator complex, a complex that is essential for all RNA polymerase II-dependent gene expression. The CKM is generally considered a negative regulator that sterically prevents Mediator binding to the basal transcriptional machinery. Yet CDK8 and CDK19 have also been found to positively regulate gene expression, and are each thought to direct expression of different genes. Several mechanisms of action have been proposed for CDK8, and involve phosphorylation of either specific transcription factors or the C-terminal domain (CTD) of RNA polII at specific genes during transcriptional activation, although how specificity is achieved has not been explained. To date, it appears that most cell types can survive in the absence of either CDK8 or CDK19, whose individual depletion or gene disruption has only limited effects on gene expression.

Given their extremely high structural identity and their binding to a unique cyclin, it is surprising that CDK8 and CDK19 have not been found to be functionally redundant, and whether or not cells retain an ability to activate transcriptional programmes, or even survive, in the absence of both kinases is not known. Here, we first confirm a previous report that CDK8 is not required for intestinal homeostasis or tumourigenesis in the mouse, despite still being widely thought of as a colorectal cancer oncogene. We further show that CDK8 is not required *in vivo* to phosphorylate RNA polII CTD in the absence of the main CTD kinase, CDK7. Next, using gene targeting in mice and intestinal organoids, we demonstrate that the combined deletion of CDK8 and CDK19 does not prevent cell proliferation and differentiation, but they act redundantly to control tissue-specific gene expression. Finally, we show that CDK8/19 are required in the intestinal epithelium for expression and function of the cystic fibrosis transmembrane conductance regulator (CFTR) and associated mucin genes, whose regulation is a poorly understood aspect of epithelial homeostasis. This depends on their kinase activity and can be modulated by pharmacological inhibition. We conclude that expression of the Mediator kinases is not essential for cell proliferation and differentiation, but they act redundantly to regulate tissue-specific transcriptional programmes, including the CFTR pathway in the intestinal epithelium. Given that CDK8/19 inhibitors are being considered as potential therapeutic agents in cancer, these novel findings may be important. We believe that our results should be of major interest to scientists working on transcriptional regulation and on cancer, and deserve widespread exposure in a highly visible general biology journal such as EMBO Reports.

Yours faithfully,

Daniel Fisher, DPhil

2nd Editorial Decision 4th Feb 2022

Dear Daniel,

Thank you for the submission of your manuscript to EMBO reports, and for your proposed point-by-point response. I went through it, and I think that the revisions you propose are reasonable.

I would thus like to invite you to revise your manuscript with the understanding that the referee concerns must be fully addressed and their suggestions taken on board. Please address all referee concerns in a complete point-by-point response. Acceptance of the manuscript will depend on a positive outcome of a second round of review. It is EMBO reports policy to allow a single round of major revision only and acceptance or rejection of the manuscript will therefore depend on the completeness of your responses included in the next, final version of the manuscript.

We realize that it is difficult to revise to a specific deadline. In the interest of protecting the conceptual advance provided by the work, we recommend a revision within 3 months (7th May 2022). Please discuss the revision progress ahead of this time with me or the editorial office if you require more time to complete the revisions.

You can either publish the study as a short report or as a full article. For short reports, the revised manuscript should not exceed 27,000 characters (including spaces but excluding materials & methods and references) and 5 main plus 5 expanded view figures. The results and discussion sections must further be combined, which will help to shorten the manuscript text by eliminating some redundancy that is inevitable when discussing the same experiments twice. For a normal article there are no length limitations, but it should have more than 5 main figures and the results and discussion sections must be separate. In both cases, the entire materials and methods must be included in the main manuscript file.

IMPORTANT NOTE: we perform an initial quality control of all revised manuscripts before re-review. Your manuscript will FAIL this control and the handling will be DELAYED if the following APPLIES:
1) A data availability section providing access to data deposited in public databases is missing. If you have not deposited any data, please add a sentence to the data availability section that explains that.
2) Your manuscript contains statistics and error bars based on n=2. Please use scatter blots in these cases. No statistics should be calculated if n=2.

When submitting your revised manuscript, please carefully review the instructions that follow below. Failure to include requested items will delay the evaluation of your revision.

1) a .docx formatted version of the manuscript text (including legends for main figures, EV figures and tables). Please make sure that the changes are highlighted to be clearly visible.

2) individual production quality figure files as .eps, .tif, .jpg (one file per figure). See https://wol-prod-cdn.literatumonline.com/pb-assets/embo-site/EMBOPress_Figure_Guidelines_061115-1561436025777.pdf for more info on how to prepare your figures.

3) We replaced Supplementary Information with Expanded View (EV) Figures and Tables that are collapsible/expandable online. A maximum of 5 EV Figures can be typeset. EV Figures should be cited as 'Figure EV1, Figure EV2" etc... in the text and their respective legends should be included in the main text after the legends of regular figures.

- For the figures that you do NOT wish to display as Expanded View figures, they should be bundled together with their legends in a single PDF file called *Appendix*, which should start with a short Table of Content. Appendix figures should be referred to in the main text as: "Appendix Figure S1, Appendix Figure S2" etc. See detailed instructions regarding expanded view here: <https://www.embopress.org/page/journal/14693178/authorguide#expandedview>

- Additional Tables/Datasets should be labeled and referred to as Table EV1, Dataset EV1, etc. Legends have to be provided in a separate tab in case of .xls files. Alternatively, the legend can be supplied as a separate text file (README) and zipped together with the Table/Dataset file.

4) a .docx formatted letter INCLUDING the reviewers' reports and your detailed point-by-point responses to their comments. As part of the EMBO Press transparent editorial process, the point-by-point response is part of the Review Process File (RPF), which will be published alongside your paper.

5) a complete author checklist, which you can download from our author guidelines <https://www.embopress.org/page/journal/14693178/authorguide>. Please insert information in the checklist that is also reflected in the manuscript. The completed author checklist will also be part of the RPF.

6) Please note that all corresponding authors are required to supply an ORCID ID for their name upon submission of a revised manuscript (<https://orcid.org/>). Please find instructions on how to link your ORCID ID to your account in our manuscript tracking system in our Author guidelines

<https://www.embopress.org/page/journal/14693178/authorguide#authorshipguidelines>

7) Before submitting your revision, primary datasets produced in this study need to be deposited in an appropriate public database (see https://www.embopress.org/page/journal/14693178/authorguide#datadeposition). Please remember to provide a reviewer password if the datasets are not yet public. The accession numbers and database should be listed in a formal "Data Availability" section placed after Materials & Method (see also https://www.embopress.org/page/journal/14693178/authorguide#datadeposition). Please note that the Data Availability Section is restricted to new primary data that are part of this study. * Note - All links should resolve to a page where the data can be accessed. *
If your study has not produced novel datasets, please mention this fact in the Data Availability Section.

8) We would also encourage you to include the source data for figure panels that show essential data. Numerical data should be provided as individual .xls or .csv files (including a tab describing the data). For blots or microscopy, uncropped images should be submitted (using a zip archive if multiple images need to be supplied for one panel). Additional information on source data and instruction on how to label the files are available at <https://www.embopress.org/page/journal/14693178/authorguide#sourcedata>.

9) Our journal also encourages inclusion of *data citations in the reference list* to directly cite datasets that were re-used and obtained from public databases. Data citations in the article text are distinct from normal bibliographical citations and should directly link to the database records from which the data can be accessed. In the main text, data citations are formatted as follows: "Data ref: Smith et al, 2001" or "Data ref: NCBI Sequence Read Archive PRJNA342805, 2017". In the Reference list, data citations must be labeled with "[DATASET]". A data reference must provide the database name, accession number/identifiers and a resolvable link to the landing page from which the data can be accessed at the end of the reference. Further instructions are available at https://www.embopress.org/page/journal/14693178/authorguide#referencesformat

10) Regarding data quantification (see Figure Legends: https://www.embopress.org/page/journal/14693178/authorguide#figureformat)

The following points must be specified in each figure legend:

- the name of the statistical test used to generate error bars and P values,

- the number (n) of independent experiments (please specify technical or biological replicates) underlying each data point,

- the nature of the bars and error bars (s.d., s.e.m.),

- If the data are obtained from n {less than or equal to} 2, use scatter blots showing the individual data points.

Discussion of statistical methodology can be reported in the materials and methods section, but figure legends should contain a basic description of n, P and the test applied.

- Please also include scale bars in all microscopy images.

11) The journal requires a statement specifying whether or not authors have competing interests (defined as all potential or actual interests that could be perceived to influence the presentation or interpretation of an article). In case of competing interests, this must be specified in your disclosure statement. Further information: https://www.embopress.org/competing-interests

We would also welcome the submission of cover suggestions, or motifs to be used by our Graphics Illustrator in designing a cover.

As part of the EMBO publication's Transparent Editorial Process, EMBO reports publishes online a Review Process File (RPF) to accompany accepted manuscripts. This File will be published in conjunction with your paper and will include the referee reports, your point-by-point response and all pertinent correspondence relating to the manuscript.

You are able to opt out of this by letting the editorial office know (emboreports@embo.org). If you do opt out, the Review Process File link will point to the following statement: "No Review Process File is available with this article, as the authors have chosen not to make the review process public in this case."

I look forward to seeing a revised form of your manuscript when it is ready. Please use this link to submit your revision: https://embor.msubmit.net/cgi-bin/main.plex

Kind regards,
Esther

Esther Schnapp, PhD
Senior Editor
EMBO reports

Referee #1:

This manuscript describes the phenotype of Cdk8-deficient intestines as well as the combination of Cdk8 and Cd19 knockout in intestinal organoids. Genetic ablation of Cdk8 results in no differences in cell proliferation of the distribution of the different cell types in the intestine. In addition, no effect of Cdk8 deletion is observed during colitis-induced weight loss or during the generation of intestinal tumors. To study Cdk 8 and Cdk19 in parallel, the authors generate organoids from Cdk8(lox/lox) intestines and combine tamoxifen-derivatives with CRISPR/Cas9 mediated excision of Cdk19. In this case, this double treatment shows some mild effects in proliferation or differentiation as well as altered transcription of CFTR-dependent genes.

Overall, the idea of combining Cdk8 and Cdk19 is quite interesting but I am afraid that the manuscript is quite preliminary for generating solid conclusions. In particular, the characterization of mutant organoids is quite basic. The links between Cdk8/19 and CFTR are also not explored. Finally, I am not sure that the protocols for Cdk8 ablation in vivo were effective using the strategy described in the manuscript.

1. The correlation of Cdk8/19 knockout with deregulated CFTR-controlled genes is interesting but it is difficult at the moment to establish any causal conclusions. CFTR is involved in many processes during development and this correlation may simply be caused by, e.g., defective differentiation. A correlative ChIP-seq analysis in parallel with CFTR would be very informative... Biochemical investigation of Cdk8 and Cdk19 complexes couls also be useful.

2. It is not clear whether the defects in Cdk8-Cdk19-deficient organoids are due to decreased proliferation or abnormal differentiation of different lineages. The authors may somehow have this information in the RNAseq experiments, although bulk RNAseq may be confusing to estimate defects in specific lineages. A minimal characterization of the major cell types by IF may also be informative.

3. Protocols for Cdk8 depletion in vivo. It seems that Cre was induced by a single IP injection of tamoxifen + 5 days with tamoxifen diet. Why this diet was not continued for more days? In other models, it is not unusual to keep mice on tamoxifen for months. A single injection + 5 days on diet are very likely to be insufficient for promoting excision of Cdk8 in many cells (e.g. quiescent progenitors, etc.)

4. The problem indicated above may be especially important in AOM/DSS induced pathologies. Mice were treated with tamoxifen as indicated above and treated later with the carcinogens. I don't understand this protocol. Carcinogen treatment may activate cells (e.g quiescent progenitors that are still WT for Cdk8. Why tamoxifen was not given (also) after carcinogen treatment. I am afraid that the way these protocols were performed may be hiding any potential effect of Cdk8 knockout.

Other points
Fig. 2D. are the differences between double KO and single KOs or WT significant at day 6?

Methods: "Cdk8 lox/lox mice were crossed with Villin-Cre-ERT2 +/- mice to obtain Cdk8 lox/lox, Villin-Cre-ERT2 +/-" seems a too obvious sentence.

Cdk19 CRISPR knockout seems to be at least partially repeated in two sections (Organoids and CRISPR)

Referee #2:

In recent years, enhancer-promoter interactions have received significant attention as they underlie 3D chromatin architecture and the transcriptional program of cell identity. Transducing information from enhancers to the correct promoters, the Mediator complex (containing the analogue kinases CDK8/19) is pivotal for many processes and defining cell identity. This manuscript addresses several outstanding questions in the field of transcriptional CDKs, and more widely, relating enhancers, Mediator, and the core transcriptional machinery to cancer susceptibility and cystic fibrosis.

Here, Prieto and colleagues have investigated the role of the kinases CDK8 and CDK19 by conditional deletion of one or both genes in the mouse intestine, and in derived intestinal organoids. They have examined the effect of CDK8/19 deletion on the phosphorylation of RNA Pol II (a reported target of CDK8 activity, although in early papers, in vitro), global gene expression, cell proliferation, tumorigenesis, and differentiation capacity. Their data reveals the extent to which these sister kinases have

independent or redundant functions, an under-explored aspect of Mediator function, since only one may occupy the Mediator complex at a time. Moreover, given the central position of Mediator in the enhancer-promoter interaction, and the fact that the CDK8/19 kinases represent the only enzymatic subunits of Mediator, Prieto and colleagues report that double-deletion of these kinases has a surprisingly limited effect on intestinal organoid differentiation capacity and tumorigenesis. The Authors then use their intestinal organoid system to identify a link between CDK8/19 and cystic fibrosis, specifically the CFTR pathway.

The methods and experiments using tissue-specific knockout of CDK8/19 in vivo and in intestinal organoids in vitro, appear robust. The new findings of Prieto et al will surprise many readers, as they reveal that, in some contexts, CDK8 and CDK19 are not essential, unlike general transcriptional machinery. Importantly, the Authors combine genetic and chemical methods to convincingly dissect the individual vs combined functions of the sister kinases CDK8 and CDK19, as well as separately investigating their kinase activity, important nuances which are nevertheless rare in the field. Lastly, the studies using CDK8/19 conditional deletion in organoids leads to a novel link between CDK8/19 function and Cystic Fibrosis.

As a result, overall, the manuscript is likely to be of interest to a wide audience since it explores new and important avenues on the role of CDK8/19 and Mediator in cancer, development and cystic fibrosis. I thank the Authors for an interesting read. Below I outline some Major and Minor comments:

Major Comments

Cystic Fibrosis (CF) patients lose CFTR function and are highly susceptible to early, aggressive colorectal tumor development. Similarly, based on the Authors data, a CDK8/19 small molecule inhibitor might be expected to reduce CFTR expression in the intestine in vivo. This would have wider implications for many readers, concerning the concept of using CDK8/19i in vivo in future clinical applications. The manuscript would benefit if the Authors examine whether the CFTR becomes reduced in vivo in the intestine, in mice treated with CDK8/19i, or a CF-like phenotype in mouse intestine treated with a CDK8/19-inhibitor. In any case, the Authors should comment on this possibility given its wider implications for the use of CDK8/19 inhibitors in humans.

Main Fig1, Fig2, and Fig4 are very clear.

Fig3: The Authors describe in the Results section that CDK8-KO intestinal epithelium shows almost no transcriptomic changes versus wild-type intestine (DEG = 4 genes). In contrast, CDK8-KO intestinal epithelium organoids show 716 up, 575 down, total DEGs = 1291 genes. Even with a 2x threshold, DEG's = 390 genes (Figure 3A). Can the Authors comment on this difference? Moreover, in the Discussion section, the Authors make a conclusion that needs clarification or qualification, as it appears at odds with the data: "Our results also do not support an essential role for Mediator kinases in general gene expression, in contrast to Mediator itself, since relatively few genes were highly deregulated in double knockouts." However, this only appears to apply to the in vivo intestinal tissue for CDK8-KO, but not the organoids for CDK8-KO or CDK8/19-dKO -can the Authors include this nuance in the Discussion, and comment on reasons for this large difference? In particular, the data does show a very large effect on gene expression for CDK8/19-double KO, where it appears > 3600 differential genes were observed (Figure 3B). Therefore the current statement seems out of place, and may require re-phrasing.

Can the Authors clarify if their RNAseq expts with a "spike-in" to detect a global shift in the whole transcriptome? (This is not mentioned in the Methods) RNAseq has an internal normalization that can hide a global shift in the whole transcriptome (Loven J et al., 2012, Cell, PMID: 23101621). This might apply in a situation like this manuscript where the core transcriptional machinery is being manipulated. It is perfectly acceptable if no spike-in was included, not everyone needs to do this, however it might offer an explanation for why few genes might be observed as differential in the case of the intestinal epithelium.

The Authors mention in their Discussion section: "we found that there was a strong overlap between transcriptome changes of double knockout organoids and intestinal knockout of the gene encoding CFTR...." However I did not find the Figure reference for the comparison of transcriptomic changes in CDK8/19i vs CFTR-KO, as mentioned -can the Authors please clarify this, or show this analysis?

Minor Comments

Introduction: the statement: "CDK19 deletion has not yet been reported." I suggest to specify that this applies in vivo, highlighting the novelty of the new study. In vitro, CDK19 has been knocked out in a number of cell types.

In this sentence in the Results section, I think the word "as" might be missing. Otherwise it is not clear: "We found that Senexin B treatment for 24h leads to the downregulation of Cftr and upregulation of Muc3 expression (Fig 4G) "AS" seen upon genetic ablation of both Cdk8 and Cdk19, indicating that kinase activity of CDK8/19 controls CFTR pathway gene expression."

In this sentence in the Results section, I think the word "they" refers to "CDK8/19", but it could alternatively refer to "quiescent cells" -I suggest to re-phrase to clarify: "....(Koehler et al, 2016) our data suggest that in the intestinal epithelium, cells devoid of both CDK8 and CDK19 have an increased tendency to become quiescent, implying that they provide a growth advantage."

Referee #3:

Using conditional "floxed" KO mouse models and small intestinal (SI) organoids generated from these mice or gene edited by CRISPR/Cas9 to obtain conditional double KOs for the cyclin-dependent kinases CDK8 and CDK19, this study shows rather convincing evidence for a functional redundancy of these highly conserved kinases in the control of gene expression by the essential Mediator gene transcription complex. However, the combined deletion or pharmacological inhibition of CDK8 and CDK19 in intestinal organoids is not lethal but reduces proliferation, results in loss of Cyclin C, and induces a cystic fibrosis-like phenotype, characterized by a partial loss of CFTR expression and function, and mucus (Muc2 and 3) accumulation and hypersecretion by goblet cells. The models used and the findings obtained in this study are robustly documented and are new and of considerable scientific and medical interest in the area of tumor biology and CF. A major limitation is however its focus on a single species (adult mice, not humans) and tissue (small intestine). Yet there are several points of criticism that require further attention.
Specific comments:
1. On p. 4, 13 l. up the authors justify the use of Cdk8/Cdk19 double KO intestinal organoids rather than mice as a model by claiming that Cdk8/Cdk19 double knockout in adult mice is most probably lethal. However it is unclear why they did not attempt to generate Cdk8lox/lox/Cdk19lox/lox mice and to cross them with a Tamoxifen-inducible Cre expressed under control of the intestine-specific villin promoter. Assuming that the double KO would slow down intestinal cell proliferation but is not lethal and allows differentiation, as reported for the organoids, Tamoxifen treatment is not expected to induce a severe morbidity or lethality at the intestinal level on the short run. Because the intestinal phenotype of CF mice has been described in much detail in the literature (e.g. see Kreda et al Cold Spring Harb Perspect med 2012; 2:a009589; Ikpa et al Genomics 2020; 112: 1139), confirmation of the CF-like phenotype in double Cdk8/Cdk19 knockout mice in vivo rather than in organoids would further strengthen this study.
2. CDK8/19 deletion or Senexin B inhibition of the kinase activities in the SI organoids resulted in the gradual loss of CFTR expression, CFTR protein, and CFTR function, as measured by qRT-PCR, WB, and the organoid swelling (FIS) assay, respectively (Fig. 3, D,E; Fig. 4, E-G). However, these observations raise several questions: (i) (WT) CFTR transcripts are down by only ~25% in Cdk8 KO organoids and by ~50% in the double KOs (Fig. 3D). It is well known that a loss of 50% WT CFTR expression and activity as seen in CF carriers (CFTR+/severe mutation) does not cause any pathology and is beyond the dynamic window of the FIS assay. It is more likely, therefore, that CDK8/19 deletion results in a much more dramatic loss of expression and function of other transporters or regulators required to allow the forskolin-activated, CFTR-dependent anion secretion measured by FIS, e.g. NKCC1, NBCe1, adenylyl cyclase or PK-A (please note that anion and fluid secretion in 3D organoids as monitored by FIS is also dependent on Cl- or HCO3- import across the basolateral membrane of the enterocytes and on AC and PKA activity). Therefore the authors need to verify in their RNA-Seq data base (Fig, 3B) whether one or more of these genes are strongly downregulated in the double KO mice; (iii) Western blotting or ICH of rodent CFTR is notoriously difficult to perform because of a lack of specific monoclonal antibodies, and is at best semi-quantitative. It is doubtful whether the band stained by the monoclonal CFTR Ab2784 in Fig. 3E represents the C-band of complex-glycosylated CFTR, which normally is broad and very diffuse. Therefore the whole blot rather than a small section of the blot should be made accessible to the reviewer, and the identity of the band and the specificity of the antibody for mouse CFTR should be verified by WB of a Cftr-/- mouse. Also the number of blots examined should be given. Considering the 1363 genes that are downregulated in the double KO organoids (Fig. 3B), there are multiple candidate proteins that could possibly crossreact with the Abcam antibody; (iv) a more direct assessment of CFTR activity in intestinal organoids can be achieved by electrical current measurements across monolayers of organoids permeabilized basolaterally by a pore-forming agent, e.g. nystatin.
3. Strong downregulation of CFTR and upregulation of Muc2 in goblet cells is also seen upon differentiation of WT intestinal organoids by removing Wnt3a from the culture medium and/or by inhibiting Notch signaling by the -secretase inhibitor DAPT. Would it be possible, in analogy, that the KO of CDK8+19, or the inhibition of cyclin-dependent kinase activity by Senexin B results in the repression of genes in the Notch signaling pathway, explaining the partial inhibition of cell proliferation (Fig. 2F) and the conversion into a mucoid phenotype? Did pathway analysis of the RNA-Seq data showed downregulation of the Notch signaling components?
4. Fig. 3B: How do the authors explain that in the Venn diagram only 27 and 14 out of the 155 respectively 151 genes that are up-or downregulated in the Cdk19-/- organoids are also up-or downregulated in the double KO organoids? How can the additional knockdown of Cdk8 result in the loss of up-and downregulation of 128 respectively 137 genes?
5. Fig. 4F: The extent of FIS inhibition in the Cdk8-/-/Cdk19-/- mice (~80% of WT) was much higher than the inhibition by saturating concentrations (10 uM) of SenB (<40% of WT), suggesting that the loss of CDK proteins has more impact on CFTR function than the loss of CDK kinase activity. Therefore the claim that "the kinase activity of CDK8/19 controls CFTR pathway expression" (p. 5, 9 l. up) should be toned down.
6. Fig. 4G: MUC2, not MUC3 is the major secreted mucin in mouse intestine. Was the expression of the Muc2 gene also upregulated by SenB?
7. To allow a fair comparison, was the genetic background of the WT mice or intestinal organoids always identical to the one of the KO mice/organoids?
8. In the Method section, the description of the CRISPR/Cas9 editing of the organoids on p. 8, ala. 3 is highly repetitive with the text on p. 13, ala. 2. Please correct this duplication.
9. Some minor typo's should be corrected, e.g. p. 13, l. 6: deferentially=differentially; p. 19, 4 l. up: umpaired=unpaired

Reply to editor and reviewer 1

Dear Esther,
Please find enclosed a revised version of our article "CDK8 and CDK19 control the CFTR pathway in the intestinal epithelium", by Prieto et al (n°: EMBOR-2021-54261V3), whose title we have changed to the more specific "CDK8 and CDK19 act redundantly to control the CFTR pathway in the intestinal epithelium". We apologise for the time this revision has taken, but we preferred to thoroughly respond to all of the reviewers' comments. This involved new mouse experiments, and has taken a fair amount longer than the originally suggested two months. Please find below our detailed responses to the reviewers' criticisms, explaining what we have done to satisfy their queries (reviewers' comments in blue, our reply in black). For completeness, in parts we have reiterated our original reply to the reviewers (as we do not know whether this was already communicated to the reviewers).

Reviewer 1
This manuscript describes the phenotype of Cdk8-deficient intestines as well as the combination of Cdk8 and Cdk19 knockout in intestinal organoids. Genetic ablation of Cdk8 results in no differences in cell proliferation of the distribution of the different cell types in the intestine. In addition, no effect of Cdk8 deletion is observed during colitis-induced weight loss or during the generation of intestinal tumors. To study Cdk8 and Cdk19 in parallel, the authors generate organoids from Cdk8(lox/lox) intestines and combine tamoxifen-derivatives with CRISPR/Cas9 mediated excision of Cdk19. In this case, this double treatment shows some mild effects in proliferation or differentiation as well as altered transcription of CFTR-dependent genes.

Overall, the idea of combining Cdk8 and Cdk19 is quite interesting but I am afraid that the manuscript is quite preliminary for generating solid conclusions.

We understand that the control of secretion pathways by Mediator kinases is a very new finding and therefore might appear "preliminary", and we have reinforced this with new experiments. We cannot, however, agree that the manuscript overall is preliminary, and the other reviewers do not appear to feel this is the case either. Our study involved ten years of work to generate the mouse models and to extensively explore the resulting phenotypes. We have improved our description of the context so that our findings can be more easily appreciated. It is worth pointing out that there is a lot of material published in high impact journals about the role of CDK8 as an oncogene or a tumour suppressor (cf Firestein et al., 2008, Nature; Morris et al., 2008, Nature; Kapoor et al., 2010, Nature; Pelish et al., 2015, Nature, to cite but a few). Yet the evidence for either is not particularly strong, and the first CDK8 conditional knockout in the mouse (from the Firestein lab, McCleland et al, 2015, J. Pathol) found no evidence for the oncogenic activity that they first reported in 2008 (incidentally, inadvertently scooping us as we had similar results). This was extremely unexpected; indeed, CDK8 was thought to be essential as its constitutive knockout is lethal at the pre-implantation embryonic stage (Westerling et al. 2007). However, in our conditional CDK8 knockout mouse model, in contrast to the Firestein lab, we did not find any evidence for tumour suppressor activity. While a "negative" result, this will nevertheless have a certain impact. We next tested for functional redundancy with the paralogue, CDK19. Loss of both kinases was widely expected to be lethal. Our findings that it is not, that differentiation in a complex tissue can still occur in their absence, and that transcriptional effects are also quite limited, while perhaps not spectacular, are reliable and important results. Finally, in this revised version, we have performed new experiments that strengthen the conclusions and we hope will convince this reviewer that our study is not as preliminary as it initially might have seemed.

In particular, the characterization of mutant organoids is quite basic. The links between Cdk8/19 and CFTR are also not explored.

We understand and accept this criticism, and we have now provided a better characterisation of mutant organoids, as described below. As for the links between Mediator kinases and CFTR, the reviewer should be aware that no papers on CDK8/19 to date have provided a convincing mechanism relating to their specificity for certain genes. In general, either ChIP-seq of CDK8 itself is performed, showing that CDK8 binds promoters of certain genes – but why these genes and not

others is not explained; and/or S2/S5 phosphorylation of the C-Terminal Domain of RNA Polymerase II is performed, showing that on the genes that are downregulated, Pol II CTD phosphorylation is reduced, which is expected for a downregulated gene and again tells us nothing new about why that gene and not others is a target of CDK8/19. Nevertheless, we have done more to address the reasons for the CF-like phenotype in the absence of these kinases since, as other reviewers also point out, it may not all be about CFTR itself, and this has turned out to be the most likely explanation of some of the results.

Finally, I am not sure that the protocols for Cdk8 ablation in vivo were effective using the strategy described in the manuscript.

As pointed out by other reviewers in the cross-refereeing comments, this is not an issue as we have demonstrated the effectiveness of the strategy used for *Cdk8* ablation *in vivo* in original figures by IHC (Fig 1 and Fig EV5), PCR-genotyping (Fig EV1) and western blot (Fig EV2).

1. The correlation of Cdk8/19 knockout with deregulated CFTR-controlled genes is interesting but it is difficult at the moment to establish any causal conclusions. CFTR is involved in many processes during development and this correlation may simply be caused by, e.g., defective differentiation. A correlative ChIP-seq analysis in parallel with CFTR would be very informative... Biochemical investigation of Cdk8 and Cdk19 complexes couls also be useful.

We agree with this statement but, as pointed out above, even if we found by ChIP-seq that CDK8 is directly bound to the promoter of CFTR, this would not explain why the CFTR promoter recruits CDK8, nor how the regulation works. It should also be stated that ChIP-seq from intestinal organoids would be extremely technically challenging and would require a large amount of organoid material and a long-time to optimise, even assuming that the available antibodies for CDK8 were suitable, which is far from certain. In the only two published CDK8 ChIP-seq, from 1) the Espinosa lab (Galbraith et al., Cell, 2013), which was performed in a cancer cell line, HCT-116; 2) the Taatjes and Shair study (Pelish et al., Nature, 2015), which was performed in an AML cell line, MOLM-14, neither were controlled for CDK8 specificity by downregulating CDK8. Nevertheless, this data is publicly available on GEO and we have interrogated it to see if there is evidence for CDK8 binding to the CFTR promoter. In summary, there is no convincing evidence for this: see figure below, in which we show the data from CDK8 ChIP-seq in HCT-116 cells for the CFTR gene and the positive control gene c-Myc. However, there are many technical reasons why direct binding of CDK8 to the CFTR promote might not be seen by ChIP-seq, and it also might be different in intestinal tissue compared with cancer cell lines, but the reduction of CFTR expression in CDK8/19 knockout may well also be indirect.

Figure showing CDK8 ChiP-Seq for CFTR gene (top), where no peaks were called; c-Myc (below), showing clear peaks.

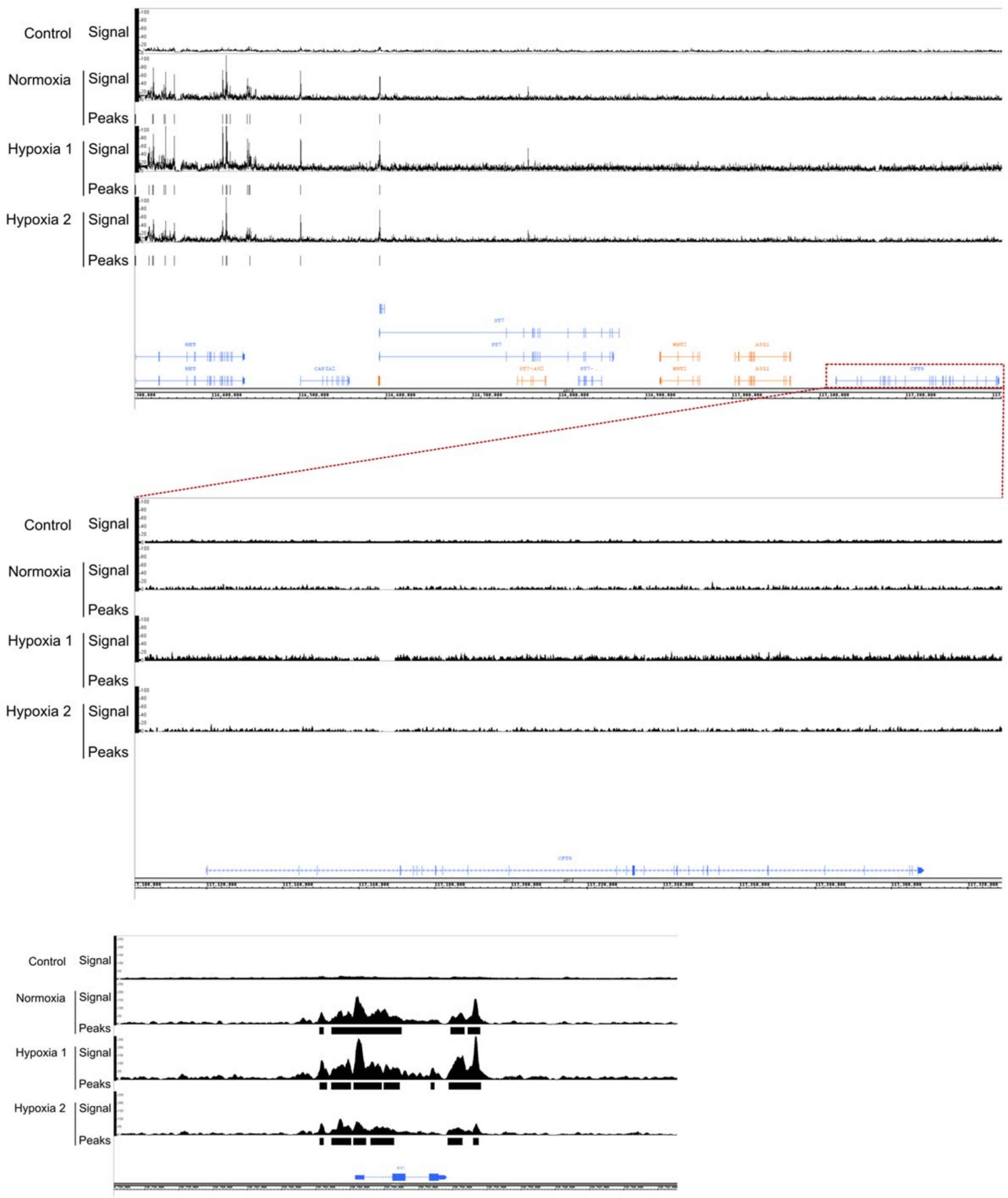

Finally, we find the suggestion '' biochemical investigation of Cdk8 and Cdk19 complexes could also be useful", while certainly true, a bit vague, and unrealistic to perform as part of this study – it would require a study in itself.

2. It is not clear whether the defects in Cdk8-Cdk19-deficient organoids are due to decreased proliferation or abnormal differentiation of different lineages. The authors may somehow have this information in the RNAseq experiments, although bulk RNAseq may be confusing to estimate defects in specific lineages. A minimal characterization of the major cell types by IF may also be informative.

Reply to reviewer 1

This is a good suggestion. We have investigated whether there is a skewed differentiation in double Cdk8/19 knockouts, as now described in new Fig 3 and new Fig EV9. First, we performed IHC experiments on particular markers for different cell types in WT and double *Cdk8/19* knockouts. Muc2 staining (goblet cells) was higher in knockouts, while staining for proteins specific for stem cells (the luminally secreted Olfm4), Paneth cells (lysozyme) and tuft cells (Dlck1) was not altered (new Fig 3C).
Next, we interrogated our RNA-seq data, as also suggested by other reviewers. New Fig EV9 correlates our RNA-seq data with published single cell RNA-seq data for different intestinal cell types. Again, globally, there is no obvious bias in markers for different cell types, but there is a decrease of a subset of stem cell marker genes and an increase of a subset of enterocyte-specific genes. Finally, in Fig 3D, we analysed expression of markers for different cell lineages by qRT-PCR in WT and double knockout organoids. This confirmed that there is no systematic increase or decrease of all markers of any particular lineage, and no changes for any gene of more than about two-fold (with the exception of Notch1, downregulated about 4-fold). There are, nevertheless, slight but reproducible changes, for example, a decrease in expression of the stem cell marker *Lgr5* and an increase of the goblet cell marker *Muc2*. Importantly, these changes are similar between organoids deleted for *Cdk8/19* and WT organoids treated with the CDK8/19 inhibitor Senexin B. Taken together, these results show that there is no major change in differentiation in intestinal organoids but there is a clear increase in Muc2 expression. However, we cannot entirely rule out slight changes in differentiation.

3. Protocols for Cdk8 depletion in vivo. It seems that Cre was induced by a single IP injection of tamoxifen + 5 days with tamoxifen diet. Why this diet was not continued for more days? In other models, it is not unusual to keep mice on tamoxifen for months. A single injection + 5 days on diet are very likely to be insufficient for promoting excision of Cdk8 in many cells (e.g. quiescent progenitors, etc.).

This is not an issue. A single injection + 5 days of tamoxifen diet is enough to completely remove *Cdk8* (NB: in the intestinal epithelium, the stem cells are highly proliferating and not quiescent). As indicated in the cross-comments by referee 3, and as mentioned above, in original Fig 1A we show a complete absence of CDK8 immunostaining in small intestine of $Cdk8^{-/-}$ mice collected two months after tamoxifen treatment, which indicates that recombination took place from the beginning. If it were inefficient, the crypts would undoubtedly be repopulated by un-recombined stem cells.

4. The problem indicated above may be especially important in AOM/DSS induced pathologies. Mice were treated with tamoxifen as indicated above and treated later with the carcinogens. I don't understand this protocol. Carcinogen treatment may activate cells (e.g quiescent progenitors that are still WT for Cdk8. Why tamoxifen was not given (also) after carcinogen treatment. I am afraid that the way these protocols were performed may be hiding any potential effect of Cdk8 knockout.

Please also see our comment above in response to point 3. We understand that the reviewer noted that in some mice treated with AOM-DSS, background staining of CDK8 could be detected, but this is negligible and likely to be due to the difficulty in obtaining a pure population of cells for western blotting; in other mice, there was no background, and no mice revealed any effects of CDK8 deletion on the phenotype. Figure 1A and PCR genotyping (Fig EV1C) shows that the deletion is highly effective.

Other points
Fig. 2D. are the differences between double KO and single KOs or WT significant at day 6?

The reviewer's comment made us realise that the way we had presented the data is not the easiest in order to appreciate differences in growth, and we have presented these comparisons differently in the revised version. In brief: what we show in this figure is a clear increase in size in WT and single KOs which is not the case for the double mutant, indicating that these organoids

grow more slowly. We have done statistical tests on the 6-day samples, as suggested. We have also performed a longer term CDK8/19 inhibition experiment over 9 days, and found a statistically significant decrease in organoid growth, as shown in new Figure 2G.

Methods: "Cdk8 lox/lox mice were crossed with Villin-Cre-ERT2 +/- mice to obtain Cdk8 lox/lox, Villin-Cre-ERT2 +/-" seems a too obvious sentence.

We agree, and have edited this sentence.

Cdk19 CRISPR knockout seems to be at least partially repeated in two sections (Organoids and CRISPR)

We have eliminated this redundancy.

Reviewer 2.

In recent years, enhancer-promoter interactions have received significant attention as they underlie 3D chromatin architecture and the transcriptional program of cell identity. Transducing information from enhancers to the correct promoters, the Mediator complex (containing the analogue kinases CDK8/19) is pivotal for many processes and defining cell identity. This manuscript addresses several outstanding questions in the field of transcriptional CDKs, and more widely, relating enhancers, Mediator, and the core transcriptional machinery to cancer susceptibility and cystic fibrosis.

Here, Prieto and colleagues have investigated the role of the kinases CDK8 and CDK19 by conditional deletion of one or both genes in the mouse intestine, and in derived intestinal organoids. They have examined the effect of CDK8/19 deletion on the phosphorylation of RNA Pol II (a reported target of CDK8 activity, although in early papers, in vitro), global gene expression, cell proliferation, tumorigenesis, and differentiation capacity. Their data reveals the extent to which these sister kinases have independent or redundant functions, an under-explored aspect of Mediator function, since only one may occupy the Mediator complex at a time. Moreover, given the central position of Mediator in the enhancer-promoter interaction, and the fact that the CDK8/19 kinases represent the only enzymatic subunits of Mediator, Prieto and colleagues report that double-deletion of these kinases has a surprisingly limited effect on intestinal organoid differentiation capacity and tumorigenesis. The Authors then use their intestinal organoid system to identify a link between CDK8/19 and cystic fibrosis, specifically the CFTR pathway.

The methods and experiments using tissue-specific knockout of CDK8/19 in vivo and in intestinal organoids in vitro, appear robust. The new findings of Prieto et al will surprise many readers, as they reveal that, in some contexts, CDK8 and CDK19 are not essential, unlike general transcriptional machinery. Importantly, the Authors combine genetic and chemical methods to convincingly dissect the individual vs combined functions of the sister kinases CDK8 and CDK19, as well as separately investigating their kinase activity, important nuances which are nevertheless rare in the field. Lastly, the studies using CDK8/19 conditional deletion in organoids leads to a novel link between CDK8/19 function and Cystic Fibrosis.

As a result, overall, the manuscript is likely to be of interest to a wide audience since it explores new and important avenues on the role of CDK8/19 and Mediator in cancer, development and cystic fibrosis. I thank the Authors for an interesting read. Below I outline some Major and Minor comments:

We would like to thank the reviewer for his or her careful reading and thoughtful analysis, with which we entirely agree.

Reply to reviewer 2

Major Comments

Cystic Fibrosis (CF) patients lose CFTR function and are highly susceptible to early, aggressive colorectal tumor development. Similarly, based on the Authors data, a CDK8/19 small molecule inhibitor might be expected to reduce CFTR expression in the intestine in vivo. This would have wider implications for many readers, concerning the concept of using CDK8/19i in vivo in future clinical applications. The manuscript would benefit if the Authors examine whether the CFTR becomes reduced in vivo in the intestine, in mice treated with CDK8/19i, or a CF-like phenotype in mouse intestine treated with a CDK8/19-inhibitor. In any case, the Authors should comment on this possibility given its wider implications for the use of CDK8/19 inhibitors in humans.

This is a very good suggestion, and we would have liked to have already done these experiments but we have only recently acquired a sufficient quantity of inhibitors to do so. We have now performed the suggested *in vivo* experiments with a well characterised and highly specific CDK8/19 inhibitor, Senexin-631, developed by Igor Roninson (University of South Carolina). The result was striking: we found that CDK8/19 inhibition for four days caused a significant increase in mucin staining in the intestine, as shown in new Fig 6. This effect wore off over time as at day 11 there was no longer any visible difference in mucin staining, indicating adaptation to the inhibitors. Interestingly, we found that there was a strong upregulation of CDK8 and CDK19 in response to long-term treatment with inhibitors, which may well tend to counteract the effect (and incidentally gives a very good biomarker for CDK8/19 inhibition *in vivo)*. There was no acute toxicity of these inhibitors: the mice tolerated the inhibitors well with no other strong complications of CF observed (weight loss, intestinal occlusion, etc; see new Fig EV10). These results are important both with regards to the mechanism of mucin expression and secretion, which depends on CDK8/19 kinase activity, as well as regarding potential usefulness and toxicity of inhibitors. One potential advantage of CFTR inhibition could be to reduce acute fluid loss in secretory diarrhea (a major global health problem due to parasites and drug side-effects); as CDK8/19 inhibitors appear to work in the short term, at least, and can be well tolerated *in vivo*, future studies will address possible usefulness in this regard.

Main Fig1, Fig2, and Fig4 are very clear.

Thank you for this appraisal.

Fig3: The Authors describe in the Results section that CDK8-KO intestinal epithelium shows almost no transcriptomic changes versus wild-type intestine (DEG = 4 genes). In contrast, CDK8-KO intestinal epithelium organoids show 716 up, 575 down, total DEGs = 1291 genes. Even with a 2x threshold, DEG's = 390 genes (Figure 3A). Can the Authors comment on this difference?

This is a good point and we are happy to elaborate. We suspected that the lack of statistically significant transcriptomic changes in the CDK8-KO intestinal epithelium (DEG = 4 genes in our original analysis) may well have more to do with the complexity and biological variability of the system, and the fact that we only had replicates. In such cases, the statistical power of the analysis of the *in vivo* RNA-seq is reduced. We have now investigated this hypothesis. The original transcriptome analysis was performed in 2017 using a pipeline that has since been superceded; we reanalysed the data applying the same pipeline as for the organoid RNA-seq and found 31 differentially expressed genes (see new Table EV1), even given the lack of additional replicates; indeed, the variance is limiting. Finally, to avoid any potential misinterpretation, we have modified the wording of this section: the fact that many genes *in vivo* do not pass a threshold p-value for differences being statistically significant does not necessarily mean that they are not deregulated; simply, that we do not have the statistical power to claim that they are.

Moreover, in the Discussion section, the Authors make a conclusion that needs clarification or qualification, as it appears at odds with the data: "Our results also do not support an essential role for Mediator kinases in general gene expression, in contrast to Mediator itself, since relatively few genes were highly deregulated in double knockouts." However, this only appears to apply to the

in vivo intestinal tissue for CDK8-KO, but not the organoids for CDK8-KO or CDK8/19-dKO -can the Authors include this nuance in the Discussion, and comment on reasons for this large difference? In particular, the data does show a very large effect on gene expression for CDK8/19-double KO, where it appears > 3600 differential genes were observed (Figure 3B). Therefore the current statement seems out of place, and may require re-phrasing.

In the Discussion section we had indeed concluded that "Our results also do not support an essential role for Mediator kinases in general gene expression". Although perhaps this is open to misinterpretation (and has been re-phrased in our revised version), we meant to contrast it to what is observed when essential proteins of the Mediator complex (*e.g.* Med14) are removed. In this case, a global shift in the whole transcriptome occurs within 60 hours, with a mean downregulation of a factor of 7, as shown in:

El Khattabi, L., Zhao, H., Kalchschmidt, J., Young, N., Jung, S., Van Blerkom, P., ... & Casellas, R. (2019). A pliable mediator acts as a functional rather than an architectural bridge between promoters and enhancers. Cell, 178(5), 1145-1158.

Finally, even though we found 3182 differentially expressed genes in the CDK8/19 KO organoids, for the majority of these the fold-change is less than two, which may not be biologically very meaningful.

This has now been explained better.

Can the Authors clarify if their RNAseq expts with a "spike-in" to detect a global shift in the whole transcriptome? (This is not mentioned in the Methods) RNAseq has an internal normalization that can hide a global shift in the whole transcriptome (Loven J et al., 2012, Cell, PMID: 23101621). This might apply in a situation like this manuscript where the core transcriptional machinery is being manipulated. It is perfectly acceptable if no spike-in was included, not everyone needs to do this, however it might offer an explanation for why few genes might be observed as differential in the case of the intestinal epithelium.

This is an astute comment, and we also worried (as one should when knocking out the only two kinases known to regulate Mediator) that we might normalise out a shift in the whole transcriptome. We thus employed ERCC spike-in RNAs for RNA-seq of the organoids, added in proportion to the genomic DNA isolated in parallel with the RNA (in case rRNA was also altered, we did not spike in proportion to total RNA). We compared results between library normalisation and spike-in normalisation, and there were no global differences (as shown in new Fig EV8A). The magnitude of the fold-change was generally slightly smaller for the spike-in normalisation (see figure below). This is almost certainly due to the high dispersion of spike-in normalisation factors for the WT samples, that range from 0.51 to 1.04 (average 0.8), compared to library normalisation factors, which vary from 0.97 to 1.00 (average 0.986). Library normalisation is therefore superior and we present these data.

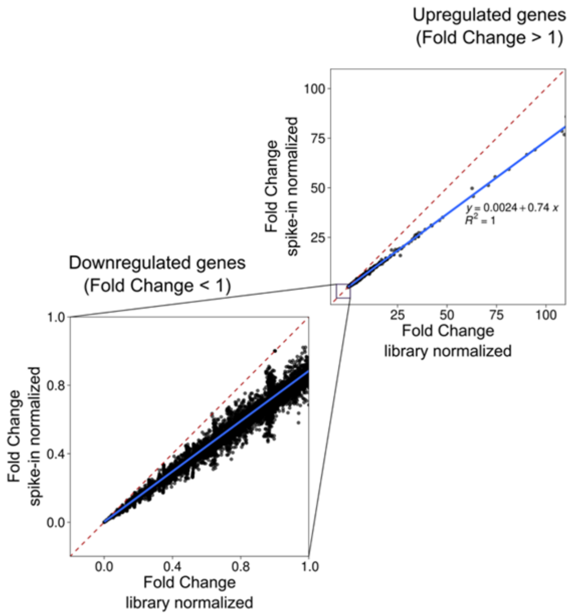

Reply to reviewer 2

The Authors mention in their Discussion section: "we found that there was a strong overlap between transcriptome changes of double knockout organoids and intestinal knockout of the gene encoding CFTR...." However I did not find the Figure reference for the comparison of transcriptomic changes in CDK8/19i vs CFTR-KO, as mentioned -can the Authors please clarify this, or show this analysis?

We apologise for not having shown this data, which is now presented in Venn diagram format in new Fig 4B and the data in a Supplementary Table EV3. This shows that 98% of genes deregulated in the small intestine in CFTR knockouts are also deregulated in CDK8/19 knockouts. Most CFTR KO upregulated genes are similarly deregulated in CDK8/19 knockouts, but only a minority of downregulated genes.

Minor Comments

Introduction: the statement: "CDK19 deletion has not yet been reported." I suggest to specify that this applies in vivo, highlighting the novelty of the new study. In vitro, CDK19 has been knocked out in a number of cell types.

While our paper was in revision, a competing lab published (Dannappel, JCI, Aug 25 2022) that mice obtained from the Jackson laboratory are "viable and generally healthy", and we cite this study.

In this sentence in the Results section, I think the word "as" might be missing. Otherwise it is not clear: "We found that Senexin B treatment for 24h leads to the downregulation of Cftr and upregulation of Muc3 expression (Fig 4G) "AS" seen upon genetic ablation of both Cdk8 and Cdk19, indicating that kinase activity of CDK8/19 controls CFTR pathway gene expression."

In this sentence in the Results section, I think the word "they" refers to "CDK8/19", but it could alternatively refer to "quiescent cells" -I suggest to re-phrase to clarify: "....(Koehler et al, 2016) our data suggest that in the intestinal epithelium, cells devoid of both CDK8 and CDK19 have an increased tendency to become quiescent, implying that they provide a growth advantage."

Thank you for pointing out these glitches; we have included all the modifications suggested.

Reviewer 3

Using conditional "floxed" KO mouse models and small intestinal (SI) organoids generated from these mice or gene edited by CRISPR/Cas9 to obtain conditional double KOs for the cyclin-dependent kinases CDK8 and CDK19, this study shows rather convincing evidence for a functional redundancy of these highly conserved kinases in the control of gene expression by the essential Mediator gene transcription complex. However, the combined deletion or pharmacological inhibition of CDK8 and CDK19 in intestinal organoids is not lethal but reduces proliferation, results in loss of Cyclin C, and induces a cystic fibrosis-like phenotype, characterized by a partial loss of CFTR expression and function, and mucus (Muc2 and 3) accumulation and hypersecretion by goblet cells. The models used and the findings obtained in this study are robustly documented and are new and of considerable scientific and medical interest in the area of tumor biology and CF.

We thank the reviewer for this appreciation.

A major limitation is however its focus on a single species (adult mice, not humans) and tissue (small intestine).

Reply to reviewer 3

It is of course true that the results would have even more impact if it could be shown that they apply to humans and / or airway tissue, but we felt it important to document our results in a first discovery paper involving nearly ten years of work, opening the way for exploring this further.

Yet there are several points of criticism that require further attention.
Specific comments:
1. On p. 4, 13 l. up the authors justify the use of Cdk8/Cdk19 double KO intestinal organoids rather than mice as a model by claiming that Cdk8/Cdk19 double knockout in adult mice is most probably lethal. However, it is unclear why they did not attempt to generate Cdk8lox/lox/Cdk19lox/lox mice and to cross them with a Tamoxifen-inducible Cre expressed under control of the intestine-specific villin promoter. Assuming that the double KO would slow down intestinal cell proliferation but is not lethal and allows differentiation, as reported for the organoids, Tamoxifen treatment is not expected to induce a severe morbidity or lethality at the intestinal level on the short run. Because the intestinal phenotype of CF mice has been described in much detail in the literature (e.g. see Kreda et al Cold Spring Harb Perspect med 2012; 2:a009589; Ikpa et al Genomics 2020; 112: 1139), confirmation of the CF-like phenotype in double Cdk8/Cdk19 knockout mice in vivo rather than in organoids would further strengthen this study.

While it would indeed be ideal to have a double conditional CDK8/19 knockout, such a model did not exist and would take a long time for our lab to generate. Yet to address this issue, and also to examine the effect of inhibition of both CDK8 and CDK19 *in vivo*, we performed additional experiments in mice with the newest CDK8/19 inhibitor, which has been validated *in vivo*, Senexin 631 (Ding et al., PNAS, 2022). As described above in our response to suggestions of reviewer 2, we found two quite striking observations: the first is that CDK8/19 inhibition *in vivo* almost exactly recapitulated the phenotype of double knockout organoids *in vitro*, *i.e.* a strong increase in mucin staining, while the second is the strong compensatory upregulation of CDK8 and CDK19 protein levels (thus constituting a good in vivo marker for CDK8/19 inhibition). The latter observation may explain why the mucin phenotype was only transient, as it was observed at 4 days but no longer at 11 days, and it may have reduced the toxicity of the treatment, which was well tolerated.

Given the extreme conservation of these two kinases, the only kinases known to regulate Mediator, and the fact that double knockout organoids lose viability over time, we expected a lethal phenotype from intestine-specific double mutant mice. This is the main reason we did not cross our conditional *Cdk8* mutant mice to the *Cdk19* CRISPR mutant mouse, which was available from the Jackson institute. We also knew that the Firestein lab (Melbourne, Australia), with whom we were in touch, was performing such experiments and it did not make sense to duplicate efforts. The Firestein lab published their paper with exactly this model in JCI on August 25th 2022, *i.e.* while we were finishing revisions (Dannappel et al., 2022, *J Clin. Inv.*). Like us, they conclude that CDK8 and CDK19 are dispensable for cell proliferation and differentiation in the intestinal epithelium, although they did not study the CFTR pathway. However, they observed a reduction in the proportion of cells issued from the secretory lineage in double knockouts and interpreted this as being evidence for a lineage switch regulated by Mediator kinases. We find no such evidence and were surprised by what appears to be hyperplasia of secretory cell types in the intestinal epithelium of their wild-type mice, which show far more goblet cells and tuft cells per villus than are typically seen (*c.f.* Gerbe et al., 2016, *Nature*), possibly reflecting the presence of pathogens, whereas their *Cdk8/Cdk19* knockouts show no such hyperplasia. Along with the absence of a change in the balance of secretory lineage cells in *Cdk8/Cdk19* knockouts in our experiments, and the accumulating evidence that CDK8/19 are required for pro-inflammatory responses (Chen et al, 2017, *PNAS*; Johannessen et al, 2017, *Nat Chem Biol*) we think it is unlikely that Mediator kinases control a lineage switch in the intestine but plausible that they are required for mucosal immune responses. We discuss this in the revised version of our paper.

2. CDK8/19 deletion or Senexin B inhibition of the kinase activities in the SI organoids resulted in the gradual loss of CFTR expression, CFTR protein, and CFTR function, as measured by qRT-PCR, WB, and the organoid swelling (FIS) assay, respectively (Fig. 3, D,E; Fig. 4, E-G). However, these observations raise several questions: (i) (WT) CFTR transcripts are down by only ~25% in Cdk8 KO

organoids and by ~50% in the double KOs (Fig. 3D). It is well known that a loss of 50% WT CFTR expression and activity as seen in CF carriers (CFTR+/severe mutation) does not cause any pathology and is beyond the dynamic window of the FIS assay. It is more likely, therefore, that CDK8/19 deletion results in a much more dramatic loss of expression and function of other transporters or regulators required to allow the forskolin-activated, CFTR-dependent anion secretion measured by FIS, e.g. NKCC1, NBCe1, adenylyl cyclase or PK-A (please note that anion and fluid secretion in 3D organoids as monitored by FIS is also dependent on Cl- or HCO3- import across the basolateral membrane of the enterocytes and on AC and PKA activity). Therefore the authors need to verify in their RNA-Seq data base (Fig, 3B) whether one or more of these genes are strongly downregulated in the double KO mice;

This is an excellent suggestion, that we have implemented. We surmised that the 25% difference in mRNA, which takes into account both transcription and degradation, might be reflected in larger changes at the protein level. This involved acquiring new antibodies from the Cystic Fibrosis Foundation (University of North Carolina) which took some time (see reviewer's point (iii), below). We have thus performed new western blots to re-examine the levels of CFTR protein in *Cdk8*$^{-/-}$/ *Cdk19*$^{-/-}$ organoids. The band migrating in WT organoids at the same height as the CFTR positive control is almost completely absent in the double knockout (as seen in the CFTR negative control; see new Fig. 4D). We also blotted intestinal tissue from our new experiment in which we treated mice with CDK8/19 inhibitor (see response to comment (i), above). Strikingly, the CFTR band is increased in intestinal tissue after 4d of treatment where the CF-like phenotype is seen, like the increase in CDK8/19 expression, suggesting a compensation (new Fig 6A).
Second, indeed, there are many solute carriers (95) whose expression is deregulated in CDK8/19 knockouts (new supplementary Table EV5), including four inorganic anion transporters of the Slc26 family, all of which are upregulated. NKCC1 (Slc12a2) is not identified but its putative inhibitor Slc12a9 is upregulated. We also do not identify NBCe1 (Slc4a4) as deregulated, but a homologue (Slc4a2) is upregulated. 22 solute carrier genes have reduced expression. Some of these changes may either contribute to or be compensatory for reduced CFTR pathway function. We have now discussed these results and modified the conclusions to avoid the appearance of making excessive claims that the phenotype is entirely and directly due to loss of CFTR expression, even though this remains a possibility.

(iii) (NB: we did not find the reviewer's "(ii)") Western blotting or ICH of rodent CFTR is notoriously difficult to perform because of a lack of specific monoclonal antibodies, and is at best semi-quantitative. It is doubtful whether the band stained by the monoclonal CFTR Ab2784 in Fig. 3E represents the C-band of complex-glycosylated CFTR, which normally is broad and very diffuse. Therefore the whole blot rather than a small section of the blot should be made accessible to the reviewer, and the identity of the band and the specificity of the antibody for mouse CFTR should be verified by WB of a Cftr-/- mouse. Also the number of blots examined should be given. Considering the 1363 genes that are downregulated in the double KO organoids (Fig. 3B), there are multiple candidate proteins that could possibly crossreact with the Abcam antibody;

We agree that these blots are technically difficult, especially from intestinal tissue. As explained above, we have acquired new CFTR antibodies as well as positive and negative controls and confirmed our results using these antibodies (new Fig 4D), which indeed show broad and rather diffuse staining. Overexposure of whole blots (right) shows that the minor bands are cross-reacting proteins as they are present in the negative control.

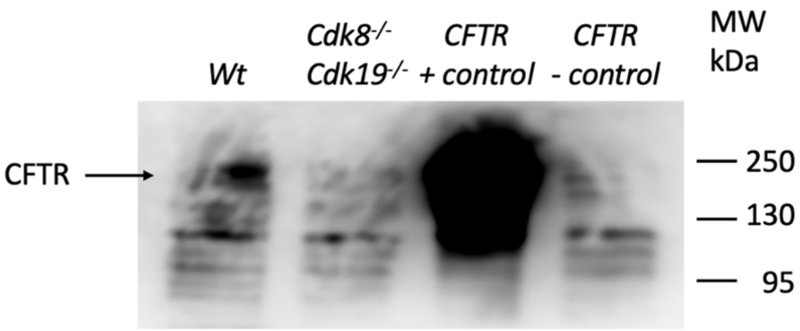

(iv) a more direct assessment of CFTR activity in intestinal organoids can be achieved by electrical current measurements across monolayers of organoids permeabilized basolaterally by a pore-forming agent, e.g. nystatin.

This is a very interesting suggestion but we could not find collaborators who were available to help with such experiments, and we will have to defer this to a follow-up study.

3. Strong downregulation of CFTR and upregulation of Muc2 in goblet cells is also seen upon differentiation of WT intestinal organoids by removing Wnt3a from the culture medium and/or by inhibiting Notch signaling by the γ-secretase inhibitor DAPT. Would it be possible, in analogy, that the KO of CDK8+19, or the inhibition of cyclin-dependent kinase activity by Senexin B results in the repression of genes in the Notch signaling pathway, explaining the partial inhibition of cell proliferation (Fig. 2F) and the conversion into a mucoid phenotype? Did pathway analysis of the RNA-Seq data showed downregulation of the Notch signaling components?

This is another excellent suggestion, especially since CDK8-cyclin C has already been implicated in control of the Notch pathway (Li et al., Nat Cell Biol, 2014). Yet loss of cyclin C was found to upregulate, rather than downregulate the pathway. In our RNA-seq and new qRT-PCR data from double knockout organoids, there was indeed, at the transcriptome level, a significant downregulation of Notch1 (see new Fig 3E), but Hes1, one of the main targets of Notch1, was not altered. Since these are population studies, we cannot rule out bigger changes in individual cell types such as goblet cells. Thus, it remains possible that altered activity of the Notch pathway might contribute to some of the phenotypes. Further single cell studies will be required to explore this in detail. This has been added to the discussion.

4. Fig. 3B: How do the authors explain that in the Venn diagram only 27 and 14 out of the 155 respectively 151 genes that are up-or downregulated in the Cdk19-/- organoids are also up-or downregulated in the double KO organoids? How can the additional knockdown of Cdk8 result in the loss of up-and downregulation of 128 (131 =129+2) respectively 137 (129 +8) genes?????

This is a very astute comment, and we have explored this further. We first ruled out several potential artefacts, whereby double knockouts might have greater variance than single knockouts for these particular genes. This is not the case for genes other than those grouping to very low variance: see graph in new Fig EV8B. We also ruled out a potential bias of these genes to low read counts; see graph in new Fig EV8C. We thus considered the possibility that some genes might be dominantly controlled by, or specific to, CDK8 or CDK19. As such, additional knockout of the second Mediator kinase might counteract the effect of the first knockout. Thus, we looked at read counts for each gene deregulated only in single *Cdk8* or *Cdk19* knockouts but not double knockouts. This revealed that essentially all genes specifically deregulated in single *Cdk8* knockouts are also somewhat deregulated in the same direction in double knockouts, but to a lesser degree. In almost all cases, the same genes were either not deregulated in *Cdk19* knockouts or somewhat deregulated in the opposite direction. Interestingly, the same is not true for genes only deregulated in single *Cdk19* knockouts: the double knockouts more resemble single *Cdk8* knockouts rather than showing an intermediate level of expression between the single and double knockouts. These data are presented in Principal Component Analysis graphs in new Fig EV8D and E. This analysis highlights the fact that the failure to pass a threshold for statistical significance for being deregulated is not synonymous with not being deregulated, a common misconception when interpreting gene expression data; and that gene expression networks are inherently complex: effects of combined knockouts are not always additive. Moreover, it suggests that CDK8 exerts a stronger influence on gene expression than CDK19 (consistent with the reported higher expression levels of CDK19), and that some genes are regulated in opposite directions by CDK8 and CDK19; *i.e.* although CDK8 and CDK19 appear largely redundant, some genes may be specifically regulated by one or the other kinase. As such, the suggestion was very useful and allowed us to strengthen message of the paper.

Reply to reviewer 3

5. Fig. 4F: The extent of FIS inhibition in the Cdk8-/-/Cdk19-/- mice (~80% of WT) was much higher than the inhibition by saturating concentrations (10 uM) of SenB (<40% of WT), suggesting that the loss of CDK proteins has more impact on CFTR function than the loss of CDK kinase activity. Therefore the claim that "the kinase activity of CDK8/19 controls CFTR pathway expression" (p. 5, 9 l. up) should be toned down.

This is a fair comment and we have revised the description of these results to be more cautious. It is worth mentioning that we showed that the effect of the inhibitor was not seen after only one hour, but was clearly visible after 24h, suggesting that it is a consequence of loss of transcription rather than a regulation of the pathway via direct phosphorylation by Cdk8/19. It is also worth mentioning that inhibitors are not "on/off" switches, we have to take into account that they are ATP competitive inhibitors and do not completely kill the kinase activity even at micromolar concentrations, since intracellular ATP is millimolar. In this context, using our new CFTR antibodies, we found that 24h Senexin B inhibition of CDK8/19 in organoids caused a much smaller reduction of CFTR expression than *Cdk8/19* knockout (new Fig 5H).

6. Fig. 4G: MUC2, not MUC3 is the major secreted mucin in mouse intestine. Was the expression of the Muc2 gene also upregulated by SenB?

Our new qRT-PCR experiment (new Fig 3D) confirms that Muc2 is slightly upregulated in intestinal organoids upon Senexin B treatment, similarly to the double Cdk8/19 knockout.

7. To allow a fair comparison, was the genetic background of the WT mice or intestinal organoids always identical to the one of the KO mice/organoids?

Yes, the genetic background of all mice and organoids was identical; this has been mentioned in the revision.

8. In the Method section, the description of the CRISPR/Cas9 editing of the organoids on p. 8, ala. 3 is highly repetitive with the text on p. 13, ala. 2. Please correct this duplication.
9. Some minor typo's should be corrected, e.g. p. 13, l. 6: deferentially=differentially; p. 19, 4 l. up: umpaired=unpaired

Thank you for pointing out these errors, which have been corrected.

Cross-comments by referee 3:

On Referee 1:
- I am less concerned about the effectivity of Cdk8 ablation in vivo, considering the complete absence of CDK8 immunostaining in small intestine of Cdk8-/- mice collected two months after tamoxifen treatment (Fig. 1A).

Please also see our reply to referee 1.

- I agree that the relationship between CDK8/19 KO and the "CFTR pathway" is descriptive rather than mechanistic, and could be simply due to enhanced differentiation of the organoids (e.g. by Notch inhibition) which strongly reduces CFTR expression and function in intestinal organoids. As suggested in my report, it is even not certain that the inhibition of organoid swelling in the double KO is due to a loss of CFTR expression, more likely it results from loss of chloride import through NKCC1 or the loss of upstream signaling. Because the authors have bulk RNA Seq data, it is easy for them to explore such possibilities.

As described in our detailed reply, above, we have explored these possibilities and report the data in the revised version. Please also see our comment in the general reply to reviewer 1 about

previous claims in the literature about the mechanisms of action of CDK8/19 on gene expression, which, in our view, do not genuinely constitute convincing mechanisms.

On Referee 2
- I agree with his/her positive and negative comments, in particular with major comment 1 and 3. It remains possible that CDK8/19 KO or inhibition of the kinases in organoids would only turn undifferentiated, crypt-like and CFTR-rich organoids into differentiated, villus-like organoids and therefore would not trigger a CF-like phenotype in mice in vivo. This might also explain why CDK8 KO in organoids but not in mice in vivo causes major changes in gene expression. The in vivo double KO could be made chemically by treatment with CDK8/19i or senexin B (but this will also affect multiple other tissues), or, preferentially, genetically by generating conditional double KO mice (cf. my report, comment 1). But perhaps the authors have already tried one or more of these approaches and have strong arguments why intestinal organoids are the best/only usable models.

As described above, we have now used CDK8/19 inhibitors *in vivo*, which recapitulate the increased Mucin phenotype until adaptation occurs, and characterised better the WT and mutant organoids in terms of cell types. While some data suggest slight differences in stem cell and differentiation markers, these are not sufficient to state that these kinases promote the maintenance of an undifferentiated state.

Reviewer#1 Specific comment#1: The Authors may be able to address this relatively easily. Perhaps a published ChIP-seq of CDK8/19 might reveal binding proximal to the CTFR genes, providing a mechanistic direct link to the expression of CFTR?

We have looked at this but not found any evidence for it; please also see our reply to reviewer 1 on the ChIP-seq question.

Reviewer#1 Specific comment#2: The Reviewer is concerned that CDK8/19-dKO organoids might show a CF-like phenotype due to indirect effects, such as decreased proliferation or abnormal differentiation of different lineages. The Authors already show no effect on intestine cell composition or proliferation by CDK8-KO, in Fig.1. The Authors could perhaps extend this analysis into intestinal organoid cell composition, and using a CDK8/19-dKO context, using IHC or IF to characterize the major cell types present, and proliferation (ki67). The existing RNAseq might also indicate whether there is decreased proliferation or abnormal differentiation of different lineages. Related: It has been shown that CFTR null mice have increased (rather than decreased) epithelial cell proliferation (PMID: 11518680).

As explained in detail above, we have performed the suggested analysis, and we have also cited the suggested paper.

Reviewer#1 Specific comment#3-to-end: These concerns of the Reviewer may require only clarification or explanations by the Authors. This could be textual.

Indeed, as described above, we have clarified these points and present the analysis of organoid proliferation in a way that should be easier to understand.

Cross-referee comments from Reviewer#2 on Reviewer#3

Reviewer#3 Specific comment#1: Perhaps the Authors could easily address this point by one or two approaches; (i) providing any more information if they have attempted to make CDK8/19-dKO mouse intestine. The Authors say they anticipate that CDK8/19-dKO in intestine might be lethal -can they confirm that they tried this or elaborate on why they think it would be lethal? (ii) If the Authors have treated WT mice with CDK8/19i small molecule inhibitors, did they see lethality?

We have elaborated on why we thought intestinal double knockout mice would be lethal, and have performed experiments using WT mice with CDK8/19 inhibitors, as described above.

Reply to cross commenting

Reviewer#3 Specific comment#2+3: The Reviewer has a main concerned that CDK8/19-dKO might affect the expression of many ion channels, so that the CF-like phenotype observed is not solely dependent on CFTR. Perhaps the Authors could address this point by rescuing CFTR expression. If CFTR expression levels are returned to normal in the CDK8/19-dKO organoids, does this rescue the CF-like phenotype. I suggest that this might address this Reviewer concern, and provide further support to a central message of the paper. CFTR expression levels could be supported by exogenous over-expression, or CRISPRa of the endogenous CFTR, for example, in CDK8/19-dKO organoids, or WT organoids treated with CDK8/19i.

These are good suggestions, but we think that the idea of reviewer #3 is very likely and that the phenotype might not solely be due to CFTR downregulation. As such, we have explored contributions of other genes to the CF phenotype, as described above, and modified the conclusions of the paper accordingly. Rescue experiments to ensure delivery of ≈ 5kb of cDNAs to all cells of mutant organoids that do not proliferate well would be technically extremely challenging.

Reviewer#3 Specific comment#4-9: these concerns of the Reviewer may require only clarification/explanations by the Authors. This could be textual.
Or in the case of antibody specificity for CFTR -the Authors could perform some reasonable check and controls on the antibody specificity, as the Reviewer#1 suggests.

As detailed above, we have acquired other antibodies and controls using CFTR mutant tissue and performed new analyses.

1st Revision - Editorial Decision 23rd Nov 2022

Dear Daniel,

Thank you for the submission of your revised manuscript. We have now received the enclosed reports from referees 1 and 2. Both referees only have one more minor suggestion that I would like you to address and incorporate before we can proceed with the official acceptance of your manuscript.

A few editorial requests will also need to be addressed:

- Please make sure that your publicly deposited data are freely accessible upon the online publication of your manuscript.

- Please rename the subtitle "Conflict of Interest" to "DISCLOSURE AND COMPETING INTERESTS STATEMENT".

- Please remove the author credits paragraph from the ms file. We now use CRediT to specify the contributions of each author in the journal submission system. CRediT replaces the author contribution section. Please use the free text box to provide more detailed descriptions, if you wish. See also our guide to authors https://www.embopress.org/page/journal/14693178/authorguide#authorshipguidelines.

- Some funding grant numbers are missing in our online submission system, please add.

- Please combine the movie legend with the movie in a zipped file and upload this as one file. The movie legend should be removed from the ms file.

- Your manuscript has 10 EV figures, but our publisher can only process 5. You can either move 5 EV figures to an Appendix file, or move all EV figures into a single pdf Appendix file. The appendix file needs a table of content with page numbers. You can find more info in our guide to authors online. The nomenclature of Appendix figures is Appendix Figure S1, etc. Please also correct all callouts in the ms text.

- Tables EV2-4 should be renamed to Dataset EV2-EV4 with their corresponding legends in the same files (can be in a separate sheet). Table EV5 should be renamed to Table EV2, and the callouts need to be corrected accordingly

- I attach to this email a related ms file with comments by our data editors. Please address all comments in the final ms file.

EMBO press papers are accompanied online by A) a short (1-2 sentences) summary of the findings and their significance, B) 2-3 bullet points highlighting key results and C) a synopsis image that is exactly 550 pixels wide and 200-600 pixels high (the height is variable). You can either show a model or key data in the synopsis image. Please note that text needs to be readable at the final size. Please send us this information along with the revised manuscript.

I look forward to seeing a final version of your manuscript as soon as possible. Please use this link to submit your revision: https://embor.msubmit.net/cgi-bin/main.plex

Best regards,
Esther

Esther Schnapp, PhD
Senior Editor
EMBO reports

Referee #1:

The authors have made a major effort to address many of the concerns with the methodology and the conclusions in the previous version. The new data using the Cdk8/19 inhibitors are very helpful given the lack of an in vivo double knockout model. Analysis of RNAseq data is also improved and, together with the new characterization of specific cell types, many conclusions have been clarified with new results and the discussion.

Minor observation: Page 5.: "We performed RNA-sequencing from the intestinal epithelium of wild-type and knockout mice, but could identify only 31 genes with statistically significant expression alterations due to biological variability between animals and the limited number of replicates". I find a bit unusual to conclude in these first sentences that the only genes with statistical differences are not real hits.

Referee #2:

REVIEWER2 RESPONSE TO AUTHOR REBUTTAL1

Reviewer2: please note:
-I have given my response to the Authors rebuttal1, below, after each point.
-As requested by the Editor, I have also responded in place of Reviewer3, further below, in the Reviewer3-sections.

Reviewer 2.
In recent years, enhancer-promoter interactions have received significant attention as they underlie 3D chromatin architecture and the transcriptional program of cell identity. Transducing information from enhancers to the correct promoters, the Mediator complex (containing the analogue kinases CDK8/19) is pivotal for many processes and defining cell identity. This manuscript addresses several outstanding questions in the field of transcriptional CDKs, and more widely, relating enhancers, Mediator, and the core transcriptional machinery to cancer susceptibility and cystic fibrosis.

Here, Prieto and colleagues have investigated the role of the kinases CDK8 and CDK19 by conditional deletion of one or both genes in the mouse intestine, and in derived intestinal organoids. They have examined the effect of CDK8/19 deletion on the phosphorylation of RNA Pol II (a reported target of CDK8 activity, although in early papers, in vitro), global gene expression, cell proliferation, tumorigenesis, and differentiation capacity. Their data reveals the extent to which these sister kinases have independent or redundant functions, an under-explored aspect of Mediator function, since only one may occupy the Mediator complex at a time. Moreover, given the central position of Mediator in the enhancer-promoter interaction, and the fact that the CDK8/19 kinases represent the only enzymatic subunits of Mediator, Prieto and colleagues report that double-deletion of these kinases has a surprisingly limited effect on intestinal organoid differentiation capacity and tumorigenesis. The Authors then use their intestinal organoid system to identify a link between CDK8/19 and cystic fibrosis, specifically the CFTR pathway.

The methods and experiments using tissue-specific knockout of CDK8/19 in vivo and in intestinal organoids in vitro, appear robust. The new findings of Prieto et al will surprise many readers, as they reveal that, in some contexts, CDK8 and CDK19 are not essential, unlike general transcriptional machinery. Importantly, the Authors combine genetic and chemical methods to convincingly dissect the individual vs combined functions of the sister kinases CDK8 and CDK19, as well as separately investigating their kinase activity, important nuances which are nevertheless rare in the field. Lastly, the studies using CDK8/19 conditional deletion in organoids leads to a novel link between CDK8/19 function and Cystic Fibrosis.

As a result, overall, the manuscript is likely to be of interest to a wide audience since it explores new and important avenues on the role of CDK8/19 and Mediator in cancer, development and cystic fibrosis. I thank the Authors for an interesting read. Below I outline some Major and Minor comments:

We would like to thank the reviewer for his or her careful reading and thoughtful analysis, with which we entirely agree.

Major Comments

Cystic Fibrosis (CF) patients lose CFTR function and are highly susceptible to early, aggressive colorectal tumor development. Similarly, based on the Authors data, a CDK8/19 small molecule inhibitor might be expected to reduce CFTR expression in the intestine in vivo. This would have wider implications for many readers, concerning the concept of using CDK8/19i in vivo in future clinical applications. The manuscript would benefit if the Authors examine whether the CFTR becomes reduced in vivo in the intestine, in mice treated with CDK8/19i, or a CF-like phenotype in mouse intestine treated with a CDK8/19-inhibitor. In any case, the Authors should comment on this possibility given its wider implications for the use of CDK8/19 inhibitors in humans.

This is a very good suggestion, and we would have liked to have already done these experiments but we have only recently acquired a sufficient quantity of inhibitors to do so. We have now performed the suggested in vivo experiments with a well characterised and highly specific CDK8/19 inhibitor, Senexin-631, developed by Igor Roninson (University of South Carolina). The result was striking: we found that CDK8/19 inhibition for four days caused a significant increase in mucin staining in the intestine, as shown in new Fig 6. This effect wore off over time as at day 11 there was no longer any visible difference in mucin staining, indicating adaptation to the inhibitors. Interestingly, we found that there was a strong upregulation of CDK8 and CDK19 in response to long-term treatment with inhibitors, which may well tend to counteract the effect (and incidentally gives a very good biomarker for CDK8/19 inhibition in vivo). There was no acute toxicity of these inhibitors: the mice tolerated the inhibitors well with no other strong complications of CF observed (weight loss, intestinal occlusion, etc; see new Fig EV10). These results are important both with regards to the mechanism of mucin expression and secretion, which depends on CDK8/19 kinase activity, as well as regarding potential usefulness and toxicity of inhibitors. One potential advantage of CFTR inhibition could be to reduce acute fluid loss in secretory diarrhea (a major global health problem due to parasites and drug side-effects); as CDK8/19 inhibitors appear to work in the short term, at least, and can be well tolerated in vivo, future studies will address possible usefulness in this regard.

Reviewer2: The Authors have addressed this comment with new analyses and a clear conclusion.

Main Fig1, Fig2, and Fig4 are very clear.

Thank you for this appraisal.

Fig3: The Authors describe in the Results section that CDK8-KO intestinal epithelium shows almost no transcriptomic changes versus wild-type intestine (DEG = 4 genes). In contrast, CDK8-KO intestinal epithelium organoids show 716 up, 575 down, total DEGs = 1291 genes. Even with a 2x threshold, DEG's = 390 genes (Figure 3A). Can the Authors comment on this difference?

This is a good point and we are happy to elaborate. We suspected that the lack of statistically significant transcriptomic changes in the CDK8-KO intestinal epithelium (DEG = 4 genes in our original analysis) may well have more to do with the complexity and biological variability of the system, and the fact that we only had replicates. In such cases, the statistical power of the analysis of the in vivo RNA-seq is reduced. We have now investigated this hypothesis. The original transcriptome analysis was performed in 2017 using a pipeline that has since been superceded; we reanalysed the data applying the same pipeline as for the organoid RNA-seq and found 31 differentially expressed genes (see new Table EV1), even given the lack of additional replicates; indeed, the variance is limiting. Finally, to avoid any potential misinterpretation, we have modified the wording of this section: the fact that many genes in vivo do not pass a threshold p-value for differences being statistically significant does not necessarily mean that they are not deregulated; simply, that we do not have the statistical power to claim that they are.

Reviewer2: The Authors have addressed this comment with re-analyses and refinement of the conclusions.

Moreover, in the Discussion section, the Authors make a conclusion that needs clarification or qualification, as it appears at odds with the data: "Our results also do not support an essential role for Mediator kinases in general gene expression, in contrast to Mediator itself, since relatively few genes were highly deregulated in double knockouts." However, this only appears to apply to the in vivo intestinal tissue for CDK8-KO, but not the organoids for CDK8-KO or CDK8/19-dKO -can the Authors include this nuance in the Discussion, and comment on reasons for this large difference? In particular, the data does show a very large effect on gene expression for CDK8/19-double KO, where it appears > 3600 differential genes were observed (Figure 3B). Therefore the current statement seems out of place, and may require re-phrasing.

In the Discussion section we had indeed concluded that "Our results also do not support an essential role for Mediator kinases in general gene expression". Although perhaps this is open to misinterpretation (and has been re-phrased in our revised version), we meant to contrast it to what is observed when essential proteins of the Mediator complex (e.g. Med14) are removed. In this case, a global shift in the whole transcriptome occurs within 60 hours, with a mean downregulation of a factor of 7, as shown in:

El Khattabi, L., Zhao, H., Kalchschmidt, J., Young, N., Jung, S., Van Blerkom, P., ... & Casellas, R. (2019). A pliable mediator acts as a functional rather than an architectural bridge between promoters and enhancers. Cell, 178(5), 1145-1158.

Finally, even though we found 3182 differentially expressed genes in the CDK8/19 KO organoids, for the majority of these the fold-change is less than two, which may not be biologically very meaningful.

This has now been explained better.

Reviewer2: The Authors have addressed this comment, and clarified the associated text.

Can the Authors clarify if their RNAseq expts with a "spike-in" to detect a global shift in the whole transcriptome? (This is not mentioned in the Methods) RNAseq has an internal normalization that can hide a global shift in the whole transcriptome (Loven J et al., 2012, Cell, PMID: 23101621). This might apply in a situation like this manuscript where the core transcriptional machinery is being manipulated. It is perfectly acceptable if no spike-in was included, not everyone needs to do this, however it might offer an explanation for why few genes might be observed as differential in the case of the intestinal epithelium.

This is an astute comment, and we also worried (as one should when knocking out the only two kinases known to regulate Mediator) that we might normalise out a shift in the whole transcriptome. We thus employed ERCC spike-in RNAs for RNA-seq of the organoids, added in proportion to the genomic DNA isolated in parallel with the RNA (in case rRNA was also altered, we did not spike in proportion to total RNA). We compared results between library normalisation and spike-in normalisation, and there were no global differences (as shown in new Fig EV8A). The magnitude of the fold-change was generally slightly smaller for the spike-in normalisation (see figure below). This is almost certainly due to the high dispersion of spike-in normalisation factors for the WT samples, that range from 0.51 to 1.04 (average 0.8), compared to library normalisation factors, which vary from 0.97 to 1.00 (average 0.986). Library normalisation is therefore superior and we present these data.

Reviewer2: The Authors have addressed this comment satisfactorily with re-analysis.

The Authors mention in their Discussion section: "we found that there was a strong overlap between transcriptome changes of double knockout organoids and intestinal knockout of the gene encoding CFTR...." However I did not find the Figure reference for the comparison of transcriptomic changes in CDK8/19i vs CFTR-KO, as mentioned -can the Authors please clarify this, or show this analysis?

We apologise for not having shown this data, which is now presented in Venn diagram format in new Fig 4B and the data in a Supplementary Table EV3. This shows that 98% of genes deregulated in the small intestine in CFTR knockouts are also deregulated in CDK8/19 knockouts. Most CFTR KO upregulated genes are similarly deregulated in CDK8/19 knockouts, but only a minority of downregulated genes.

Reviewer2: The Authors have addressed this comment.

Minor Comments

Introduction: the statement: "CDK19 deletion has not yet been reported." I suggest to specify that this applies in vivo, highlighting the novelty of the new study. In vitro, CDK19 has been knocked out in a number of cell types.

While our paper was in revision, a competing lab published (Dannappel, JCI, Aug 25 2022) that mice obtained from the Jackson laboratory are "viable and generally healthy", and we cite this study.

Reviewer2: The Authors have addressed this comment.

In this sentence in the Results section, I think the word "as" might be missing. Otherwise it is not clear: "We found that Senexin B treatment for 24h leads to the downregulation of Cftr and upregulation of Muc3 expression (Fig 4G) "AS" seen upon genetic ablation of both Cdk8 and Cdk19, indicating that kinase activity of CDK8/19 controls CFTR pathway gene expression."

In this sentence in the Results section, I think the word "they" refers to "CDK8/19", but it could alternatively refer to "quiescent cells" -I suggest to re-phrase to clarify: "....(Koehler et al, 2016) our data suggest that in the intestinal epithelium, cells devoid of both CDK8 and CDK19 have an increased tendency to become quiescent, implying that they provide a growth advantage."

Thank you for pointing out these glitches; we have included all the modifications suggested.

Reviewer2: The Authors have addressed these comments.

Reviewer 3

Using conditional "floxed" KO mouse models and small intestinal (SI) organoids generated from these mice or gene edited by CRISPR/Cas9 to obtain conditional double KOs for the cyclin-dependent kinases CDK8 and CDK19, this study shows rather convincing evidence for a functional redundancy of these highly conserved kinases in the control of gene expression by the essential Mediator gene transcription complex. However, the combined deletion or pharmacological inhibition of CDK8 and

CDK19 in intestinal organoids is not lethal but reduces proliferation, results in loss of Cyclin C, and induces a cystic fibrosis-like phenotype, characterized by a partial loss of CFTR expression and function, and mucus (Muc2 and 3) accumulation and hypersecretion by goblet cells. The models used and the findings obtained in this study are robustly documented and are new and of considerable scientific and medical interest in the area of tumor biology and CF.
We thank the reviewer for this appreciation.

A major limitation is however its focus on a single species (adult mice, not humans) and tissue (small intestine).
It is of course true that the results would have even more impact if it could be shown that they apply to humans and / or airway tissue, but we felt it important to document our results in a first discovery paper involving nearly ten years of work, opening the way for exploring this further.

Reviewer2, in place of Reviewer3: I think it is reasonable to work in one species, the mouse model, in a single study. In my opinion, no further action required here.

Yet there are several points of criticism that require further attention.
Specific comments:
1. On p. 4, 13 l. up the authors justify the use of Cdk8/Cdk19 double KO intestinal organoids rather than mice as a model by claiming that Cdk8/Cdk19 double knockout in adult mice is most probably lethal. However, it is unclear why they did not attempt to generate Cdk8lox/lox/Cdk19lox/lox mice and to cross them with a Tamoxifen-inducible Cre expressed under control of the intestine-specific villin promoter. Assuming that the double KO would slow down intestinal cell proliferation but is not lethal and allows differentiation, as reported for the organoids, Tamoxifen treatment is not expected to induce a severe morbidity or lethality at the intestinal level on the short run. Because the intestinal phenotype of CF mice has been described in much detail in the literature (e.g. see Kreda et al Cold Spring Harb Perspect med 2012; 2:a009589; Ikpa et al Genomics 2020; 112: 1139), confirmation of the CF-like phenotype in double Cdk8/Cdk19 knockout mice in vivo rather than in organoids would further strengthen this study.

While it would indeed be ideal to have a double conditional CDK8/19 knockout, such a model did not exist and would take a long time for our lab to generate. Yet to address this issue, and also to examine the effect of inhibition of both CDK8 and CDK19 in vivo, we performed additional experiments in mice with the newest CDK8/19 inhibitor, which has been validated in vivo, Senexin 631 (Ding et al., PNAS, 2022). As described above in our response to suggestions of reviewer 2, we found two quite striking observations: the first is that CDK8/19 inhibition in vivo almost exactly recapitulated the phenotype of double knockout organoids in vitro, i.e. a strong increase in mucin staining, while the second is the strong compensatory upregulation of CDK8 and CDK19 protein levels (thus constituting a good in vivo marker for CDK8/19 inhibition). The latter observation may explain why the mucin phenotype was only transient, as it was observed at 4 days but no longer at 11 days, and it may have reduced the toxicity of the treatment, which was well tolerated.

Given the extreme conservation of these two kinases, the only kinases known to regulate Mediator, and the fact that double knockout organoids lose viability over time, we expected a lethal phenotype from intestine-specific double mutant mice. This is the main reason we did not cross our conditional Cdk8 mutant mice to the Cdk19 CRISPR mutant mouse, which was available from the Jackson institute. We also knew that the Firestein lab (Melbourne, Australia), with whom we were in touch, was performing such experiments and it did not make sense to duplicate efforts. The Firestein lab published their paper with exactly this model in JCI on August 25th 2022, i.e. while we were finishing revisions (Dannappel et al., 2022, J Clin. Inv.). Like us, they conclude that CDK8 and CDK19 are dispensable for cell proliferation and differentiation in the intestinal epithelium, although they did not study the CFTR pathway. However, they observed a reduction in the proportion of cells issued from the secretory lineage in double knockouts and interpreted this as being evidence for a lineage switch regulated by Mediator kinases. We find no such evidence and were surprised by what appears to be hyperplasia of secretory cell types in the intestinal epithelium of their wild-type mice, which show far more goblet cells and tuft cells per villus than are typically seen (c.f. Gerbe et al., 2016, Nature), possibly reflecting the presence of pathogens, whereas their Cdk8/Cdk19 knockouts show no such hyperplasia. Along with the absence of a change in the balance of secretory lineage cells in Cdk8/Cdk19 knockouts in our experiments, and the accumulating evidence that CDK8/19 are required for pro-inflammatory responses (Chen et al, 2017, PNAS; Johannessen et al, 2017, Nat Chem Biol) we think it is unlikely that Mediator kinases control a lineage switch in the intestine but plausible that they are required for mucosal immune responses. We discuss this in the revised version of our paper.

Reviewer2, in place of Reviewer3: The Authors have addressed this comment with a nuanced argument and updates to the manuscript that incorporate these points.

2. CDK8/19 deletion or Senexin B inhibition of the kinase activities in the SI organoids resulted in the gradual loss of CFTR expression, CFTR protein, and CFTR function, as measured by qRT-PCR, WB, and the organoid swelling (FIS) assay, respectively (Fig. 3, D,E; Fig. 4, E-G). However, these observations raise several questions: (i) (WT) CFTR transcripts are down by only ~25% in Cdk8 KO organoids and by ~50% in the double KOs (Fig. 3D). It is well known that a loss of 50% WT CFTR expression and activity as seen in CF carriers (CFTR+/severe mutation) does not cause any pathology and is beyond the dynamic window of the FIS assay. It is more likely, therefore, that CDK8/19 deletion results in a much more dramatic loss of

expression and function of other transporters or regulators required to allow the forskolin-activated, CFTR-dependent anion secretion measured by FIS, e.g. NKCC1, NBCe1, adenylyl cyclase or PK-A (please note that anion and fluid secretion in 3D organoids as monitored by FIS is also dependent on Cl- or HCO3- import across the basolateral membrane of the enterocytes and on AC and PKA activity). Therefore the authors need to verify in their RNA-Seq data base (Fig, 3B) whether one or more of these genes are strongly downregulated in the double KO mice;

This is an excellent suggestion, that we have implemented. We surmised that the 25% difference in mRNA, which takes into account both transcription and degradation, might be reflected in larger changes at the protein level. This involved acquiring new antibodies from the Cystic Fibrosis Foundation (University of North Carolina) which took some time (see reviewer's point (iii), below). We have thus performed new western blots to re-examine the levels of CFTR protein in Cdk8-/-/ Cdk19-/- organoids. The band migrating in WT organoids at the same height as the CFTR positive control is almost completely absent in the double knockout (as seen in the CFTR negative control; see new Fig. 4D). We also blotted intestinal tissue from our new experiment in which we treated mice with CDK8/19 inhibitor (see response to comment (i), above). Strikingly, the CFTR band is increased in intestinal tissue after 4d of treatment where the CF-like phenotype is seen, like the increase in CDK8/19 expression, suggesting a compensation (new Fig 6A).
Second, indeed, there are many solute carriers (95) whose expression is deregulated in CDK8/19 knockouts (new supplementary Table EV5), including four inorganic anion transporters of the Slc26 family, all of which are upregulated. NKCC1 (Slc12a2) is not identified but its putative inhibitor Slc12a9 is upregulated. We also do not identify NBCe1 (Slc4a4) as deregulated, but a homologue (Slc4a2) is upregulated. 22 solute carrier genes have reduced expression. Some of these changes may either contribute to or be compensatory for reduced CFTR pathway function. We have now discussed these results and modified the conclusions to avoid the appearance of making excessive claims that the phenotype is entirely and directly due to loss of CFTR expression, even though this remains a possibility.

Reviewer2, in place of Reviewer3: This is a good point to raise. The Authors have addressed this comment by analyzing the other transporters for alterations in expression, and incorporating their possible contribution to the phenotype, in the Conclusions. As such I suggest that this issue has been explored and addressed sufficiently.

(iii) (NB: we did not find the reviewer's "(ii)") Western blotting or ICH of rodent CFTR is notoriously difficult to perform because of a lack of specific monoclonal antibodies, and is at best semi-quantitative. It is doubtful whether the band stained by the monoclonal CFTR Ab2784 in Fig. 3E represents the C-band of complex-glycosylated CFTR, which normally is broad and very diffuse. Therefore the whole blot rather than a small section of the blot should be made accessible to the reviewer, and the identity of the band and the specificity of the antibody for mouse CFTR should be verified by WB of a Cftr-/- mouse. Also the number of blots examined should be given. Considering the 1363 genes that are downregulated in the double KO organoids (Fig. 3B), there are multiple candidate proteins that could possibly crossreact with the Abcam antibody;

We agree that these blots are technically difficult, especially from intestinal tissue. As explained above, we have acquired new CFTR antibodies as well as positive and negative controls and confirmed our results using these antibodies (new Fig 4D), which indeed show broad and rather diffuse staining. Overexposure of whole blots (right) shows that the minor bands are cross-reacting proteins as they are present in the negative control.

Reviewer2, in place of Reviewer3: The Authors have addressed this comment by displaying the full blot as requested and further analyses with new antibodies. To me, these questions have been explored sufficiently in the context of the current study.

(iv) a more direct assessment of CFTR activity in intestinal organoids can be achieved by electrical current measurements across monolayers of organoids permeabilized basolaterally by a pore-forming agent, e.g. nystatin.

This is a very interesting suggestion but we could not find collaborators who were available to help with such experiments, and we will have to defer this to a follow-up study.

Reviewer2, in place of Reviewer3: I agree, this is an interesting question. But perhaps it is beyond the scope of the current study, which explores several other avenues already. For me, the Authors have addressed this comment.

3. Strong downregulation of CFTR and upregulation of Muc2 in goblet cells is also seen upon differentiation of WT intestinal organoids by removing Wnt3a from the culture medium and/or by inhibiting Notch signaling by the -secretase inhibitor DAPT. Would it be possible, in analogy, that the KO of CDK8+19, or the inhibition of cyclin-dependent kinase activity by Senexin B results in the repression of genes in the Notch signaling pathway, explaining the partial inhibition of cell proliferation (Fig. 2F) and the conversion into a mucoid phenotype? Did pathway analysis of the RNA-Seq data showed downregulation of the Notch signaling components?

This is another excellent suggestion, especially since CDK8-cyclin C has already been implicated in control of the Notch pathway (Li et al., Nat Cell Biol, 2014). Yet loss of cyclin C was found to upregulate, rather than downregulate the pathway. In our RNA-seq and new qRT-PCR data from double knockout organoids, there was indeed, at the transcriptome level, a significant downregulation of Notch1 (see new Fig 3E), but Hes1, one of the main targets of Notch1, was not altered. Since these are population studies, we cannot rule out bigger changes in individual cell types such as goblet cells. Thus, it remains possible that altered activity of the Notch pathway might contribute to some of the phenotypes. Further single cell studies will be required to explore this in detail. This has been added to the discussion.

Reviewer2, in place of Reviewer3: I agree with Reviewer3 that Notch is a likely candidate for understanding some of the data in the current data. However, as the Authors note, a single cell analysis is likely to be required to reach a sold conclusion on the extent to which Notch signaling is up- or down-regulated in specific cell types. In my opinion, the Authors have addressed this comment sufficiently by including a discussion of the Notch pathway as a possible candidate for future research based on their current findings.

4. Fig. 3B: How do the authors explain that in the Venn diagram only 27 and 14 out of the 155 respectively 151 genes that are up-or downregulated in the Cdk19-/- organoids are also up-or downregulated in the double KO organoids? How can the additional knockdown of Cdk8 result in the loss of up-and downregulation of 128 (131 =129+2) respectively 137 (129 +8) genes?????

This is a very astute comment, and we have explored this further. We first ruled out several potential artefacts, whereby double knockouts might have greater variance than single knockouts for these particular genes. This is not the case for genes other than those grouping to very low variance: see graph in new Fig EV8B. We also ruled out a potential bias of these genes to low read counts; see graph in new Fig EV8C. We thus considered the possibility that some genes might be dominantly controlled by, or specific to, CDK8 or CDK19. As such, additional knockout of the second Mediator kinase might counteract the effect of the first knockout. Thus, we looked at read counts for each gene deregulated only in single Cdk8 or Cdk19 knockouts but not double knockouts. This revealed that essentially all genes specifically deregulated in single Cdk8 knockouts are also somewhat deregulated in the same direction in double knockouts, but to a lesser degree. In almost all cases, the same genes were either not deregulated in Cdk19 knockouts or somewhat deregulated in the opposite direction. Interestingly, the same is not true for genes only deregulated in single Cdk19 knockouts: the double knockouts more resemble single Cdk8 knockouts rather than showing an intermediate level of expression between the single and double knockouts. These data are presented in Principal Component Analysis graphs in new Fig EV8D and E. This analysis highlights the fact that the failure to pass a threshold for statistical significance for being deregulated is not synonymous with not being deregulated, a common misconception when interpreting gene expression data; and that gene expression networks are inherently complex: effects of combined knockouts are not always additive. Moreover, it suggests that CDK8 exerts a stronger influence on gene expression than CDK19 (consistent with the reported higher expression levels of CDK19), and that some genes are regulated in opposite directions by CDK8 and CDK19; i.e. although CDK8 and CDK19 appear largely redundant, some genes may be specifically regulated by one or the other kinase. As such, the suggestion was very useful and allowed us to strengthen message of the paper.

Reviewer2, in place of Reviewer3: As the Authors discuss, the roles of CDK and CDk19 may be synergistic, or antagonistic, in a gene-specific manner. The two kinases are also known to have distinct regulons. These features were previously explored in Galratih et al., 2013 Cell (PMID: 23746844), where these conclusions were reached. The Author´s current gene expression data on the single, or double-KO models is therefore not incompatible. Indeed, it is consistent with what is previously known for the complex interplay between CDK8 and CDK19. The Authors have addressed this comment.

5. Fig. 4F: The extent of FIS inhibition in the Cdk8-/-/Cdk19-/- mice (~80% of WT) was much higher than the inhibition by saturating concentrations (10 uM) of SenB (<40% of WT), suggesting that the loss of CDK proteins has more impact on CFTR function than the loss of CDK kinase activity. Therefore the claim that "the kinase activity of CDK8/19 controls CFTR pathway expression" (p. 5, 9 l. up) should be toned down.

This is a fair comment and we have revised the description of these results to be more cautious. It is worth mentioning that we showed that the effect of the inhibitor was not seen after only one hour, but was clearly visible after 24h, suggesting that it is a consequence of loss of transcription rather than a regulation of the pathway via direct phosphorylation by Cdk8/19. It is also worth mentioning that inhibitors are not "on/off" switches, we have to take into account that they are ATP competitive inhibitors and do not completely kill the kinase activity even at micromolar concentrations, since intracellular ATP is millimolar. In this context, using our new CFTR antibodies, we found that 24h Senexin B inhibition of CDK8/19 in organoids caused a much smaller reduction of CFTR expression than Cdk8/19 knockout (new Fig 5H).

Reviewer2, in place of Reviewer3: This is a fair comment. The Authors have addressed this comment by inserting a more nuanced commentary to these data.

6. Fig. 4G: MUC2, not MUC3 is the major secreted mucin in mouse intestine. Was the expression of the Muc2 gene also upregulated by SenB?

Our new qRT-PCR experiment (new Fig 3D) confirms that Muc2 is slightly upregulated in intestinal organoids upon Senexin B treatment, similarly to the double Cdk8/19 knockout.

Reviewer2, in place of Reviewer3: The Authors have addressed this comment.

7. To allow a fair comparison, was the genetic background of the WT mice or intestinal organoids always identical to the one of the KO mice/organoids?

Yes, the genetic background of all mice and organoids was identical; this has been mentioned in the revision.

Reviewer2, in place of Reviewer3: The Authors have addressed this comment.

8. In the Method section, the description of the CRISPR/Cas9 editing of the organoids on p. 8, ala. 3 is highly repetitive with the text on p. 13, ala. 2. Please correct this duplication.
9. Some minor typo's should be corrected, e.g. p. 13, l. 6: deferentially=differentially; p. 19, 4 l. up: umpaired=unpaired

Thank you for pointing out these errors, which have been corrected.

Reviewer2, in place of Reviewer3: The Authors have addressed these comments.

Cross-comments by referee 3:

On Referee 1:
- I am less concerned about the effectivity of Cdk8 ablation in vivo, considering the complete absence of CDK8 immunostaining in small intestine of Cdk8-/- mice collected two months after tamoxifen treatment (Fig. 1A).

Please also see our reply to referee 1.

- I agree that the relationship between CDK8/19 KO and the "CFTR pathway" is descriptive rather than mechanistic, and could be simply due to enhanced differentiation of the organoids (e.g. by Notch inhibition) which strongly reduces CFTR expression and function in intestinal organoids. As suggested in my report, it is even not certain that the inhibition of organoid swelling in the double KO is due to a loss of CFTR expression, more likely it results from loss of chloride import through NKCC1 or the loss of upstream signaling. Because the authors have bulk RNA Seq data, it is easy for them to explore such possibilities.

As described in our detailed reply, above, we have explored these possibilities and report the data in the revised version. Please also see our comment in the general reply to reviewer 1 about previous claims in the literature about the mechanisms of action of CDK8/19 on gene expression, which, in our view, do not genuinely constitute convincing mechanisms.

Reviewer2, in place of Reviewer3: The Authors have addressed these comments by exploring the points and adjusting the manuscript text to be more cautious. To me, the Authors have sufficiently addressed these concepts.

On Referee 2
- I agree with his/her positive and negative comments, in particular with major comment 1 and 3. It remains possible that CDK8/19 KO or inhibition of the kinases in organoids would only turn undifferentiated, crypt-like and CFTR-rich organoids into differentiated, villus-like organoids and therefore would not trigger a CF-like phenotype in mice in vivo. This might also explain why CDK8 KO in organoids but not in mice in vivo causes major changes in gene expression. The in vivo double KO could be made chemically by treatment with CDK8/19i or senexin B (but this will also affect multiple other tissues), or, preferentially, genetically by generating conditional double KO mice (cf. my report, comment 1). But perhaps the authors have already tried one or more of these approaches and have strong arguments why intestinal organoids are the best/only usable models.

As described above, we have now used CDK8/19 inhibitors in vivo, which recapitulate the increased Mucin phenotype until adaptation occurs, and characterised better the WT and mutant organoids in terms of cell types. While some data suggest slight differences in stem cell and differentiation markers, these are not sufficient to state that these kinases promote the maintenance of an undifferentiated state.

Reviewer2, in place of Reviewer3: The Authors have addressed these comments with their nee assays, as described above.

Reviewer#1 Specific comment#1: The Authors may be able to address this relatively easily. Perhaps a published ChIP-seq of CDK8/19 might reveal binding proximal to the CTFR genes, providing a mechanistic direct link to the expression of CFTR?

We have looked at this but not found any evidence for it; please also see our reply to reviewer 1 on the ChIP-seq question.

Reviewer2, in place of Reviewer3: The Authors have addressed this comment.

Reviewer#1 Specific comment#2: The Reviewer is concerned that CDK8/19-dKO organoids might show a CF-like phenotype due to indirect effects, such as decreased proliferation or abnormal differentiation of different lineages. The Authors already show no effect on intestine cell composition or proliferation by CDK8-KO, in Fig.1. The Authors could perhaps extend this analysis into intestinal organoid cell composition, and using a CDK8/19-dKO context, using IHC or IF to characterize the major cell types present, and proliferation (ki67). The existing RNAseq might also indicate whether there is decreased proliferation or abnormal differentiation of different lineages. Related: It has been shown that CFTR null mice have increased (rather than decreased) epithelial cell proliferation (PMID: 11518680).

As explained in detail above, we have performed the suggested analysis, and we have also cited the suggested paper.

Reviewer2, in place of Reviewer3: The Authors have addressed these comments.

Reviewer#1 Specific comment#3-to-end: These concerns of the Reviewer may require only clarification or explanations by the Authors. This could be textual.

Indeed, as described above, we have clarified these points and present the analysis of organoid proliferation in a way that should be easier to understand.

Reviewer2, in place of Reviewer3: The Authors have addressed these comments.

Cross-referee comments from Reviewer#2 on Reviewer#3

Reviewer#3 Specific comment#1: Perhaps the Authors could easily address this point by one or two approaches; (i) providing any more information if they have attempted to make CDK8/19-dKO mouse intestine. The Authors say they anticipate that CDK8/19-dKO in intestine might be lethal -can they confirm that they tried this or elaborate on why they think it would be lethal? (ii) If the Authors have treated WT mice with CDK8/19i small molecule inhibitors, did they see lethality?

We have elaborated on why we thought intestinal double knockout mice would be lethal, and have performed experiments using WT mice with CDK8/19 inhibitors, as described above.

Reviewer#3 Specific comment#2+3: The Reviewer has a main concerned that CDK8/19-dKO might affect the expression of many ion channels, so that the CF-like phenotype observed is not solely dependent on CFTR. Perhaps the Authors could address this point by rescuing CFTR expression. If CFTR expression levels are returned to normal in the CDK8/19-dKO organoids, does this rescue the CF-like phenotype. I suggest that this might address this Reviewer concern, and provide further support to a central message of the paper. CFTR expression levels could be supported by exogenous over-expression, or CRISPRa of the endogenous CFTR, for example, in CDK8/19-dKO organoids, or WT organoids treated with CDK8/19i.

These are good suggestions, but we think that the idea of reviewer #3 is very likely and that the phenotype might not solely be due to CFTR downregulation. As such, we have explored contributions of other genes to the CF phenotype, as described above, and modified the conclusions of the paper accordingly. Rescue experiments to ensure delivery of ≈ 5kb of cDNAs to all cells of mutant organoids that do not proliferate well would be technically extremely challenging.

Reviewer2: The Authors should include in their conclusions that other transporters might contribute to the observed phenotype, in addition to the CFTR. In this way, they address this concept sufficiently.

Reviewer#3 Specific comment#4-9: these concerns of the Reviewer may require only clarification/explanations by the Authors. This could be textual.
Or in the case of antibody specificity for CFTR -the Authors could perform some reasonable check and controls on the antibody specificity, as the Reviewer#1 suggests.

As detailed above, we have acquired other antibodies and controls using CFTR mutant tissue and performed new analyses.

Reviewer2: The Authors have addressed these comments.

Dear Esther,
Please find enclosed the final version of our article “CDK8 and CDK19 act redundantly to control the CFTR pathway in the intestinal epithelium”, by Prieto et al (n°: EMBOR-2021-54261V4). We thank the reviewers for their time and thoroughness in assessing the revision.
We have addressed the remaining two comments:

Reviewer #1: Minor observation: Page 5.: "We performed RNA-sequencing from the intestinal epithelium of wild-type and knockout mice, but could identify only 31 genes with statistically significant expression alterations due to biological variability between animals and the limited number of replicates". I find a bit unusual to conclude in these first sentences that the only genes with statistical differences are not real hits.

Yes, indeed! We have changed the wording to: “We performed RNA-sequencing from the intestinal epithelium of wild-type and knockout mice, but, due to biological variability between animals and the limited number of replicates, could identify only 31 genes with statistically significant expression alterations (Table EV1). This nevertheless suggests that effects of CDK8 loss on gene expression may be somewhat restricted.”

Reviewer2: The Authors should include in their conclusions that other transporters might contribute to the observed phenotype, in addition to the CFTR. In this way, they address this concept sufficiently.

In the discussion we have the following conclusion: “While changes in FIS and in CFTR expression are consistent with CFTR being directly involved in the phenotype, we cannot at this stage conclude that CFTR is the sole player. Indeed, there are 95 solute carriers whose expression is deregulated in CDK8/19 knockouts (Table EV2), including four inorganic anion transporters of the Slc26 family, all of which are upregulated, and which might contribute to the phenotype”.

We have also implemented all of the editorial requests.
Best regards
Daniel

Dr. Daniel Fisher
National Centre for Scientific Research, CNRS
Montpellier Institute of Molecular Genetics, IGMM
1919 Route de Mende
Montpellier 34293
France

Dear Daniel,

I am very pleased to accept your manuscript for publication in the next available issue of EMBO reports. Thank you for your contribution to our journal.

At the end of this email I include important information about how to proceed. Please ensure that you take the time to read the information and complete and return the necessary forms to allow us to publish your manuscript as quickly as possible.

As part of the EMBO publication's Transparent Editorial Process, EMBO reports publishes online a Review Process File to accompany accepted manuscripts. As you are aware, this File will be published in conjunction with your paper and will include the referee reports, your point-by-point response and all pertinent correspondence relating to the manuscript.

If you do NOT want this File to be published, please inform the editorial office within 2 days, if you have not done so already, otherwise the File will be published by default [contact: emboreports@embo.org]. If you do opt out, the Review Process File link will point to the following statement: "No Review Process File is available with this article, as the authors have chosen not to make the review process public in this case."

Thank you again for your contribution to EMBO reports and congratulations on a successful publication. Please consider us again in the future for your most exciting work.

Best regards,
Esther

Esther Schnapp, PhD
Senior Editor
EMBO reports

****

THINGS TO DO NOW:

Please note that you will be contacted by Wiley Author Services to complete licensing and payment information. The required 'Page Charges Authorization Form' is available here: https://www.embopress.org/pb-assets/embo-site/er_apc.pdf - please download and complete the form and return to embopressproduction@wiley.com

EMBO Press participates in many Publish and Read agreements that allow authors to publish Open Access with reduced/no publication charges. Check your eligibility: https://authorservices.wiley.com/author-resources/Journal-Authors/open-access/affiliation-policies-payments/index.html

You will receive proofs by e-mail approximately 2-3 weeks after all relevant files have been sent to our Production Office; you should return your corrections within 2 days of receiving the proofs.

Please inform us if there is likely to be any difficulty in reaching you at the above address at that time. Failure to meet our deadlines may result in a delay of publication, or publication without your corrections.

All further communications concerning your paper should quote reference number EMBOR-2021-54261V4 and be addressed to emboreports@wiley.com.

Should you be planning a Press Release on your article, please get in contact with emboreports@wiley.com as early as possible, in order to coordinate publication and release dates.

EMBO Press Author Checklist

Corresponding Author Name: Daniel Fisher	
Journal Submitted to: EMBO Reports	
Manuscript Number: EMBOR-2021-54261V4	

USEFUL LINKS FOR COMPLETING THIS FORM
The EMBO Journal - Author Guidelines
EMBO Reports - Author Guidelines
Molecular Systems Biology - Author Guidelines
EMBO Molecular Medicine - Author Guidelines

Reporting Checklist for Life Science Articles (updated January 2022)
This checklist is adapted from Materials Design Analysis Reporting (MDAR) Checklist for Authors. MDAR establishes a minimum set of requirements in transparent reporting in the life sciences (see Statement of Task: 10.31222/osf.io/9sm4x). Please follow the journal's guidelines in preparing your manuscript.
Please note that a copy of this checklist will be published alongside your article.

Abridged guidelines for figures
1. Data
The data shown in figures should satisfy the following conditions:
- the data were obtained and processed according to the field's best practice and are presented to reflect the results of the experiments in an accurate and unbiased manner.
- ideally, figure panels should include only measurements that are directly comparable to each other and obtained with the same assay.
- plots include clearly labeled error bars for independent experiments and sample sizes. Unless justified, error bars should not be shown for technical replicates.
- if n<5, the individual data points from each experiment should be plotted. Any statistical test employed should be justified.
- Source Data should be included to report the data underlying figures according to the guidelines set out in the authorship guidelines on Data Presentation.

2. Captions
Each figure caption should contain the following information, for each panel where they are relevant:
- a specification of the experimental system investigated (eg cell line, species name).
- the assay(s) and method(s) used to carry out the reported observations and measurements.
- an explicit mention of the biological and chemical entity(ies) that are being measured.
- an explicit mention of the biological and chemical entity(ies) that are altered/varied/perturbed in a controlled manner.
- the exact sample size (n) for each experimental group/condition, given as a number, not a range;
- a description of the sample collection allowing the reader to understand whether the samples represent technical or biological replicates (including how many animals, litters, cultures, etc.).
- a statement of how many times the experiment shown was independently replicated in the laboratory.
- definitions of statistical methods and measures:
  - common tests, such as t-test (please specify whether paired vs. unpaired), simple χ2 tests, Wilcoxon and Mann-Whitney tests, can be unambiguously identified by name only, but more complex techniques should be described in the methods section;
  - are tests one-sided or two-sided?
  - are there adjustments for multiple comparisons?
  - exact statistical test results, e.g., P values = x but not P values < x;
  - definition of 'center values' as median or average;
  - definition of error bars as s.d. or s.e.m.

Please complete ALL of the questions below.
Select "Not Applicable" only when the requested information is not relevant for your study.

Materials

Newly Created Materials	Information included in the manuscript?	In which section is the information available? (Reagents and Tools Table, Materials and Methods, Figures, Data Availability Section)
New materials and reagents need to be available; do any restrictions apply?	Not Applicable	

Antibodies	Information included in the manuscript?	In which section is the information available? (Reagents and Tools Table, Materials and Methods, Figures, Data Availability Section)
For **antibodies** provide the following information:<br>- Commercial antibodies: RRID (if possible) or supplier name, catalogue number and or/clone number<br>- Non-commercial: RRID or citation	Yes	Materials and Methods

DNA and RNA sequences	Information included in the manuscript?	In which section is the information available? (Reagents and Tools Table, Materials and Methods, Figures, Data Availability Section)
**Short novel DNA or RNA including primers, probes**: provide the sequences.	Yes	Materials and Methods

Cell materials	Information included in the manuscript?	In which section is the information available? (Reagents and Tools Table, Materials and Methods, Figures, Data Availability Section)
**Cell lines:** Provide species information, strain. Provide accession number in repository **OR** supplier name, catalog number, clone number, and/**OR** RRID.	Not Applicable	
**Primary cultures:** Provide species, strain, sex of origin, genetic modification status.	Yes	Materials and Methods
Report if the cell lines were recently **authenticated** (e.g., by STR profiling) and tested for mycoplasma contamination.	Not Applicable	

Experimental animals	Information included in the manuscript?	In which section is the information available? (Reagents and Tools Table, Materials and Methods, Figures, Data Availability Section)
**Laboratory animals or Model organisms:** Provide species, strain, sex, age, genetic modification status. Provide accession number in repository **OR** supplier name, catalog number, clone number, **OR** RRID.	Yes	Materials and Methods
**Animal observed in or captured from the field:** Provide species, sex, and age where possible.	Not Applicable	
Please detail **housing and husbandry conditions**.	Yes	Materials and Methods

Plants and microbes	Information included in the manuscript?	In which section is the information available? (Reagents and Tools Table, Materials and Methods, Figures, Data Availability Section)
**Plants:** provide species and strain, ecotype and cultivar where relevant, unique accession number if available, and source (including location for collected wild specimens).	Not Applicable	
**Microbes:** provide species and strain, unique accession number if available, and source.	Not Applicable	

Human research participants	Information included in the manuscript?	In which section is the information available? (Reagents and Tools Table, Materials and Methods, Figures, Data Availability Section)
If collected and within the bounds of privacy constraints report on age, sex and gender or ethnicity for all study participants.	Not Applicable	

Core facilities	Information included in the manuscript?	In which section is the information available? (Reagents and Tools Table, Materials and Methods, Figures, Data Availability Section)
If your work benefited from core facilities, was their service mentioned in the acknowledgments section?	Yes	Acknowledgements

Design

**Study protocol**	**Information included in the manuscript?**	**In which section is the information available?** (Reagents and Tools Table, Materials and Methods, Figures, Data Availability Section)
If study protocol has been **pre-registered, provide DOI in the manuscript**. For clinical trials, provide the trial registration number **OR** cite DOI.	Not Applicable	
Report the **clinical trial registration number** (at ClinicalTrials.gov or equivalent), where applicable.	Not Applicable	

**Laboratory protocol**	**Information included in the manuscript?**	**In which section is the information available?** (Reagents and Tools Table, Materials and Methods, Figures, Data Availability Section)
Provide DOI OR other citation details if **external detailed step-by-step protocols** are available.	Not Applicable	

**Experimental study design and statistics**	**Information included in the manuscript?**	**In which section is the information available?** (Reagents and Tools Table, Materials and Methods, Figures, Data Availability Section)
Include a statement about **sample size** estimate even if no statistical methods were used.	Yes	The sample size was chosen based on G*power calculations for statistical power. We used an 80% power for detecting a 10% difference in the slope of curves with a confidence >95%, using a bivariate linear regression.
Were any steps taken to minimize the effects of subjective bias when allocating animals/samples to treatment (e.g. **randomization procedure**)? If yes, have they been described?	Yes	No randomisation procedures were used
Include a statement about **blinding** even if no blinding was done.	Yes	No blinding was done
Describe **inclusion/exclusion criteria** if samples or animals were excluded from the analysis. Were the criteria pre-established?<br><br>If sample or data points were omitted from analysis, report if this was due to attrition or intentional exclusion and provide justification.	Yes	Animals were not excluded from the analysis
For every figure, are **statistical tests** justified as appropriate? Do the data meet the assumptions of the tests (e.g., normal distribution)? Describe any methods used to assess it. Is there an estimate of variation within each group of data? Is the variance similar between the groups that are being statistically compared?	Yes	All statistical tests were reported in Figure legends. No normality distribution tests were performed

**Sample definition and in-laboratory replication**	**Information included in the manuscript?**	**In which section is the information available?** (Reagents and Tools Table, Materials and Methods, Figures, Data Availability Section)
In the figure legends: state number of times the experiment was **replicated** in laboratory.	Yes	Figure legends
In the figure legends: define whether data describe **technical or biological replicates**.	Yes	Figure legends

Ethics

**Ethics**	**Information included in the manuscript?**	**In which section is the information available?** (Reagents and Tools Table, Materials and Methods, Figures, Data Availability Section)
Studies involving **human participants**: State details of **authority granting ethics approval** (IRB or equivalent committee(s), provide reference number for approval.	Not Applicable	
Studies involving **human participants**: Include a statement confirming that **informed consent** was obtained from all subjects and that the experiments conformed to the principles set out in the WMA Declaration of Helsinki and the Department of Health and Human Services Belmont Report.	Not Applicable	
Studies involving **human participants:** For publication of **patient photos**, include a statement confirming that consent to publish was obtained.	Not Applicable	
Studies involving experimental **animals**: State details of **authority granting ethics approva**l (IRB or equivalent committee(s), provide reference number for approval. Include a statement of compliance with ethical regulations.	Yes	Materials and Methods
Studies involving **specimen and field samples:** State if relevant **permits** obtained, provide details of authority approving study; if none were required, explain why.	Not Applicable	

**Dual Use Research of Concern (DURC)**	**Information included in the manuscript?**	**In which section is the information available?** (Reagents and Tools Table, Materials and Methods, Figures, Data Availability Section)
Could your study fall under dual use research restrictions? Please check biosecurity documents and list of **select agents and toxins** (CDC): https://www.selectagents.gov/sat/list.htm	Not Applicable	
If you used a select agent, is the security level of the lab appropriate and reported in the manuscript?	Not Applicable	
If a study is subject to dual use research of concern regulations, is the name of the **authority granting approval and reference number** for the regulatory approval provided in the manuscript?	Not Applicable	

Reporting

The MDAR framework recommends adoption of discipline-specific guidelines, established and endorsed through community initiatives. Journals have their own policy about requiring specific guidelines and recommendations to complement MDAR.

**Adherence to community standards**	**Information included in the manuscript?**	**In which section is the information available?** (Reagents and Tools Table, Materials and Methods, Figures, Data Availability Section)
State if relevant guidelines or checklists (e.g., **ICMJE, MIBBI, ARRIVE, PRISMA**) have been followed or provided.	Yes	Materials and Methods
For **tumor marker prognostic studies,** we recommend that you follow the **REMARK** reporting guidelines (see link list at top right). See author guidelines, under 'Reporting Guidelines'. Please confirm you have followed these guidelines.	Not Applicable	
For **phase II and III randomized controlled trials**, please refer to the **CONSORT** flow diagram (see link list at top right) and submit the CONSORT checklist (see link list at top right) with your submission. See author guidelines, under 'Reporting Guidelines'. Please confirm you have submitted this list.	Not Applicable	

Data Availability

**Data availability**	**Information included in the manuscript?**	**In which section is the information available?** (Reagents and Tools Table, Materials and Methods, Figures, Data Availability Section)
Have **primary datasets** been deposited according to the journal's guidelines (see 'Data Deposition' section) and the respective accession numbers provided in the Data Availability Section?	Yes	Materials and Methods
Were **human clinical and genomic datasets** deposited in a public access-controlled repository in accordance to ethical obligations to the patients and to the applicable consent agreement?	Not Applicable	
Are **computational models** that are central and integral to a study available without restrictions in a machine-readable form? Were the relevant accession numbers or links provided?	Not Applicable	
If publicly available data were reused, provide the respective **data citations in the reference list**.	Yes	Reference list
